# New Biological Insights Into How Deforestation in Amazonia Affects Soil Microbial Communities Using Metagenomics and Metagenome-Assembled Genomes

**DOI:** 10.3389/fmicb.2018.01635

**Published:** 2018-07-23

**Authors:** Marie E. Kroeger, Tom O. Delmont, A. M. Eren, Kyle M. Meyer, Jiarong Guo, Kiran Khan, Jorge L. M. Rodrigues, Brendan J. M. Bohannan, Susannah G. Tringe, Clovis D. Borges, James M. Tiedje, Siu M. Tsai, Klaus Nüsslein

**Affiliations:** ^1^Department of Microbiology, University of Massachusetts Amherst, Amherst, MA, United States; ^2^Department of Medicine, University of Chicago, Chicago, IL, United States; ^3^Josephine Bay Paul Center, Marine Biological Laboratory, Woods Hole, MA, United States; ^4^Institute of Ecology and Evolution, University of Oregon, Eugene, OR, United States; ^5^Center for Microbial Ecology, Michigan State University, East Lansing, MI, United States; ^6^Department of Land, Air, and Water Resources, University of California, Davis, Davis, CA, United States; ^7^DOE Joint Genome Institute, Walnut Creek, CA, United States; ^8^Centro de Energia Nuclear na Agricultura, University of São Paulo, Piracicaba, Brazil

**Keywords:** Amazon rainforest soil, land-use change, metagenome assembled genomes, rare biosphere, soil metagenomics

## Abstract

Deforestation in the Brazilian Amazon occurs at an alarming rate, which has broad effects on global greenhouse gas emissions, carbon storage, and biogeochemical cycles. In this study, soil metagenomes and metagenome-assembled genomes (MAGs) were analyzed for alterations to microbial community composition, functional groups, and putative physiology as it related to land-use change and tropical soil. A total of 28 MAGs were assembled encompassing 10 phyla, including both dominant and rare biosphere lineages. Amazon Acidobacteria subdivision 3, Melainabacteria, Microgenomates, and Parcubacteria were found exclusively in pasture soil samples, while Candidatus Rokubacteria was predominant in the adjacent rainforest soil. These shifts in relative abundance between land-use types were supported by the different putative physiologies and life strategies employed by the taxa. This research provides unique biological insights into candidate phyla in tropical soil and how deforestation may impact the carbon cycle and affect climate change.

## Introduction

The Amazon Basin contains the largest continuous tropical rainforest on the planet (Laurance et al., 2001) and is home to unparalleled levels of plant and animal diversity (Da Silva et al., 2005). Rainforests in this region are under threat from human activities, with the largest single threat being conversion to agriculture (Soares-Filho et al., 2006). Several studies have illustrated that human activities in the tropics such as land use change have adverse effects on macro-organismal biodiversity (Dale et al., 1994; McKinney and Lockwood, 1999; Sala et al., 2000), and more recently it became apparent that belowground microbial communities are altered as well (Rodrigues et al., 2013; Paula et al., 2014; Ranjan et al., 2015; Meyer et al., 2017). Soil microbial biodiversity in the Amazonian tropics is primarily known through 16S rRNA and ITS gene sequencing surveys (Borneman and Triplett, 1997; Peixoto et al., 2002; Jesus et al., 2009; Tripathi et al., 2012; Mueller et al., 2014), with only limited genomic and metagenomic surveys (Navarrete et al., 2015; Meyer et al., 2017).

Phylogenetic analyses based on 16S rRNA gene sequences organize bacterial and archaeal life into more than 60 phyla. Half of these phyla have no cultured representatives, yet these ‘candidate’ phyla represent a substantial portion of the tree of life (Hugenholtz, 2002; Rinke et al., 2013; Hug et al., 2016). Until recently, these microorganisms have only been detected through surveys of 16S rRNA gene surveys of environmental sequencing (Dojka et al., 2000; Hedlund et al., 2014), which provided very little direct information about their ecology or metabolic capabilities. With the development of shotgun metagenomics, insights into the diversity and functional potential of microbial communities have drastically increased. Expanding on that advance, genome-resolved metagenomics (Alneberg et al., 2014; Imelfort et al., 2014; Eren et al., 2015; Kang et al., 2015; Sangwan et al., 2016) and single-cell genome sequencing (Gawad et al., 2016) were developed, and can now be used to characterize the genomic content of microbial populations without the need for cultivation. Metagenome-assembled genomes (MAGs) have been characterized from a variety of environments, including recently from soil (Wrighton et al., 2012; Brown et al., 2015; Delmont et al., 2015; Sekiguchi et al., 2015; Butterfield et al., 2016; Hu et al., 2016; White et al., 2016). By integrating large sequencing datasets from environmental samples into reconstructed genomic composition, MAGs have increased the resolution at which microbial ecologists can understand a microorganism’s functional potential.

The use of MAGs has increased our understanding of candidate phyla in the environment (Siegl et al., 2011; Wrighton et al., 2012; Kantor et al., 2013; McLean et al., 2013; Probst et al., 2017). A study by Brown et al. (2015) identified a distinct radiation from known bacteria that altered our view of the tree of life and was termed the candidate phyla radiation (CPR) (Hug et al., 2016). Altogether, the CPR lineages might encompass over 70 phyla and two superphyla, Microgenomates, and Parcubacteria (Anantharaman et al., 2016). CPR genomes are generally streamlined (<1.5 Mb) with limited biosynthetic capabilities suggesting that they rely on other organisms (micro- or macro-) for their essential or additional metabolic needs (Rinke et al., 2013; Kantor et al., 2013; Brown et al., 2015; Probst et al., 2017). Until now, most CPR genomes have been characterized from within subsurface environments, limiting our understanding of this major bacterial group in highly heterogeneous environments such as soil.

Soil is regarded as one of the most diverse ecosystems on Earth, and characterizing even the most abundant MAGs has been considered an outstanding challenge due to the exceptional levels of soil biodiversity (Gans et al., 2005; Howe et al., 2014; Prosser, 2015; White et al., 2016). Few studies have successfully performed genome-resolved metagenomics on soil and among those, some have perturbed the community composition prior to sampling to lower the complexity of the microbial communities (Delmont et al., 2015; Yeoh et al., 2015), while others have assembled MAGs without manipulation (Butterfield et al., 2016; White et al., 2016; Tas et al., 2018). Despite these recent metagenomic accomplishments, the genomic content of most microbial populations inhabiting soil from the Amazon basin and elsewhere have yet to be characterized.

Here, we analyzed ten extensive tropical soil metagenomes (five from pristine rainforest soil and five from an adjacent cattle pasture) to understand the microbial community composition and how deforestation may affect these communities. In addition, we also characterized 28 MAGs from the rare soil biosphere for their functional potential in these tropical rainforest soils, because there is no knowledge of the metabolic capabilities of these groups either in any soil (Microgenomates and Parcubacteria) or in tropical soils (Rokubacteria). We also described shifts in the soil chemistry, and in the soil microbial functional potential. The sequencing depth of our dataset (6.4 billion shotgun metagenomic reads) was critical in the successful reconstruction of microbial genomes from this highly complex biological system. We were able to characterize MAGs encompassing 10 phyla, including CPR superphyla Microgenomates, and Parcubacteria, and from ubiquitous soil bacteria, Acidobacteria. Using comparative genomic approaches, we elucidated the unique features and potential metabolic capabilities of various tropical soil microbial populations. Not only does our study begin to depict the genomic and functional potential of poorly understood phyla, but it also increases our understanding of understudied tropical microbial biodiversity.

## Materials and Methods

### Site Description, Sampling, and Soil Chemistry

This study was performed at the Amazon Rainforest Microbial Observatory site, established in 2009 (10°10′5″ S and 62°49′27″ W). Sample design and soil chemical analyses were performed as specified previously (Rodrigues et al., 2013; Meyer et al., 2017). Briefly, in April 2010 soil cores collected from primary rainforest and a 38-year-old converted pasture (five each). The pasture site was initially a pristine rainforest. After selective logging of timber trees all remaining vegetation was cut at the end of the raining season and left to dry. At the end of the 3 months long dry season the remaining biomass was burned. The pasture site was then aerially seeded with *Urochloa brizantha*. No herbicides, tillage, or chemical fertilizers are used in this process. Weeds are controlled by fire when necessary (roughly every 2 years). There are many soil studies at Fazenda Nova Vida ranch in Rondonia and overall the pasture soil has an increase in soil bulk density especially in the top 0–5 cm of soil. The soil type for both pasture and forest sites are Ultisols (yellow–red podzolic soils) (de Moraes et al., 1996). Soil cores were sampled to a depth of 10 cm using 5 cm diameter PVC tubes and homogenized. Soil cores were frozen on the spot, transported on dry ice to the laboratory and stored at −80°C until further processing for DNA extraction. Sub-samples of each soil, stored at 4°C, were also analyzed for pH, total C and N, and other elemental analyses as described previously (Rodrigues et al., 2013).

### Nucleic Acid Extraction and Sequencing

Soil DNA was extracted following the same protocol as Rodrigues et al. (2013). DNA was sequenced as described in Meyer et al. (2017) at the United States Department of Energy – Joint Genome Institute (JGI) on the Illumina HiSeq platform across 21 lanes to produce 6,366,557,730 paired-end reads of 150 bp.

### Bioinformatic and Statistical Analysis of Metagenomes

Raw reads were uploaded to MG-RAST (Meyer et al., 2008). Then, functional and taxonomic annotations were obtained as described in detail by Meyer et al. (2017), except that in this study the sequences were not rarefied. Instead, the raw annotation counts for both function and taxonomy were analyzed using DESeq2 package in R version 3.4.2 (Love et al., 2014; R Core Team, 2016). Microbial community cluster analysis and visualization were done using the vegan R package (R Core Team, 2016; Oksanen et al., 2018).

### Metagenomic Assembly

All raw reads of metagenome samples were quality trimmed using fastq-mcf (Aronesty, 2011) and paired-end reads assembled using FLASH (Magoč and Salzberg, 2011) as described in Guo et al. (2016). The reads were pooled and assembled with MEGAHIT (Li et al., 2015) (−m 1.38e^12^ −t 32 −l 400 –k-mins 35 –k-max 75 –cpu-only), which used 42.4% of all reads resulting in 36,786,646 contigs with a total length of ∼27 Gbp. For the genome reconstruction, we only used contigs longer than 5 kb decreasing the total contigs to 79,226 and total nucleotides to 627 Mb. The number of reads used to map each sample to the filtered metagenome co-assembly is detailed in Supplementary Table S1.

### Genome Reconstruction and Annotation

All genome reconstructions followed the protocol outlined in detail by Eren et al. (2015). Briefly, quality filtered reads for each metagenome were mapped to the co-assembly of short reads (>5 kb) using bowtie2 with default parameters (Langmead and Salzberg, 2012). The sequence alignment map (SAM) files were converted to a binary format (BAM) that were then imported into the Anvi’o 2.0 pipeline along with the metagenomic co-assembly for genomic binning using sequence composition and differential mean coverage (Li et al., 2009; Eren et al., 2015). An initial binning using CONCOCT (40 clusters) was performed (Alneberg et al., 2014) followed by human-guided manual binning and curation of MAGs using the anvi’o interactive interface (see Eren et al., 2015 for details). MAGs were annotated using PATRIC (Wattam et al., 2014). The completion of the genome and the redundancy percentage (i.e., more than one single copy gene is present) for the genomic bins was determined using the 139 single copy genes used in anvi’o except for the MAGs designated as Microgenomates or Parcubacteria, which used the 44 single copy genes outlined by Brown et al. (2015). The unique features found in each MAG were determined using the PATRIC FIGFam protein family sorter. The features were then checked against reference genomes to ensure they could not be found. For each phylum or order, there is a different size set of reference genomes; 186 for Acidobacteria, 55 for Melainabacteria, 75 for Rokubacteria, 859 for Microgenomates, 4 for Pacebacteria, and 236 for Parcubacteria. The potential metabolism of the organisms based on the MAG annotations was determined by the PATRIC comparative pathway tool, by searching for specific functional annotations, and using BLAST (Altschul et al., 1997). All NCBI genome comparisons were completed in PATRIC by searching for the genomes, creating a genome group, and comparing the genomes in the protein family sorter with FIGFams and the comparative pathway tool. The new genome reporting standards and metadata for MAGS as outlined by Bowers et al. (2017) can be found in Supplementary Table S2. tRNAscan-SE v.2.0 was used to determine the number of tRNAs in all MAGs, except Amazon_FNV_2010_0_2_1 and Amazon_FNV_2010_0_4 (Lowe and Chan, 2016). These two MAGs used Aragorn to check for tRNAs due to large file sizes that tRNAscan-SE online could not process (Laslett and Canback, 2004). CheckM was used to determine if 16S or 18S was present in each MAG (Parks et al., 2015). Lateral gene transfer (lgt) in the Amazon Rokubacteria MAG was predicted using CompareM lgt-di function with default settings^1^. The heatmap visualizing the absolute abundance of each MAG in each metagenome sample was made using the heatmap.2 from the gplots package in R (R Core Team, 2016; Warnes et al., 2016).

### Pangenome Analysis

All pangenomic analyses were performed in the anvi’o 2.0 workflow for microbial pangenomes using the NCBI-blast analysis^2^. The “core” included protein clusters detected in at least 66% of the genomes. Protein clusters only detected in one genome were considered “unique.”

### Phylogenetic Analysis

All phylogenetic analyses were performed using MEGA 7.0 (Kumar et al., 2016) using a modified protocol from Brown et al. (2015). Briefly, concatenated gene trees were generated by aligning each gene in MEGA using MUSCLE (Edgar, 2004). Then, the gene alignments were trimmed using phyutility (−clean 0.95) and concatenated (−concat) (Smith and Dunn, 2008). Maximum likelihood phylogenetic trees with 500 bootstrap replications were made from the concatenated gene alignments using the Poisson model with default parameters. The phylogenetic trees were visualized in ITOL (Letunic and Bork, 2016). CompareM was used to determine the mean amino acid identity between related microbial genomes (see footnote 1).

### Geochemical Data Statistics

Geochemical data from the soil cores used for metagenome sequencing were analyzed using a MANOVA test in R version 3.2.4 (R Core Team, 2016).

### Data Availability

Contigs for all MAGs are available on figshare^3^ and NCBI BioProject PRJNA432584. The raw sequence FASTA files for the ten metagenomes are available at JGI (Supplementary Table S1).

## Results

### Metagenomes of Rainforest and Cattle Pasture Soil Exhibit Distinct Taxonomic and Functional Profiles

**FIGURE 1:**
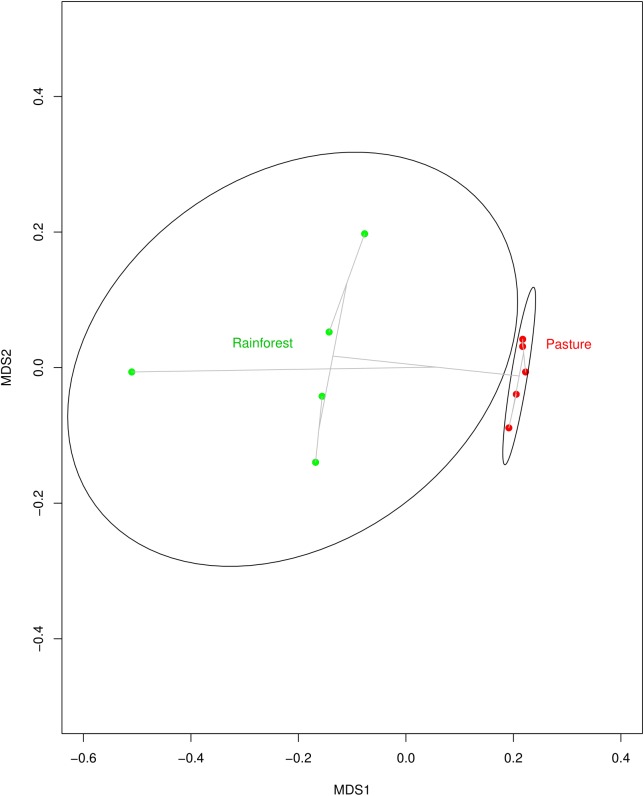
Multidimensional scaling (MDS) plot indicating how similar soil microbial communities from Rainforest samples (green dots) cluster together, but away from communities of the Pasture samples (red dots). The clustering is visualized with gray lines connecting samples with similar communities. The MDS plot was created using Bray–Curtis dissimilarity among samples and an average-linkage algorithm for community clustering.

With land-use change from rainforest to pasture there was a significant change in soil geochemistry with increases in acidic potential (total soil acidity), and in organic matter, copper, iron, and zinc concentrations in the cattle pasture, while boron and aluminum concentrations were significantly higher in rainforest soil (Supplementary Table S3). The dominant phyla in both the rainforest and cattle pasture soil metagenomes were Proteobacteria, Actinobacteria, Firmicutes, Acidobacteria, and Verrucomicrobia. Beyond this similarity, the microbial community composition changes drastically (**Figure 1**) with the abundance of 13 out of 34 identified microbial phyla significantly changing between rainforest and cattle pasture soils. Thaumarchaeota, a known ammonia-oxidizing archaeon, almost disappeared during rainforest-to-pasture conversion (loss of 99.5%). Other phyla significantly decreased by deforestation were Crenarchaeota, Nitrospirae, Gemmatimonadetes, Fusobacteria, Aquificae, Lentisphaerae, and Korarchaeota (Supplementary Figure S1 and Supplementary Table S4).

**FIGURE 2:**
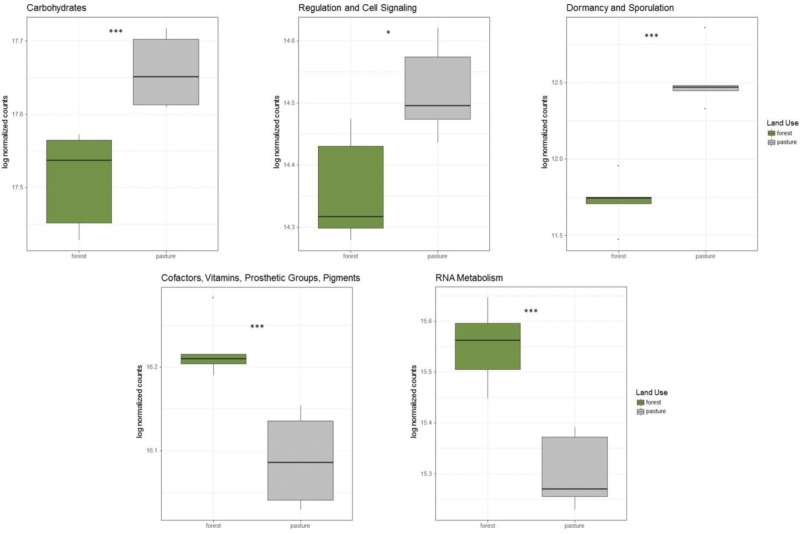
Significantly altered abundancies of functional gene groups between rainforest and pasture soils. These log-normalized counts were determined using DESeq2 (*p* < 0.05^∗^, *p* < 0.01^∗∗^, *p* < 0.001^∗∗∗^).

In addition to taxonomic shifts in the microbial community, there were significant alterations to functional groups between rainforest and pasture soil. Genes related to carbohydrate metabolism, dormancy and sporulation, and regulation and cell signaling were all significantly increased in the pasture compared to rainforest, while genes related to transcription and vitamin production were significantly higher in rainforest soil (**Figure 2**). Additionally, significant shifts were found in carbon cycling genes. Lignin degradation genes including a superoxide dismutase were significantly higher in rainforest soils. All methyl coenzyme M reductase genes that are essential for methanogenesis were significantly higher in pasture while the particulate methane monooxygenase genes important to methane oxidation were significantly higher in rainforest. Additionally, key genes involved carbon assimilation by methanotrophs using the serine-glyoxylate cycle were significantly higher in rainforest and the ribulose monophosphate pathway genes were significantly higher in pasture soils. Overall, there was a predominance of significantly higher genes involved in monosaccharide, oligosaccharide, and polysaccharide metabolism, along with fermentation in pasture (Supplementary Table S5).

### Genome-Resolved Metagenomics Exposed Novel Genomes From the Soil Rare Biosphere

**FIGURE 3:**
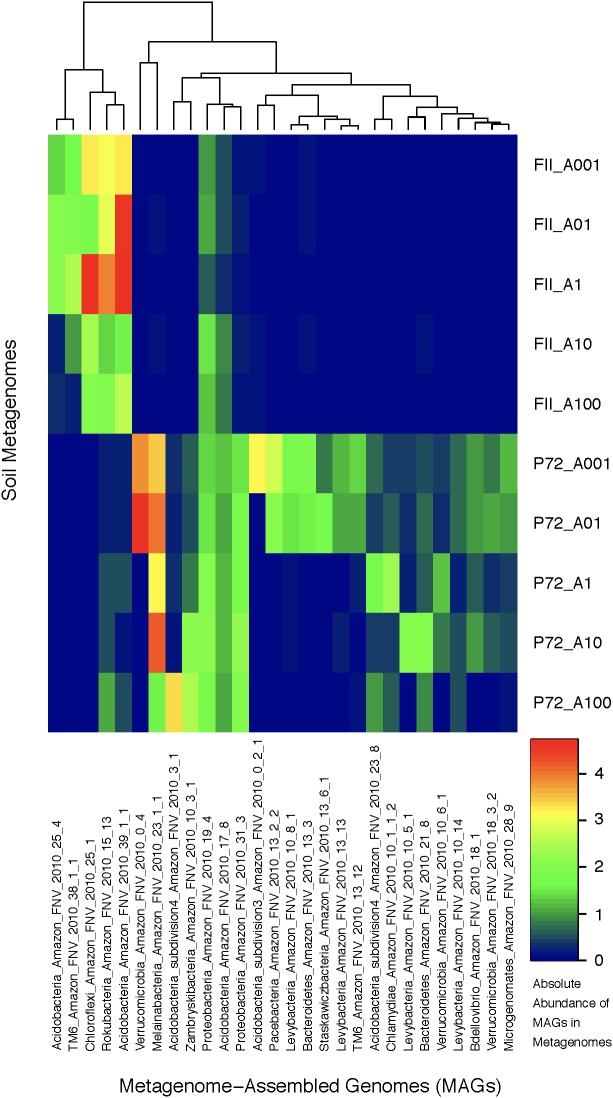
A heatmap of the absolute abundance of MAGs in each metagenome sample from this study. Absolute abundance means the number of the specific genome found in each soil metagenome; an absolute abundance of four would mean that four instances of that genome are found in that specific metagenome. The *x*-axis depicts the MAGs labeled with the following scheme: Taxonomic association, location of sampling (Amazon FNV), year of sampling (2010), and bin number from anvi’o. The *y*-axis are the soil metagenome samples for rainforest (FII) or cattle pasture (P72), differentiated by the location on a 100 m transect indicating the distance from the starting location in meters. The clustering at the top of the graph is based on hierarchical clustering.

Using contigs longer than 5 kb from each metagenome, we characterized 28 MAGs representing three of the dominant phyla (Proteobacteria, Acidobacteria, Verrucomicrobia) as well as other lineages (TM6, Bacteroidetes, Melainabacteria, Chloroflexi, Chlamydiae, Microgenomates, Parcubacteria, Rokubacteria) identified as members of the rare biosphere (<0.01%) based on iTag sequence annotations (Supplementary Table S6). The genome length ranged from 0.96 to 7.44 Mb with an average of 2.5 Mb due to many streamlined genomes from candidate phyla MAGs. The GC content ranged from 30.77 to 67.72% covering a large portion of known bacterial GC content (**Table 1** and Supplementary Table S2). If we consider genomes that are shorter than 2 Mb to be streamlined, then the average GC content is 38.12% with a range of 30.77–43.41%. Genomes longer than 2 Mb have an average GC content of 54.26% with a range of 42.76–67.72%. Although there are not many replicates at the MAG level, we observed a clear effect of land-use change on the abundance of specific taxonomic groups. MAGs found only in the pasture soils belong to the Microgenomates, Parcubacteria, Verrucomicrobia, Chlamydiae, and Melainabacteria, while MAGs of Rokubacteria and Chloroflexi were in much higher abundance in rainforest soils. Acidobacteria MAGs were found in both land-use types, with subdivisions 3 and 4 only found in pasture sites (**Figure 3**). Beyond the new insights we gained regarding the effects of land usage on the community composition of Amazon soils, the MAGs we have characterized correspond to many understudied bacterial lineages that deserve detailed descriptions of their unique functional features in the context of relevant genomic databases.

**Table 1:** Genome characteristics for the 11 metagenome-assembled genomes (MAGs) discussed thoroughly in this study.

Genome	Completion (%)	Redundancy (%)	Contigs	Length (Mb)	GC (%)	CDS	N50	Mean AAI	Top Hit	Taxonomy
Microgenomates Amazon FNV 2010 10_8_1	93.02	1.72	25	1.20	30.77	1240	57316	59.26	Microgenomates Amazon FNV 2010 10_14	Candidatus Levybacteria
Microgenomates Amazon FNV 2010 13_2_2	90.7	2.13	32	1.20	41.45	1272	84287	62.55	Microgenomates Amazon FNV 2010 13_13	Candidatus Pacebacteria
Parcubacteria Amazon FNV 2010 10_3_1	90.7	1.36	16	0.651	36.82	735	64234	67.57	Zambryskibacteria RIFCSPLOWO2 12 FULL 39_45	Candidatus Zambryskibacteria
Microgenomates Amazon FNV 2010 28_9	83.72	1.27	65	1.056	43.41	1258	19835	51.93	Microgenomates Amazon FNV 2010 13_13	^∗∗^Candidatus Cerribacteria
Parcubacteria Amazon FNV 2010 13_6_1	81.4	0	43	0.534	35.07	631	14301	68.37	Staskawiczbacteria RIFOXYD2 FULL 37_9	Candidatus Staskawiczbacteria
Microgenomates Amazon FNV 2010_13_13	76.74	0.72	85	0.813	42.24	928	9549	62.55	Microgenomates_Amazon FNV 2010 13_2_2	Candidatus Pacebacteria
Acidobacteria Amazon FNV 2010 0_2_1	68.03	5.93	405	7.44	60.03	6869	24087	66.20	Solibacter usitatus	Acidobacteria subdivision 3
Melainabacteria Amazon FNV 2010 23_1_1	63.73	1.16	294	3.04	50.61	2991	11292	56.14	Obscuribacter phosphatis	Melainabacteria
Microgenomates Amazon FNV 2010 10_5_1	53.49	0.54	107	1.37	36.42	1471	13537	59.26	Microgenomates Amazon FNV 2010 10_14	Candidatus Levybacteria
Rokubacteria Amazon FNV 2010 15_13	44.71	3.65	462	3.35	67.72	3636	10946	61.09	Rokubacteria GWA2 70 _23	Candidatus Rokubacteria
Microgenomates Amazn FNV 2010 10_14	39.53	0.62	92	0.964	38.16	1059	10365	59.26	Microgenomates Amazon FNV 2010 10_5_1	Candidatus Levybacteria

*CDS, number of conserved domain sequences; Mean AAI, mean amino acid identity for the best hit using compare, and the taxonomy of the MAG as determined using mean AAI and concatenated ribosomal proteins. ^∗∗^Proposed novel phylum within candidate phyla radiation.*

### Amazon Acidobacteria Involved in Pasture Hydrocarbon Metabolism

The six Acidobacteria MAGs represented five of the currently 26 described subdivisions (Supplementary Figure S2). Amongst these are MAGs from three of the most well characterized subdivisions 1, 3, and 4, and from the lesser known subdivision 13, which has no cultured representatives. Many Acidobacteria subdivisions do not currently have cultured representatives; therefore, molecular marker genes are used to determine the different subdivisions. For Acidobacteria the 16S rRNA gene is used, which were not detected in many of the Acidobacteria MAGs from this study. Unfortunately, concatenated ribosomal proteins are not able to provide clarity, which leaves some of the Acidobacteria MAGs from this study in undetermined subdivisions. Only Acidobacteria with confirmed subdivisions were analyzed for unique potential functions. An interesting feature of the Amazon Acidobacteria is that five out of six have a 4-hydroxybenzoate transporter found in only nine other Acidobacteria MAGs (out of 186 other MAGs publicly available on NCBI analyzed). The Amazon Acidobacteria MAG from subdivision 3 (Bin_0_2_1; 68% completion; 5.93% redundancy) exhibited the highest completion and was further characterized. This MAG has the functional potential to metabolize many carbon substrates including aromatic compounds, starch, glycogen, cellobiose, glucose, fructose, sucrose, maltose, and trehalose. Furthermore, this Amazon Acidobacterium has genes involved in nitrogen and sulfur cycling with the potential to reduce nitrite, nitric-oxide, sulfate, and sulfite.

### Melainabacteria Involved in Pasture Carbon Cycling

One Melainabacteria MAG corresponded to a newly discovered lineage within the candidate order Obscuribacter (Supplementary Figure S3 and **Table 1**). This MAG contains 72 unique FIGFams (i.e., protein encoding genes) when compared to the 55 Melainabacteria available in NCBI. Some features of interest were a nitric-oxide reductase (quinol-dependent) and a ubiquinol-cytochrome c reductase only found in *Candidatus Obscuribacter phosphatis* and the Amazon Melainabacteria. Similar to *O. phosphatis*, the Amazon Melainabacteria has the genetic potential to metabolize simple carbon sources including fructose, glucose, starch, glycogen, and maltose. However, only the Amazon Melainabacteria MAG contains genes to synthesize cellulose from UDP-alpha-D-glucose and can potentially metabolize cellobiose, maltose, and xylose. This Amazon Melainabacteria MAG also contains genes for both aerobic and anaerobic respiration including a complete respiratory chain like *O. phosphatis*.

### Amazon Rokubacteria Potentially Oxidize Methane in Rainforest Soil

**FIGURE 4:**
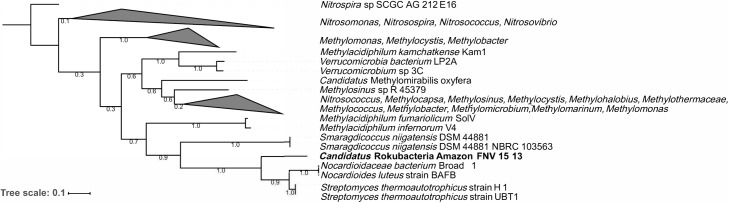
Phylogenetic tree of the particulate methane monooxygenase alpha subunit (*pmoA*). The *pmoA* sequences identified with PATRIC were used to create a maximum likelihood tree with 500 bootstrap replications. The *pmoA* from this study’s Amazon Rokubacteria MAG is indicated in bold. The numbers on each node represent the bootstrap support.

The Amazon Rokubacteria MAG (Amazon-R-15-13) contains 103 unique FIGFams when compared to the 75 available Rokubacteria MAGs from NCBI and the recently published single cell genomes from Becraft et al. (2017) (Supplementary Table S7). Among the features unique to Amazon-R-15-13 is a complete particulate methane monooxygenase (pmmo) necessary for methane oxidation to methanol. A phylogenetic analysis of the pmmo alpha subunit (*pmoA*) found the Amazon-R-15-13 *pmoA* to be unique and most closely related to *pmoA* genes from *Streptomyces thermoautotrophicus*, *Nocardioides luteus*, and *Smaragdicoccus niigatensis* (**Figure 4**). The lateral gene transfer (LGT) analysis found that LGT-predicted genes are spread across many contigs supporting proper binning and none of the annotated genes were related to methane cycling (Supplementary Table S8).

A meta-analysis of all 52 Rokubacteria MAGs available in NCBI and of our Amazon Rokubacteria MAG revealed several genomic features supporting the metabolic potential to metabolize larger hydrocarbons. The majority of soil Rokubacteria MAGs (17 out of 22) have P450 enzymes necessary for the oxidation of large hydrocarbons. Rokubacteria MAGs (16 out of 53) contain genes for alkane 1-monooxygenase enzymes to oxidize shorter hydrocarbons. Following hydrocarbon metabolism, Rokubacteria MAGs have the potential for beta-oxidation of fatty acids. These functional features suggest that soil Rokubacteria have the potential to metabolize hydrocarbons followed by β-oxidation of fatty acids, which then feeds into the TCA cycle and the electron transport chain (Supplementary Table S9).

**FIGURE 5:**
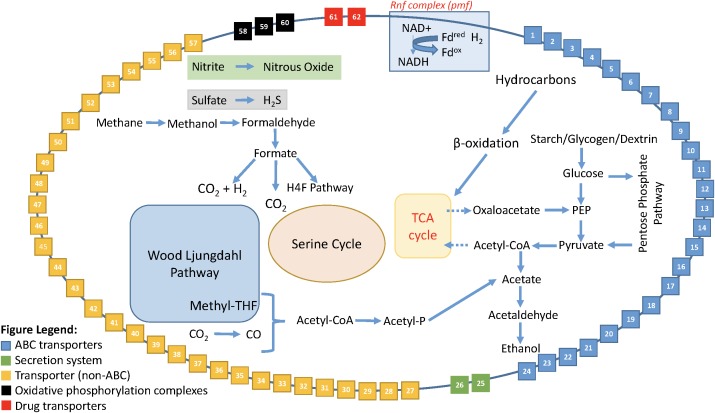
Model of the metabolic reconstruction of *Candidatus* Rokubacteria based on PATRIC annotations. Indicated are the major metabolic processes including central carbon metabolism and energy generation in the center of the cell. The cell wall includes the various transporters, secretion systems, and electron transport complexes labeled by number. All red asterisks indicate that a particular function is found only in the Amazon Rokubacteria MAG when compared to the other 52 MAGs available for Rokubacteria. The legend for the cell wall box numbers is found in Supplementary Table S10.

Enabled by the expanded dataset of 53 Rokubacteria MAGs, a conceptual metabolic model was formed based on annotated genes (**Figure 5** and Supplementary Table S10). This model is based on gene annotations without gap-filling and extends our knowledge of phylum *Candidatus* Rokubacteria. Rokubacteria MAGs have many metabolic capabilities including hydrocarbon metabolism, but the hydrocarbon methane can follow a different fate. Methane can be oxidized through three enzymatic steps to formate, which can then follow several paths: (1) it can be converted to acetyl-CoA using the tetrahydrofolate (H4F) pathway, (2) it can be oxidized to carbon dioxide via formate dehydrogenase, or (3) it can be oxidized to carbon dioxide and hydrogen using a formate hydrogen lyase (**Figure 5**). When methane is not converted to acetyl-CoA via the H4F pathway, Rokubacteria MAGs have the functional potential to utilize the serine cycle for carbon assimilation (Supplementary Table S9). Rokubacteria MAGs have the genomic potential to produce acetate and ethanol via the Wood–Ljungdahl pathway. There are genes for an electron transport Rnf complex in 20 Rokubacteria MAGs that is involved in energy generation. This phylum has the genomic potential to use all three central metabolic pathways and although there are signs of aerobic respiration, such as cytochrome c oxidase, there are also indications of anaerobic respiration including the potential to denitrify nitrate to nitrous oxide (Supplementary Table S9). Another possible electron acceptor is sulfate using dissimilatory sulfate reduction; although, there are also a few MAGs with assimilatory sulfate reduction genes (Supplementary Table S9). The wide variety of carbon substrates, energy sources, and electron acceptors may help this phylum survive in varying environmental conditions.

### Amazon Microgenomates Retain DNA Mismatch Repair Machinery

Our database contains six MAGs from the CPR superphylum Microgenomates, which correspond to the first soil genomic representatives of this large branch of the tree of life. Two of these MAGs (Amazon-Pacebacteria-13-2-2 and Amazon-Levybacteria-10-8-1) are over 90% complete (**Table 1**). Phylogenetic analyses uncovered that the three genomes Amazon-Levybacteria-10-8-1, Amazon-Levybacteria-10-5-1, and Amazon-Levybacteria-10-14 are most closely related to each other, and fall within *Candidatus* Levybacteria. MAGs Amazon-Pacebacteria-13-2-2, Amazon-Pacebacteria-13-13, and Amazon-Microgenomates-28-9 are most closely related to each other with Amazon-Pacebacteria-13-2-2 and Amazon-Pacebacteria-13-13 forming a basal monophyletic clade with *Candidatus* Pacebacteria. Amazon-Microgenomates-28-9 likely represents a novel candidate phylum within the superphylum Microgenomates based on its low mean amino acid identity compared to known phyla and its phylogenetic placement between Pacebacteria and Chisholmbacteria. Thus, we propose the name *Candidatus* Cerribacteria (**Table 1** and Supplementary Figure S4).

An extensive analysis across all Microgenomates MAGs (*n* = 859) available on NCBI revealed only 13 genomes with the essential DNA mismatch repair machinery (*mutS, mutL*). In contrast, we found *mutS, mutL*, and *uvrD* in all but one of our Amazon Microgenomates MAGs (Supplementary Table S11). Although almost none of the Microgenomates MAGs (13 out of 859) analyzed have *mutS/L*, *uvrD* is found in all the phyla within the superphylum Microgenomates. Genes involved in DNA replication initiation (*DnaA*, *DnaB*) are still present, which supports that these organisms can possibly still replicate independently from a host.

### Amazon Pacebacteria Have Expanded Carbohydrate Metabolism

An additional three genomes reconstructed from Amazon soil are most closely related to *Candidatus* Pacebacteria, one of which is thought to represent a novel phylum for which we propose the name *Candidatus* Cerribacteria. A pangenomic analysis of the four available Pacebacteria MAGs together with the three novel Amazon MAGs revealed a “core” of gene clusters. This “core” encodes for proteins involved in mannose metabolism, one-carbon pool by folate, glycolysis, and an ATP synthase (Supplementary Figure S5). This “core” provides a base understanding of their metabolism, which can utilize basic sugars like glucose and mannose via glycolysis and the pentose phosphate pathway to produce lactic acid. This phylum completely lacks the TCA cycle and is unable to produce acetyl-CoA or oxaloacetate to feed into the cycle. All genomes contain genes to use the one carbon pool by folate pathway, but none of them contain genes to perform folate biosynthesis except Amazon-Pacebacteria-13-2-2. The three Amazon Microgenomates MAGs most closely related to Pacebacteria formed a grouping of their own with 180 gene calls found in at least two genomes including genes for proteins essential for starch/glycogen metabolism. Additionally, Amazon-Pacebacteria-13-2-2 encoded a cellulase protein necessary for cellulose metabolism.

### Amazon Parcubacteria Are Phylogenetically Distinct, but Functionally Redundant

Two Amazon MAGs fall within the superphylum Parcubacteria and are the first representative genomes of this lineage in soil (Supplementary Figure S6 and **Table 1**). Most of the unique genes from these MAGs encode hypothetical proteins. Nevertheless, some functional annotations unique to Amazon Parcubacteria MAGs (compared to 236 genomes available on NCBI) are a hyaluronan synthase and cadherin-like proteins (RTX toxins). Amazon Parcubacteria have reduced metabolic capabilities, but potentially can break down various simple sugars to pyruvate using glycolysis and the pentose phosphate shunt, which is then converted to D-lactate. Amazon-Parcubacteria-13-6-1 is also capable of synthesizing trehalose from glucose-1-phosphate. Overall, the functional potential of soil Parcubacteria MAGs are in accordance with other studies, but Amazon Parcubacteria are phylogenetically distinct (Supplementary Figure S6).

## Discussion

In this study, we investigated the impact of deforestation on the genomic diversity and functional potential of soil microorganisms using metagenomics and MAGs. Previous studies have examined how forest-to-pasture conversion in the Brazilian Amazon affects soil microbial communities (Jesus et al., 2009; Rodrigues et al., 2013; Navarrete et al., 2015; Meyer et al., 2017) and discovered that land-use change is accompanied by significant shifts in the geochemistry and the microbial community of the soil. Jesus et al. (2009) determined that pasture soil microbial communities were significantly different from rainforest soil communities driven by pH, copper, and iron concentrations. Our study concurs with Jesus et al. (2009) that as the soil geochemistry changes the microbial community shifts as well with pH, iron, copper, and zinc significantly increasing in pasture soil. Previous work by Meyer et al. (2017) identified key shifts in methane cycling taxa and life history traits between rainforest and pasture samples. This study expands on work from Meyer et al. (2017) by focusing more broadly on genes related to the degradation of carbohydrates. On average, we have observed higher soil C content (g kg^−1^ soil) in the pasture sites (2.93 ± 0.65; mean ± 95% CI) than in the forest sites (1.52 ± 0.42), which suggests that higher soil C content might have selectively favored copiotrophic microorganisms with diverse carbohydrate metabolism. Furthermore, the two fast growing grass species grown in the pastures have been reported to secrete high quantities of labile C (Reis et al., 2001). Lignin is a highly aromatic, recalcitrant carbon compound produced by plants (Novaes et al., 2010). Previous work by Lammel et al. (2015) found a significantly higher abundance of lignin in rainforest compared to pasture soils, while the pasture samples had significantly higher amounts of hemicellulose. Using metagenomics, we found a differential abundance of genes related to lignin degradation in the rainforest and an increase in genes related to hemicellulose and cellulose degradation in the pasture soil. This is not surprising due to the drastic change in plant species from a diverse rainforest of large tropical trees with lignin contents around 40% to a monoculture of *Urochloa brizantha* (and a few occurrences of *Panicum maximum*) with ∼5–10% lignin (Herrero et al., 2001; Novaes et al., 2010; Mauri et al., 2015). As the carbon sources available in each land-use type change, we see significant alterations to the microbial community composition with a higher abundance of Firmicutes in the pasture. This is not unexpected since previous research has found that Firmicutes respond to increased organic matter (Cleveland et al., 2006). We also observed an almost complete loss of the ammonia-oxidizing archaea Thaumarchaeota in the pasture soil, confirming earlier studies (Hamaoui et al., 2016). A study by Subbarao et al. (2009) identified that the grass used in Brazilian cattle pastures (*Urochloa*) produces biological nitrification inhibitors (BNIs), specifically a cyclic diterpene termed brachialactone, that inhibits both the ammonia monooxygenase (amo) and hydroxylamine oxidoreductase (hao). These *Urochloa* grasses that produce BNIs have been shown to inhibit both ammonia-oxidizing bacteria and archaea (Moreta et al., 2014).

By assembling MAGs, we have gained a new depth of insight into the putative life strategies of some Amazonian soil microorganisms that is not possible with only metagenomics. In pastures we see a change in carbon metabolism coherent with results from our metagenomic data. These observed increases in fermentation and diversified carbon sources may have long-term consequences on carbon storage and greenhouse gas emissions. The Acidobacteria MAG from subdivision 3 (Bin_0_2_1) have the genetic potential to degrade a variety of carbon substrates similar to many related Acidobacteria (Kielak et al., 2016) but has the unique genetic setup to degrade aromatic compounds. The abundance of Acidobacteria subdivisions have been previously found to significantly change between land-use types (Navarrete et al., 2013, 2015) with subdivisions 4, 7, 10, and 17 increasing in pasture, and subdivisions 2 and 13 increasing in rainforest soils. Due to Acidobacteria’s predominance in soils, it has been previously proposed this group may be involved in the degradation of aromatic compounds in soil and with continued efforts to sequence and isolate these organisms we will have a better understanding of their role in soil carbon cycling (Pérez-Pantoja et al., 2010). Melainabacteria (Bin_23_1_1) appears to encode the ability to breakdown a variety of carbon substrates as well, including cellobiose. This group of non-photosynthetic fermenters have been found to metabolize cellulose and cellobiose in soil using stable isotope probing experiments (Soo et al., 2014; Pepe-Ranney et al., 2016; Juottonen et al., 2017). Both Amazon Acidobacteria subdivision 3 and Melainabacteria were found only in the pasture soil metagenomes likely playing an important role in metabolizing polycyclic aromatic hydrocarbons and plant-derived carbon deposited from the slash and burn deforestation practice. While we do not have the MAG replication to definitely test whether Acidobacteria subdivisions 3 and 4 and Melainabacteria are changing in abundance, we only detect their presence in one of the land uses, suggesting they are affected by land use change. Since Acidobacteria are considered ubiquitous in soils it is important to understand how the different subdivisions may be affected by land-use change. All Microgenomates and Parcubacteria MAGs were found only in the pasture, suggesting these groups are also influenced by land use change. This study described the first Microgenomates MAGs from soil with one MAG representing a novel phylum for which we proposed the name *Candidatus* Cerribacteria. Although the potential physiology of these candidate phyla MAGs was similar to what has previously been described (Wrighton et al., 2012; Rinke et al., 2013; Brown et al., 2015), the Amazon Microgenomates MAGs retained essential DNA mismatch repair genes (*mutS*, *mutL*) that are highly conserved in bacteria likely due to their role in providing genomic stability (Fukui, 2010). With 859 genomes examined, there is strong evidence that selection has or is occurring either for the loss of DNA mismatch repair in subsurface Microgenomates or for our Amazon soil Microgenomates to retain such machinery. These mismatch repair proteins provide DNA stability under the stress of environmental fluctuations, which in the case of cattle pasture may be temperature, moisture, and UV radiation (Denamur et al., 2000). In pathogenic or endosymbiotic bacteria, the loss of DNA repair and recombination proteins increases mutation rates which is thought to cause AT bias in small genomes (Moran and Wernegreen, 2000).

In rainforest soil we found a differential abundance of genes related to methanotrophy as has been previously reported by Meyer et al. (2017). The Rokubacteria MAG (Bin_15_13) was found predominantly in the rainforest soil metagenomes, which is not surprising due to the rainforests wide array of potential carbon sources, and in particular its ability to oxidize more methane than it produces. Rokubacteria have been described to oxidize methanol (Butterfield et al., 2016), but this is the first study to describe this group’s potential role in methane oxidation. This discovery led to an investigation into potential hydrocarbon metabolism that appears to be wide-spread in the soil MAGs. An electron transport Rnf complex was found in many of the Rokubacteria MAGs, which is suggested to be essential for ATP synthesis in some acetogens when grown autotrophically on H_2_ and CO_2_ by creating a proton motive force (Biegel et al., 2011; Tremblay et al., 2012). Our expanded analyses of the Rokubacteria MAGs indicate a diverse metabolism within this phylum including a wide variety of carbon sources ranging from gaseous substrates to complex hydrocarbons, many possible energy sources, and electron acceptors.

The combination of metagenome and MAG analyses allowed for increased understanding of the potential biological functions altered by land-use change. Carbon cycling in both datasets appeared to be significantly altered by deforestation, especially related to complex carbon sources like lignin and polycyclic aromatic hydrocarbons. This study provides the basis for continuing research into how deforestation affects soil microbial communities, biogeochemical cycling, and for the first-time tropical soil candidate phyla.

## Author Contributions

MK contributed all metagenome and MAG analyses, all statistical analyses, drafted the manuscript, and drafted the figures and tables. JR, BB, CB, JT, SMT, and KN contributed sampling collection. JG contributed metagenome co-assembly and critical review of manuscript. KM provided raw sequence annotations from MG-RAST and critical review of manuscript. ST and KK contributed in revising of manuscript. TD, AE, and KN contributed in writing and revising of manuscript.

## Conflict of Interest Statement

The authors declare that the research was conducted in the absence of any commercial or financial relationships that could be construed as a potential conflict of interest.

## Footnotes

1 https://github.com/dparks1134/CompareM

2 http://merenlab.org/2016/11/08/pangenomics-v2/

3 https://doi.org/10.6084/m9.figshare.5151571.v1
