# Supplementary material for: New Biological Insights Into How Deforestation in Amazonia Affects Soil Microbial Communities Using Metagenomics and Metagenome-Assembled Genomes
Source: Front Microbiol. 2018 Jul 23;9:1635. doi: 10.3389/fmicb.2018.01635 (PMC6064768; doi:10.3389/fmicb.2018.01635)
Supplement: Supplementary file 1 [file Image_1.PDF]

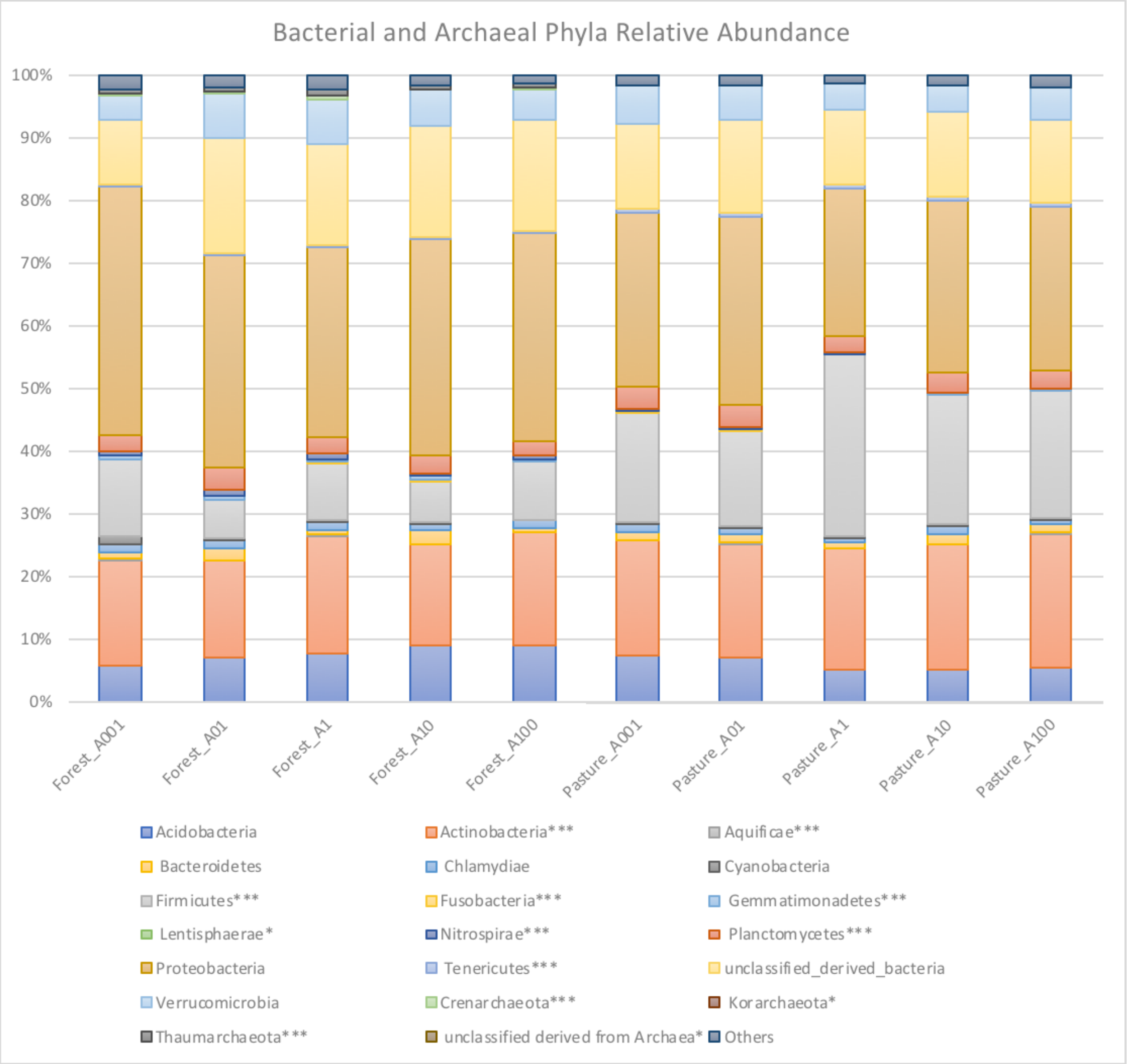

FIGURE S1 The relative abundance of bacterial and archaeal phyla in Amazon rainforest (Forest) and adjacent cattle pasture (Pasture) soil. The numbers after "A" in the sample names indicate the distance to the start location on a 100 m transect. The asterisks next to phyla names indicate the significance ($p<0.05$) determined by DESeq2 ($p<0.05$*, $p<0.01$**, $p<0.001$***).
