# Supplementary material for: New Biological Insights Into How Deforestation in Amazonia Affects Soil Microbial Communities Using Metagenomics and Metagenome-Assembled Genomes
Source: Front Microbiol. 2018 Jul 23;9:1635. doi: 10.3389/fmicb.2018.01635 (PMC6064768; doi:10.3389/fmicb.2018.01635)
Supplement: Supplementary file 2 [file Image_2.pdf]

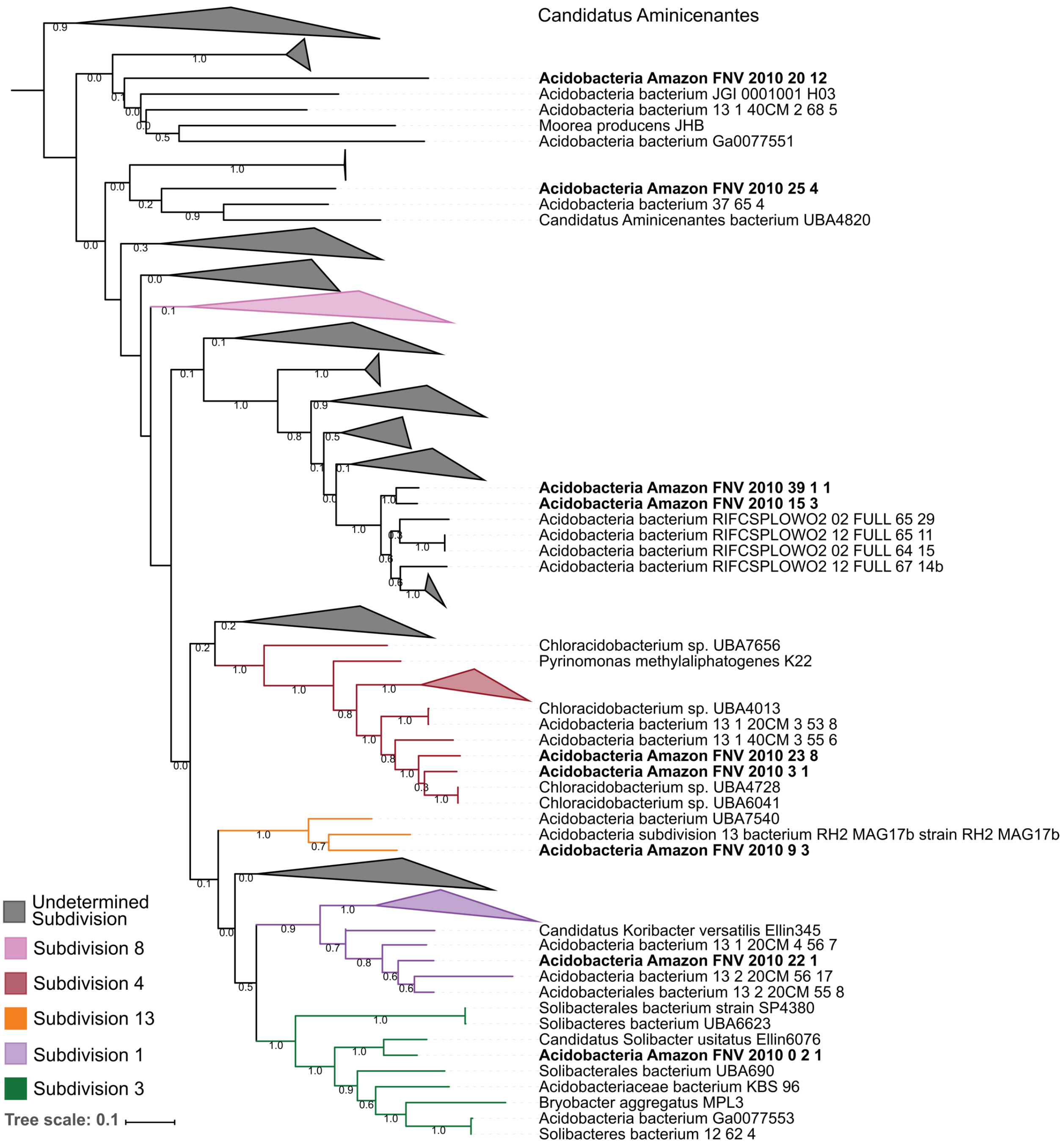

FIGURE S2 Maximum likelihood phylogenetic tree of 16 concatenated ribosomal proteins from Acidobacteria with Candidatus Aminicenantes as the outgroup, with 500 bootstrap replicates. The genomes identified in this study are indicated in bold. The numbers on each node represent the bootstrap support. Colored monophyletic clades indicate a specific Acidobacteria subdivision as determined by the inclusion of a known member. Green = subdivision 3, Purple = subdivision 1, Orange = subdivision 13, Maroon = subdivision 4, Pink = subdivision 8. All grey clades are undetermined subdivisions.
