# Supplementary material for: New Biological Insights Into How Deforestation in Amazonia Affects Soil Microbial Communities Using Metagenomics and Metagenome-Assembled Genomes
Source: Front Microbiol. 2018 Jul 23;9:1635. doi: 10.3389/fmicb.2018.01635 (PMC6064768; doi:10.3389/fmicb.2018.01635)
Supplement: Supplementary file 3 [file Image_3.pdf]

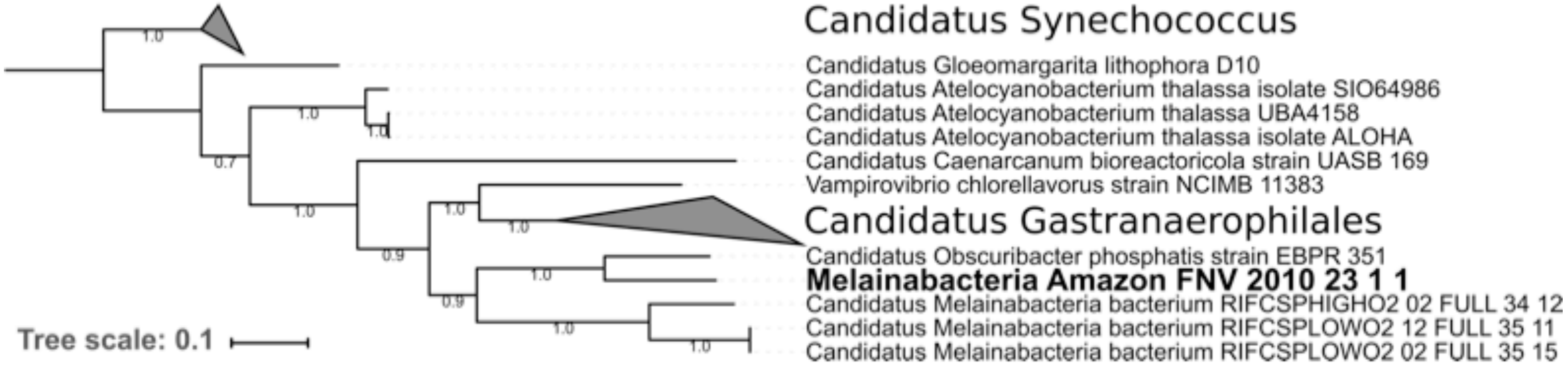

FIGURE S3 Maximum likelihood phylogenetic tree of Melainabacteria using 16 concatenated proteins and Candidatus Synechococcus as the outgroup, with 500 bootstrap replicates. The MAG from this study is indicated in bold. The numbers on each node represent the bootstrap support.
