# Supplementary material for: New Biological Insights Into How Deforestation in Amazonia Affects Soil Microbial Communities Using Metagenomics and Metagenome-Assembled Genomes
Source: Front Microbiol. 2018 Jul 23;9:1635. doi: 10.3389/fmicb.2018.01635 (PMC6064768; doi:10.3389/fmicb.2018.01635)
Supplement: Supplementary file 4 [file Image_4.PDF]

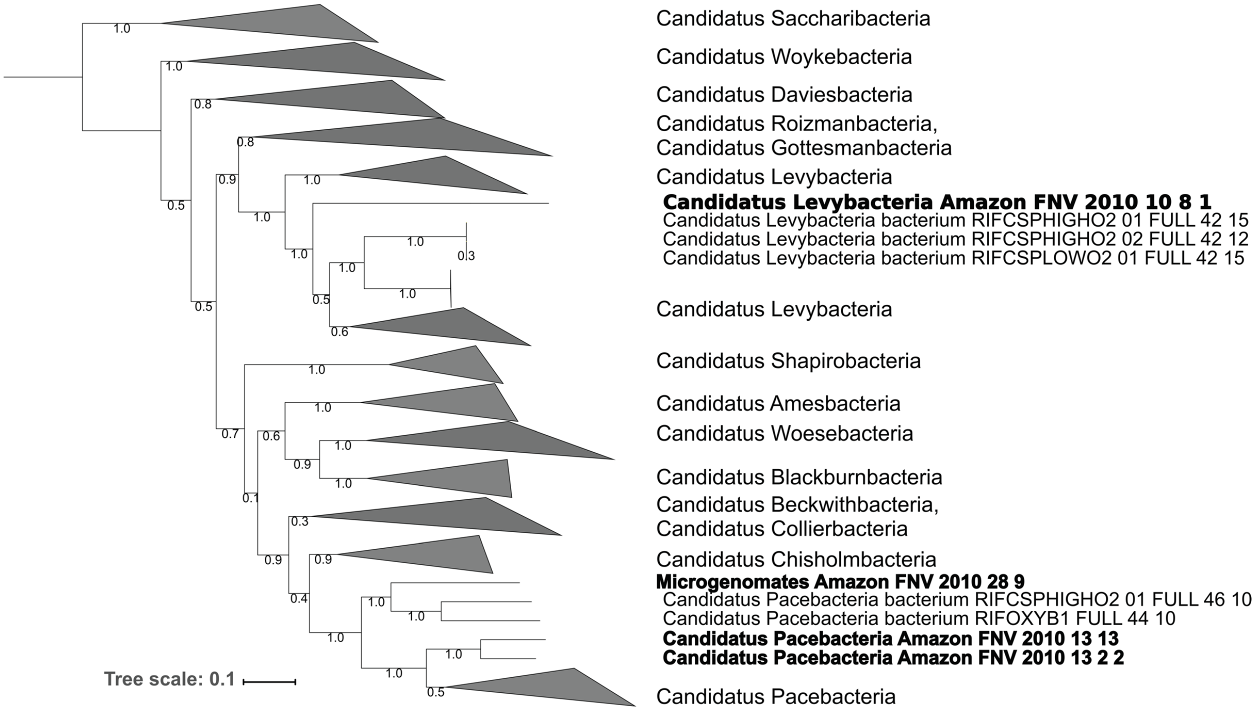

FIGURE S4 Maximum likelihood phylogenetic tree of 16 concatenated ribosomal proteins from superphylum Microgenomates with Candidatus Saccharibacteria (TM7) as the outgroup, with 500 bootstrap replicates. The genomes from this study are indicated in bold. The numbers on each node represent the bootstrap support.
