# Supplementary material for: New Biological Insights Into How Deforestation in Amazonia Affects Soil Microbial Communities Using Metagenomics and Metagenome-Assembled Genomes
Source: Front Microbiol. 2018 Jul 23;9:1635. doi: 10.3389/fmicb.2018.01635 (PMC6064768; doi:10.3389/fmicb.2018.01635)
Supplement: Supplementary file 5 [file Image_5.PDF]

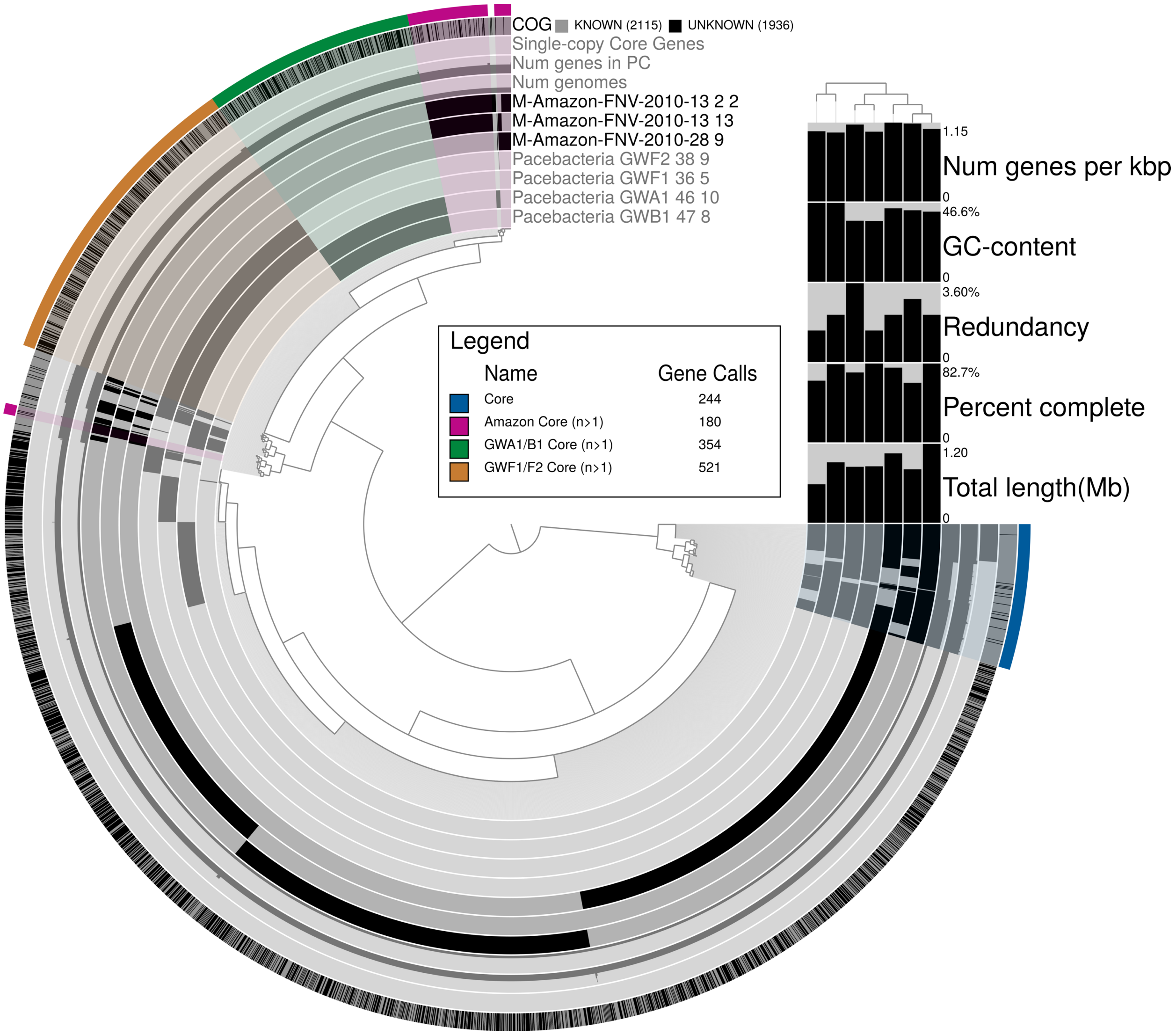

FIGURE S5 A pangenome analysis of *Candidatus* Pacebacteria and the three Amazon MAGs most closely related to *Candidatus* Pacebacteria. The "core" proteins found in the 7 genomes analyzed are highlighted in blue. The "core" of the Amazon pangenome is highlighted in pink. The "core" pangenome for *Candidatus* Pacebacteria genomes GWA1 and GWB1 are highlighted in green and the "core" for *Candidatus* Pacebacteria genomes GWF1 and GWF2 are highlighted in orange. The number of genes per kilobase pair, GC content, percent redundancy, percent completion, and total length of each genome are visualized in the bar chart. PC = protein cluster.
