# Supplementary material for: New Biological Insights Into How Deforestation in Amazonia Affects Soil Microbial Communities Using Metagenomics and Metagenome-Assembled Genomes
Source: Front Microbiol. 2018 Jul 23;9:1635. doi: 10.3389/fmicb.2018.01635 (PMC6064768; doi:10.3389/fmicb.2018.01635)
Supplement: Supplementary file 7 [file Table_1.PDF]

Supplemental Table 1: Overall alignment of each metagenome (MG) sample to the metagenome co-assembly. The alignment and number of reads in each sample only includes contigs longer than 5000 bp. Both, the IMG data set and the raw sequences are available for the metagenomes under the JGI project IDs.

Sample	Total reads in sample	overall alignment to MG-coassembly (%)	JGI Project ID
Forest A001	505512939	0.0085	1080879
Forest A1	137681004	0.0098	1080881
Forest A01	545382676	0.0084	1080880
Forest A10	588223706	0.0057	1080882
Forest A100	665802098	0.0085	1080883
Pasture A001	416868401	0.0249	1080884
Pasture A01	552101991	0.0231	1080885
Pasture A10	592561540	0.0184	1080886
Pasture A1	391519569	0.0157	1080888
Pasture A100	447516169	0.0151	1080887
