# Supplementary material for: New Biological Insights Into How Deforestation in Amazonia Affects Soil Microbial Communities Using Metagenomics and Metagenome-Assembled Genomes
Source: Front Microbiol. 2018 Jul 23;9:1635. doi: 10.3389/fmicb.2018.01635 (PMC6064768; doi:10.3389/fmicb.2018.01635)
Supplement: Supplementary file 8 [file Table_2.PDF]

Supplemental Table 2: Genome Reporting Standards and metadata for the MAGs of this study (as outlined by Bower *et al*. 2017)

Name	Microgenomates Amazon FNV 2010 10_8_1	Microgenomates Amazon FNV 2010 13_2_2	Parcubacteria Amazon FNV 2010 10_3_1	Microgenomates Amazon FNV 2010 28_9	Parcubacteria Amazon FNV 2010 13_6_1	Microgenomates Amazon FNV 2010_13_13	TM6 Amazon FNV 2010 13_12	TM6 Amazon FNV 2010 38_1_1
**Analysis Project Type**	MAG	MAG	MAG	MAG	MAG	MAG	MAG	MAG
**Taxa ID approach**	multi marker approach	multi marker approach	multi marker approach	multi marker approach	multi marker approach	multi marker approach	multi marker approach	multi marker approach
**Taxa ID**	Candidatus Levybacteria	Candidatus Pacebacteria	Candidatus Zambryskibacteria	Microgenomates unclassified	Candidatus Staskawiczbacteria	Candidatus Pacebacteria	TM6	TM6
**Assembly software**	anvi'o	anvi'o	anvi'o	anvi'o	anvi'o	anvi'o	anvi'o	anvi'o
**Annotation pipeline/tool**	PATRIC/RAST	PATRIC/RAST	PATRIC/RAST	PATRIC/RAST	PATRIC/RAST	PATRIC/RAST	PATRIC/RAST	PATRIC/RAST
**Quality**	high-quality	high-quality	high-quality	medium-quality	medium-quality	medium-quality	medium-quality	medium-quality
**Completion (%)**	93.02	90.7	90.7	83.72	81.4	76.74	60.95	57.09
**Contamination (%)**	1.72	2.13	1.36	1.27	0	0.72	4.15	0.98
**completeness software**	anvi'o	anvi'o	anvi'o	anvi'o	anvi'o	anvi'o	anvi'o	anvi'o
**number of contigs**	25	32	16	65	43	85	94	163
**16S recovered**	putative	putative	no	putative	putative	no	no	putative
**16S recovery software**	checkM	checkM	checkM	checkM	checkM	checkM	checkM	checkM
**total # tRNAs**	44	56	43	43	33	23	15	15
**# of standard tRNAs extracted**	19	18	19	19	18	15	10	9
**tRNA detection software**	tRNAscan-se	tRNAscan-se	tRNAscan-se	tRNAscan-se	tRNAscan-se	tRNAscan-se	tRNAscan-se	tRNAscan-se
**length (Mb)**	1.198	1.2	0.651	1.056	0.534	0.813	1.113	1.59
**GC content(%)**	30.77	41.45	36.82	43.41	35.07	42.24	41.06	39.11
**# CDS**	1240	1272	735	1258	631	928	1057	1607
**N50**	57316	84287	64234	19835	14301	9549	13537	10946
**completeness approach**	marker gene based	marker gene based	marker gene based	marker gene based	marker gene based	marker gene based	marker gene based	marker gene based
**decontamination software**	anvi'o	anvi'o	anvi'o	anvi'o	anvi'o	anvi'o	anvi'o	anvi'o
**bin parameters**	kmer+coverage+codon usage	kmer+coverage+codon usage	kmer+coverage+codon usage	kmer+coverage+codon usage	kmer+coverage+codon usage	kmer+coverage+codon usage	kmer+coverage+codon usage	kmer+coverage+codon usage
**binning software**	anvi'o	anvi'o	anvi'o	anvi'o	anvi'o	anvi'o	anvi'o	anvi'o
**reassembly post binning**	no	no	no	no	no	no	no	no
**mag coverage software**	anvi'o	anvi'o	anvi'o	anvi'o	anvi'o	anvi'o	anvi'o	anvi'o

Name	Microgenomates Amazon FNV 2010 10_5_1	Rokubacteria Amazon FNV 2010 15_13	Microgenomates Amazn FNV 2010 10_14	Chlamydiae_Amazon_FNV_2010_10_1_1_2	Bdellovibrio_Amazon_FNV_2010_18_1	Verrucomicrobia_Amazon_FNV_2010_0_4	Verrucomicrobia_Amazon_FNV_2010_18_3_2	Verrucomicrobia_Amazon_FNV_2010_10_6_1
**Analysis Project Type**	MAG	MAG	MAG	MAG	MAG	MAG	MAG	MAG
**Taxa ID approach**	multi marker approach	multi marker approach	multi marker approach	multi marker approach	multi marker approach	multi marker approach	multi marker approach	multi marker approach
**Taxa ID**	Candidatus Levybacteria	Candidatus Rokubacteria	Candidatus Levybacteria	Bacteria	Bacteria	Bacteria	Bacteria	Bacteria
**Assembly software**	anvi'o	anvi'o	anvi'o	anvi'o	anvi'o	anvi'o	anvi'o	anvi'o
**Annotation pipeline/tool**	PATRIC/RAST	PATRIC/RAST	PATRIC/RAST	PATRIC/RAST	PATRIC/RAST	PATRIC/RAST	PATRIC/RAST	PATRIC/RAST
**Quality**	medium-quality	low-quality	low-quality	high-quality	medium-quality	medium-quality	medium-quality	medium-quality
**Completion (%)**	53.49	44.71	39.53	89.11	78.87	78.58	73.48	71.78
**Contamination (%)**	0.54	3.65	0.62	1.95	2.41	3.19	1.03	0.74
**completeness software**	anvi'o	anvi'o	anvi'o	anvi'o	anvi'o	anvi'o	anvi'o	anvi'o
**number of contigs**	107	462	92	85	319	372	202	117
**16S recovered**	putative	no	no	no	no	no	no	no
**16S recovery software**	checkM	checkM	checkM	checkM	checkM	checkM	checkM	checkM
**total # tRNAs**	35	13	19	34	17	28	30	19
**# of standard tRNAs extracted**	17	9	14	20	12	14	15	13
**tRNA detection software**	tRNAscan-se	tRNAscan-se	tRNAscan-se	tRNAscan-se	tRNAscan-se	Aragorn	tRNAscan-se	tRNAscan-se
**length (Mb)**	1.371	3.345	0.964	1.38	3.29	7.74	2.48	0.991
**GC content(%)**	36.42	67.72	38.16	36.56%	52.44%	57.01	48.54	36.4
**# CDS**	1471	3636	1059	1445	3730	6862	2042	1125
**N50**	15073	10365	7066	18683	11157	26782	9929	8923
**completeness approach**	marker gene based	marker gene based	marker gene based	marker gene based	marker gene based	marker gene based	marker gene based	marker gene based
**decontamination software**	anvi'o	anvi'o	anvi'o	anvi'o	anvi'o	anvi'o	anvi'o	anvi'o
**bin parameters**	kmer+coverage+codon usage	kmer+coverage+codon usage	kmer+coverage+codon usage	kmer+coverage+codon usage	kmer+coverage+codon usage	kmer+coverage+codon usage	kmer+coverage+codon usage	kmer+coverage+codon usage
**binning software**	anvi'o	anvi'o	anvi'o	anvi'o	anvi'o	anvi'o	anvi'o	anvi'o
**reassembly post binning**	no	no	no	no	no	no	no	no
**mag coverage software**	anvi'o	anvi'o	anvi'o	anvi'o	anvi'o	anvi'o	anvi'o	anvi'o

Name	Acidobacteria_subdivision3_Amazon_FNV_2010_0_2_1	Bacteroidetes_Amazon_FNV_2010_21_8	Melainabacteria_Amazon_FNV_2010_23_1_1	Acidobacteria_Amazon_FNV_2010_25_4	Acidobacteria_subdivision4_Amazon_FNV_2010_3_1	Proteobacteria_Amazon_FNV_2010_19_4	Proteobacteria_Amazon_FNV_2010_31_3	Acidobacteria_Amazon_FNV_2010_39_1_1
**Analysis Project Type**	MAG	MAG	MAG	MAG	MAG	MAG	MAG	MAG
**Taxa ID approach**	multi marker approach	multi marker approach	multi marker approach	multi marker approach	multi marker approach	multi marker approach	multi marker approach	multi marker approach
**Taxa ID**	Bacteria	Bacteria	Bacteria	Bacteria	Bacteria	Bacteria	Bacteria	Bacteria
**Assembly software**	anvi'o	anvi'o	anvi'o	anvi'o	anvi'o	anvi'o	anvi'o	anvi'o
**Annotation pipeline/tool**	PATRIC/RAST	PATRIC/RAST	PATRIC/RAST	PATRIC/RAST	PATRIC/RAST	PATRIC/RAST	PATRIC/RAST	PATRIC/RAST
**Quality**	medium-quality	medium-quality	medium-quality	medium-quality	medium-quality	medium-quality	medium-quality	medium-quality
**Completion (%)**	68.03	67.24	63.73	59.67	58.77	55.51	53.51	52.59
**Contamination (%)**	5.93	3.42	1.16	0.85	3.6	3.36	0.18	2.35
**completeness software**	anvi'o	anvi'o	anvi'o	anvi'o	anvi'o	anvi'o	anvi'o	anvi'o
**number of contigs**	405	274	294	359	436	490	258	366
**16S recovered**	putative	putative	no	no	no	no	putative	no
**16S recovery software**	checkM	checkM	checkM	checkM	checkM	checkM	checkM	checkM
**total # tRNAs**	24	36	18	9	4	8	6	5
**# of standard tRNAs extracted**	13	16	11	8	3	6	4	5
**tRNA detection software**	Aragorn	tRNAscan-se	tRNAscan-se	tRNAscan-se	tRNAscan-se	tRNAscan-se	tRNAscan-se	tRNAscan-se
**length (Mb)**	7.44	4.43	3.04	2.6	3.57	3.34	2.13	3.04
**GC content(%)**	60.03	39.56	50.61	47.35	50.88	63.52	60.69	61.41
**# CDS**	6869	4119	2991	2630	3376	3545	2145	3065
**N50**	24087	20381	11292	7198	8339	6628	8155	8548
**completeness approach**	marker gene based	marker gene based	marker gene based	marker gene based	marker gene based	marker gene based	marker gene based	marker gene based
**decontamination software**	anvi'o	anvi'o	anvi'o	anvi'o	anvi'o	anvi'o	anvi'o	anvi'o
**bin parameters**	kmer+coverage+codon usage	kmer+coverage+codon usage	kmer+coverage+codon usage	kmer+coverage+codon usage	kmer+coverage+codon usage	kmer+coverage+codon usage	kmer+coverage+codon usage	kmer+coverage+codon usage
**binning software**	anvi'o	anvi'o	anvi'o	anvi'o	anvi'o	anvi'o	anvi'o	anvi'o
**reassembly post binning**	no	no	no	no	no	no	no	no
**mag coverage software**	anvi'o	anvi'o	anvi'o	anvi'o	anvi'o	anvi'o	anvi'o	anvi'o

Name	Acidobacteria_subdivision4_Amazon_FNV_2010_23_8	Acidobacteria_Amazon_FNV_2010_17_8	Bacteroidetes_Amazon_FNV_2010_13_3	Chloroflexi_Amazon_FNV_2010_25_1
**Analysis Project Type**	MAG	MAG	MAG	MAG
**Taxa ID approach**	multi marker approach	multi marker approach	multi marker approach	multi marker approach
**Taxa ID**	Bacteria	Bacteria	Bacteria	Bacteria
**Assembly software**	anvi'o	anvi'o	anvi'o	anvi'o
**Annotation pipeline/tool**	PATRIC/RAST	PATRIC/RAST	PATRIC/RAST	PATRIC/RAST
**Quality**	medium-quality	medium-quality	medium-quality	medium-quality
**Completion (%)**	51.96	51.59	51.39	51.38
**Contamination (%)**	0.81	1.57	0.81	2.47
**completeness software**	anvi'o	anvi'o	anvi'o	anvi'o
**number of contigs**	216	469	275	330
**16S recovered**	no	no	putative	no
**16S recovery software**	checkM	checkM	checkM	checkM
**total # tRNAs**	3	21	32	11
**# of standard tRNAs extracted**	2	10	14	8
**tRNA detection software**	tRNAscan-se	tRNAscan-se	tRNAscan-se	tRNAscan-se
**length (Mb)**	1.57	3.4	4.58	2.61
**GC content(%)**	51.8	55.46	42.76	58.44
**# CDS**	1582	3925	4733	2831
**N50**	7154	6914	22057	7631
**completeness approach**	marker gene based	marker gene based	marker gene based	marker gene based
**decontamination software**	anvi'o	anvi'o	anvi'o	anvi'o
**bin parameters**	kmer+coverage+codon usage	kmer+coverage+codon usage	kmer+coverage+codon usage	kmer+coverage+codon usage
**binning software**	anvi'o	anvi'o	anvi'o	anvi'o
**reassembly post binning**	no	no	no	no
**mag coverage software**	anvi'o	anvi'o	anvi'o	anvi'o
