# Supplementary material for: New Biological Insights Into How Deforestation in Amazonia Affects Soil Microbial Communities Using Metagenomics and Metagenome-Assembled Genomes
Source: Front Microbiol. 2018 Jul 23;9:1635. doi: 10.3389/fmicb.2018.01635 (PMC6064768; doi:10.3389/fmicb.2018.01635)
Supplement: Supplementary file 11 [file Table_5.PDF]

Supplemental Table 5: Genes involved in carbon cycling that are signficantly different between the 5 metagenomes of the rainforest and the 5 metagenomes of the cattle pasture (determined by DESeq2). In the associated heatmap the raw counts in each rainforest and pasture sample are listed with lowest to highest counts on a red via yellow to green color scale, respectively.

Functional Group	baseMean	log2FoldChange	lfcSE	stat	pvalue	padj	Level 1	Level 2	Level 3	Level 4	Forest_A001	Forest_A01	Forest_A1	Forest_A10	Forest_A100	Pasture_A001	Pasture_A01	Pasture_A1	Pasture_A10	Pasture_A100	land_use
Lignin Metabolism	287.138	-1.497	0.451	-3.320	0.000900166	0.009233	Iron acquisition and metabolism	-	Encapsulating_protein_for_DyP-type_peroxidase_and_ferritin-like_protein_oligomers	Encapsulating protein for a DyP-type peroxidase or ferritin-like protein oligomers	296	107	492	655	533	82	138	100	179	259	Forest
Lignin Metabolism	161.320	-1.578	0.401	-3.935	8.31E-05	0.001408	Stress Response	Oxidative stress	Oxidative_stress	Superoxide dismutase [Mn/Fe] (EC 1.15.1.1)	211	140	402	163	240	43	80	108	123	63	Forest
Aromatic Compounds	33.869	-3.234	0.471	-6.868	6.49E-12	6.07E-10	Metabolism of Aromatic Compounds	-	Benzoate_transport_and_degradation_cluster	Shikimate kinase II (EC 2.7.1.71)	18	27	73	144	46	2	3	8	3	12	Forest
Aromatic Compounds	7.149	-2.866	0.539	-5.322	1.03E-07	4.38E-06	Metabolism of Aromatic Compounds	Metabolism of central aromatic intermediates	Central_meta-cleavage_pathway_of_aromatic_compound_degradation	catalytic subunit of meta cleavage enzyme	13	4	21	13	10	0	2	0	0	5	Forest
Aromatic Compounds	342.460	-1.921	0.393	-4.890	1.01E-06	3.53E-05	Metabolism of Aromatic Compounds	-	carbazol_degradation_cluster	Ferredoxin component of carbazole 1,9a-dioxygenase	564	403	782	303	491	196	113	96	124	195	Forest
Aromatic Compounds	90.280	-1.685	0.415	-4.057	4.97E-05	0.000922	Metabolism of Aromatic Compounds	Peripheral pathways for catabolism of aromatic compounds	Benzoate_degradation	benzoate MFS transporter BenK	152	95	73	241	87	72	46	48	28	24	Forest
Aromatic Compounds	115.890	-1.391	0.362	-3.839	0.000123761	0.001921	Metabolism of Aromatic Compounds	-	Benzoate_transport_and_degradation_cluster	2-oxo-hepta-3-ene-1,7-dioic acid hydratase (EC 4.2.-.-)	205	101	190	171	113	55	69	77	54	78	Forest
Aromatic Compounds	666.037	-1.563	0.415	-3.770	0.000163448	0.002403	Metabolism of Aromatic Compounds	Metabolism of central aromatic intermediates	N-heterocyclic_aromatic_compound_degradation	Isoquinoline 1-oxidoreductase beta subunit (EC 1.3.99.16)	1436	406	1357	736	680	272	302	397	537	227	Forest
Aromatic Compounds	145.104	-1.482	0.399	-3.719	0.00019963	0.002821	Metabolism of Aromatic Compounds	Peripheral pathways for catabolism of aromatic compounds	Biphenyl_Degradation	Biphenyl dioxygenase system ferredoxin component	284	135	261	138	169	70	93	57	55	118	Forest
Aromatic Compounds	26.150	-1.135	0.313	-3.632	0.000281628	0.003688	Metabolism of Aromatic Compounds	Peripheral pathways for catabolism of aromatic compounds	Chlorobenzoate_degradation	2-chlorobenzoate 1,2-dioxygenase beta subunit (EC 1.14.12.13)	23	21	47	33	48	18	15	14	24	17	Forest
Aromatic Compounds	62.594	-1.636	0.455	-3.596	0.000323574	0.004147	Metabolism of Aromatic Compounds	-	Aromatic_Amin_Catabolism	3,4-dihydroxyphenylacetate 2,3-dioxygenase (EC 1.13.11.15)	123	74	133	21	88	24	46	19	56	11	Forest
Aromatic Compounds	25.333	-1.099	0.309	-3.555	0.000377479	0.004652	Metabolism of Aromatic Compounds	Peripheral pathways for catabolism of aromatic compounds	Benzoate_catabolism	Benzoate 1,2-dioxygenase beta subunit (EC 1.14.12.10)	34	24	38	25	40	16	14	14	21	21	Forest
Aromatic Compounds	116.519	-1.394	0.406	-3.437	0.000588612	0.006681	Metabolism of Aromatic Compounds	Metabolism of central aromatic intermediates	4-Hydroxyphenylacetic_acid_catabolic_pathway	2-oxo-hepta-3-ene-1,7-dioic acid hydratase (EC 4.2.-.-)	160	68	198	203	178	102	64	23	86	62	Forest
Aromatic Compounds	835.491	-1.851	0.567	-3.266	0.001090333	0.010722	Metabolism of Aromatic Compounds	-	Benzoate_transport_and_degradation_cluster	Benzoate transport, inner-membrane translocator	3587	806	578	230	395	465	122	280	623	189	Forest
Aromatic Compounds	791.505	-1.120	0.349	-3.209	0.001331185	0.01263	Metabolism of Aromatic Compounds	Anaerobic degradation of aromatic compounds	Anaerobic_benzoate_metabolism	Benzoate-CoA ligase (EC 6.2.1.25)	1238	621	1161	955	1066	532	686	306	528	607	Forest
Aromatic Compounds	142.971	-1.237	0.387	-3.194	0.001404718	0.013167	Metabolism of Aromatic Compounds	Metabolism of central aromatic intermediates	Catechol_branch_of_beta-ketoadipate_pathway	Muconate cycloisomerase (EC 5.5.1.1)	284	139	140	213	141	70	76	123	97	78	Forest
Aromatic Compounds	333.742	-1.747	0.554	-3.156	0.001597609	0.014521	Metabolism of Aromatic Compounds	Metabolism of central aromatic intermediates	Protocatechuate_branch_of_beta-ketoadipate_pathway	3-carboxy-cis,cis-muconate cycloisomerase (EC 5.5.1.2)	1029	501	475	41	246	160	70	81	271	143	Forest
Aromatic Compounds	278.708	-1.434	0.457	-3.139	0.001696077	0.01514	Metabolism of Aromatic Compounds	Peripheral pathways for catabolism of aromatic compounds	Salicylate_ester_degradation	salicylate esterase	639	160	380	346	348	244	184	121	54	159	Forest
Aromatic Compounds	59.595	-1.107	0.354	-3.124	0.001781084	0.015684	Metabolism of Aromatic Compounds	Peripheral pathways for catabolism of aromatic compounds	Benzoate_degradation	Benzoate 1,2-dioxygenase alpha subunit (EC 1.14.12.10)	88	51	88	60	92	39	22	46	64	30	Forest
Aromatic Compounds	155.824	-1.174	0.381	-3.084	0.002040977	0.017352	Metabolism of Aromatic Compounds	Peripheral pathways for catabolism of aromatic compounds	Benzoate_catabolism	Muconate cycloisomerase (EC 5.5.1.1)	197	161	250	191	219	75	71	57	144	157	Forest
Aromatic Compounds	87.961	-1.301	0.430	-3.027	0.002470729	0.019947	Metabolism of Aromatic Compounds	Peripheral pathways for catabolism of aromatic compounds	Benzoate_degradation	Ortho-halobenzoate 1,2-dioxygenase beta-ISP protein OhbA	174	153	97	112	32	57	53	29	78	48	Forest
Aromatic Compounds	13.109	-1.149	0.386	-2.974	0.002937051	0.022778	Metabolism of Aromatic Compounds	-	p-cymene_degradation	HCOMODA decarboxylase	11	31	20	17	6	12	10	4	8	9	Forest
Aromatic Compounds	269.495	-1.247	0.426	-2.928	0.003411048	0.025433	Metabolism of Aromatic Compounds	Peripheral pathways for catabolism of aromatic compounds	Biphenyl_Degradation	Large subunit naph/bph dioxygenase	495	215	437	238	373	226	77	120	278	136	Forest
Aromatic Compounds	453.449	-0.922	0.318	-2.899	0.00373929	0.027126	Metabolism of Aromatic Compounds	Metabolism of central aromatic intermediates	Salicylate_and_gentisate_catabolism	Fumarylacetoacetate (FAA) hydrolase (EC 4.1.1.68)	436	447	569	734	627	323	378	285	309	383	Forest
Aromatic Compounds	180.927	-1.407	0.487	-2.890	0.003849881	0.027693	Metabolism of Aromatic Compounds	Anaerobic degradation of aromatic compounds	Hydroxyaromatic_decarboxylase_family	Hydroxyaromatic non-oxidative decarboxylase protein C (EC 4.1.1.-)	382	179	454	88	119	109	68	37	177	113	Forest
Aromatic Compounds	43.139	-0.922	0.328	-2.813	0.004914914	0.033174	Metabolism of Aromatic Compounds	Peripheral pathways for catabolism of aromatic compounds	Biphenyl_Degradation	Acetaldehyde dehydrogenase (EC 1.2.1.10)	52	52	67	37	55	21	43	19	37	40	Forest
Aromatic Compounds	266.745	-1.172	0.417	-2.812	0.004924154	0.033201	Metabolism of Aromatic Compounds	Peripheral pathways for catabolism of aromatic compounds	Phenylpropanoid_compound_degradation	4-hydroxycinnamoyl CoA hydratase/lyase (Enoyl-CoA hydratase/lyase) (EC 4.2.1.17)	460	361	326	143	397	151	111	234	162	189	Forest
Aromatic Compounds	10.340	-1.053	0.379	-2.781	0.005423946	0.035614	Metabolism of Aromatic Compounds	-	Benzoate_transport_and_degradation_cluster	FIG094199: Fumarylacetoacetate hydrolase	6	10	22	18	12	8	11	7	4	6	Forest
Aromatic Compounds	177.622	-1.325	0.477	-2.776	0.005495104	0.035934	Metabolism of Aromatic Compounds	Metabolism of central aromatic intermediates	Catechol_branch_of_beta-ketoadipate_pathway	Catechol 1,2-dioxygenase 1 (EC 1.13.11.1)	211	132	483	46	354	84	84	120	160	69	Forest
Aromatic Compounds	45.584	-0.989	0.359	-2.752	0.005925855	0.037954	Metabolism of Aromatic Compounds	Metabolism of central aromatic intermediates	4-Hydroxyphenylacetic_acid_catabolic_pathway	4-hydroxyphenylacetate symporter, major facilitator superfamily (MFS)	40	36	129	51	38	37	41	24	36	26	Forest
Aromatic Compounds	123.092	-0.981	0.364	-2.691	0.007130166	0.043412	Metabolism of Aromatic Compounds	-	carbazol_degradation_cluster	Terminal dioxygenase component of carbazole 1,9a-dioxygenase	148	151	236	117	115	71	69	67	105	127	Forest
Aromatic Compounds	346.017	-1.079	0.403	-2.680	0.007353428	0.044391	Metabolism of Aromatic Compounds	-	Gentisare_degradation	Gentisate 1,2-dioxygenase (EC 1.13.11.4)	297	390	547	291	716	237	146	212	176	384	Forest
Aromatic Compounds	17.664	-0.849	0.319	-2.659	0.007825677	0.046382	Metabolism of Aromatic Compounds	Peripheral pathways for catabolism of aromatic compounds	Naphtalene_and_antracene_degradation	Naphthalene 1,2-dioxygenase system ferredoxin--NAD(+) reductase component (EC 1.18.1.3)	23	17	25	20	21	12	20	8	16	12	Forest
Aromatic Compounds	6.546	-1.242	0.469	-2.649	0.008069983	0.047355	Metabolism of Aromatic Compounds	Peripheral pathways for catabolism of aromatic compounds	Toluene_degradation	Toluate 1,2-dioxygenase beta subunit	8	6	2	16	12	5	8	3	2	2	Forest
Aromatic Compounds	346.520	-1.084	0.410	-2.645	0.008173614	0.047766	Metabolism of Aromatic Compounds	-	Benzoate_transport_and_degradation_cluster	benzoate degradation ring-cleavage hydrolase	510	388	452	638	219	217	204	107	369	280	Forest
Aromatic Compounds	17.610	3.335	0.440	7.578	3.50E-14	4.35E-12	Metabolism of Aromatic Compounds	Peripheral pathways for catabolism of aromatic compounds	Salicylate_ester_degradation	putative facilitator of salicylate uptake	3	2	4	1	2	37	28	27	33	52	Pasture
Aromatic Compounds	16.169	3.214	0.439	7.329	2.32E-13	2.68E-11	Metabolism of Aromatic Compounds	Metabolism of central aromatic intermediates	Salicylate_and_gentisate_catabolism	putative facilitator of salicylate uptake	3	2	3	1	3	25	24	35	35	42	Pasture
Aromatic Compounds	12.068	3.570	0.535	6.674	2.50E-11	2.09E-09	Metabolism of Aromatic Compounds	Peripheral pathways for catabolism of aromatic compounds	Quinate_degradation	Quinate permease	0	2	2	2	0	31	49	16	21	10	Pasture
Aromatic Compounds	6.947	2.460	0.510	4.823	1.41E-06	4.72E-05	Metabolism of Aromatic Compounds	-	p-cymene_degradation	p-cymene degradation operon transcriptional regulator	3	1	0	2	2	9	19	15	13	10	Pasture
Aromatic Compounds	7.504	2.175	0.481	4.521	6.17E-06	0.000166	Metabolism of Aromatic Compounds	Peripheral pathways for catabolism of aromatic compounds	Quinate_degradation	Repressor Protein of Quinate Catabolism	2	2	2	3	2	17	23	10	14	6	Pasture
Aromatic Compounds	16.074	2.900	0.650	4.462	8.14E-06	0.000208	Metabolism of Aromatic Compounds	Anaerobic degradation of aromatic compounds	Anaerobic_toluene_and_ethylbenzene_degradation	Succinyl-CoA:(R)-benzylsuccinate CoA-transferase subunit BbsE (EC 2.8.3.15)	0	0	5	0	6	38	41	33	11	39	Pasture
Aromatic Compounds	17.306	1.593	0.365	4.364	1.28E-05	0.000297	Metabolism of Aromatic Compounds	Peripheral pathways for catabolism of aromatic compounds	Quinate_degradation	Protein QA-X	1	12	14	7	5	36	39	21	29	21	Pasture
Aromatic Compounds	142.613	1.698	0.399	4.256	2.08E-05	0.000449	Metabolism of Aromatic Compounds	Anaerobic degradation of aromatic compounds	Anaerobic_benzoate_metabolism	Benzoyl-CoA reductase subunit BadF (EC 1.3.99.15)	65	74	38	38	69	262	297	124	203	328	Pasture
Aromatic Compounds	26.273	1.411	0.332	4.246	2.17E-05	0.000467	Metabolism of Aromatic Compounds	-	Toluene_4-monooxygenase_(T4MO)	Toluene-4-monooxygenase, subunit TmoF	6	15	10	13	21	28	45	52	39	46	Pasture
Aromatic Compounds	86.518	1.501	0.367	4.091	4.29E-05	0.000812	Metabolism of Aromatic Compounds	Anaerobic degradation of aromatic compounds	Anaerobic_benzoate_metabolism	Benzoyl-CoA reductase subunit BadD (EC 1.3.99.15)	48	25	62	28	36	103	107	165	157	172	Pasture
Aromatic Compounds	6.914	1.904	0.471	4.043	5.27E-05	0.00097	Metabolism of Aromatic Compounds	Anaerobic degradation of aromatic compounds	Anaerobic_toluene_and_ethylbenzene_degradation	(R)-benzylsuccinyl-CoA dehydrogenase (EC 1.3.99.21)	3	1	1	2	5	12	15	14	10	10	Pasture
Aromatic Compounds	21.639	1.440	0.362	3.979	6.92E-05	0.001223	Metabolism of Aromatic Compounds	-	p-cymene_degradation	2-hydroxypenta-2,4-dienoate hydratase (CmtF) (EC 4.2.1.-)	11	8	6	19	8	48	36	19	54	21	Pasture
Aromatic Compounds	24.362	1.103	0.312	3.529	0.000417524	0.005053	Metabolism of Aromatic Compounds	Peripheral pathways for catabolism of aromatic compounds	Phenol_hydroxylase	Phenol hydroxylase, FAD- and [2Fe-2S]-containing reductase component DmpP	9	15	9	23	15	39	45	35	35	30	Pasture
Aromatic Compounds	1.665	2.724	0.784	3.473	0.000514967	0.006008	Metabolism of Aromatic Compounds	Anaerobic degradation of aromatic compounds	Anaerobic_toluene_and_ethylbenzene_degradation	Benzylsuccinate synthase activating enzyme (EC 1.97.1.4)	0	0	0	0	0	2	5	1	2	8	Pasture
Aromatic Compounds	7.634	1.457	0.433	3.365	0.00076507	0.008204	Metabolism of Aromatic Compounds	Peripheral pathways for catabolism of aromatic compounds	Phenol_hydroxylase	Phenol hydroxylase, assembly protein DmpK	2	3	2	8	3	11	17	13	13	9	Pasture
Aromatic Compounds	13.688	1.188	0.361	3.291	0.000997074	0.010017	Metabolism of Aromatic Compounds	-	Phenylacetyl-CoA_catabolic_pathway_(core)	Putative membrane protein, clustering with ActP PaaL	8	11	9	5	4	20	23	25	13	23	Pasture
Aromatic Compounds	50.389	1.254	0.392	3.196	0.001392696	0.013073	Metabolism of Aromatic Compounds	-	Benzoate_transport_and_degradation_cluster	hydroxybenzoate permease	4	28	25	53	28	91	62	94	95	54	Pasture
Aromatic Compounds	33.179	1.094	0.349	3.140	0.001690334	0.015135	Metabolism of Aromatic Compounds	Metabolism of central aromatic intermediates	Central_meta-cleavage_pathway_of_aromatic_compound_degradation	2-hydroxymuconic semialdehyde dehydrogenase	10	34	29	14	9	49	42	50	60	49	Pasture
Aromatic Compounds	55.792	1.150	0.367	3.131	0.001743846	0.015441	Metabolism of Aromatic Compounds	Peripheral pathways for catabolism of aromatic compounds	Phenylpropanoid_compound_degradation	Tannase and feruloyl esterase precursor	17	43	39	43	16	95	130	89	64	50	Pasture
Aromatic Compounds	141.572	0.922	0.326	2.827	0.004698151	0.032137	Metabolism of Aromatic Compounds	Peripheral pathways for catabolism of aromatic compounds	Naphtalene_and_antracene_degradation	naphthalene dioxygenase ferredoxin	83	88	84	106	83	151	218	241	223	187	Pasture
Aromatic Compounds	12.162	0.975	0.353	2.761	0.005763226	0.037056	Metabolism of Aromatic Compounds	-	Benzoate_transport_and_degradation_cluster	Putative Acyl-CoA thiolase	2	9	12	8	7	18	20	15	20	17	Pasture
Aromatic Compounds	1.107	2.177	0.799	2.724	0.006441318	0.04031	Metabolism of Aromatic Compounds	Anaerobic degradation of aromatic compounds	Anaerobic_toluene_and_ethylbenzene_degradation	Benzylsuccinate synthase alpha subunit (EC 4.1.99.11)	0	0	0	0	0	2	5	2	0	3	Pasture
Aromatic Compounds	34.094	0.847	0.314	2.693	0.007082384	0.043194	Metabolism of Aromatic Compounds	Peripheral pathways for catabolism of aromatic compounds	Biphenyl_Degradation	2-hydroxypenta-2,4-dienoate hydratase	17	18	25	35	18	38	68	51	55	31	Pasture
-	31.170	-2.775	0.405	-6.852	7.31E-12	6.78E-10	Carbohydrates	-	Sugar_utilization_in_Thermotogales	Predicted keto-inosose hydrolase	31	41	103	25	63	8	4	6	6	14	Forest
-	48.995	-1.461	0.324	-4.509	6.53E-06	0.000174	Carbohydrates	-	Unknown_sugar_utilization_(cluster_yphABCDEFG)	Inner membrane protein YphA	72	52	90	55	67	30	20	19	35	34	Forest
-	175.320	-1.958	0.441	-4.437	9.12E-06	0.000229	Carbohydrates	-	Sugar_utilization_in_Thermotogales	Polygalacturonase (EC 3.2.1.15)	373	254	328	269	67	66	113	67	57	53	Forest
-	34.553	-1.500	0.344	-4.355	1.33E-05	0.000308	Carbohydrates	-	Sugar_utilization_in_Thermotogales	Myo-inositol ABC transporter, permease protein InoF	58	55	57	24	40	21	19	16	14	24	Forest
-	12.648	-1.399	0.375	-3.735	0.000187766	0.002682	Carbohydrates	-	Sugar_utilization_in_Thermotogales	Myo-inositol ABC transporter, permease protein InoG	20	20	24	11	10	6	5	10	9	6	Forest
-	239.654	-1.738	0.515	-3.374	0.000741729	0.008059	Carbohydrates	-	Sugar_utilization_in_Thermotogales	Alpha-L-fucosidase (EC 3.2.1.51)	748	372	154	196	154	90	163	62	190	39	Forest
-	93.533	-1.046	0.331	-3.158	0.001590463	0.014474	Carbohydrates	-	Sugar_utilization_in_Thermotogales	Myo-inositol ABC transporter, ATP-binding protein InoK	105	104	136	93	153	48	88	71	57	61	Forest
-	45.988	-1.224	0.393	-3.114	0.001846017	0.016079	Carbohydrates	-	Sugar_utilization_in_Thermotogales	Predicted glycosylase, COG2152	41	21	149	42	63	24	32	29	34	26	Forest
-	319.688	-1.373	0.463	-2.964	0.003038413	0.02338	Carbohydrates	-	Unknown_carbohydrate_utilization_(_cluster_Yeg_)	Fructose-bisphosphate aldolase class I (EC 4.1.2.13)	460	306	587	184	644	42	188	206	251	216	Forest
-	49.836	-1.100	0.388	-2.832	0.004632972	0.031824	Carbohydrates	-	Sugar_utilization_in_Thermotogales	Ribose ABC transport system, periplasmic ribose-binding protein RbsB (TC 3.A.1.2.1)	44	40	26	99	116	22	33	29	40	43	Forest
-	20.750	1.872	0.391	4.784	1.72E-06	5.59E-05	Carbohydrates	-	Sugar_utilization_in_Thermotogales	Extracellular endo-1,4-glucanase	4	9	4	16	6	23	51	57	31	19	Pasture
-	21.787	1.217	0.330	3.690	0.000224633	0.003083	Carbohydrates	-	Sugar_utilization_in_Thermotogales	Predicted glycerol-1-phosphate dehydrogenase, arabinose operon	6	21	8	10	14	38	30	36	34	30	Pasture
-	43.539	1.215	0.375	3.245	0.001176026	0.011402	Carbohydrates	-	Sugar_utilization_in_Thermotogales	Glucose oligosaccharide ABC transport system, permease protein 2	5	42	23	24	25	61	62	81	91	44	Pasture
-	29.545	1.149	0.373	3.079	0.002074533	0.017533	Carbohydrates	-	Sugar_utilization_in_Thermotogales	Xylose oligosaccharides ABC transporter, permease protein 2	8	20	8	18	29	47	78	42	41	20	Pasture
-	1.705	2.217	0.765	2.900	0.003733862	0.027102	Carbohydrates	-	Sugar_utilization_in_Thermotogales	Alpha-1,4-digalacturonate ABC transporter, substrate-binding protein	0	0	0	0	1	1	2	3	2	9	Pasture
-	12.887	1.129	0.395	2.859	0.00425604	0.029927	Carbohydrates	-	Sugar_utilization_in_Thermotogales	Predicted L-rhamnose mutarotase	4	3	12	6	12	26	30	8	11	24	Pasture
-	3.844	1.653	0.580	2.851	0.004352259	0.030385	Carbohydrates	-	Sugar_utilization_in_Thermotogales	Myo-inositol ABC transporter, periplasmic sugar-binding protein InoE	4	1	1	1	0	3	6	9	11	4	Pasture
-	2.987	1.780	0.628	2.835	0.004575595	0.031532	Carbohydrates	-	Uncharacterized_sugar_kinase_cluster_(ygc)	Probable electron transfer flavoprotein-quinone oxidoreductase YgcN (EC 1.5.5.-)	0	1	2	2	0	4	2	7	8	6	Pasture
-	42.183	0.965	0.349	2.768	0.0056319	0.036549	Carbohydrates	-	Sugar_utilization_in_Thermotogales	Predicted cellobiose ABC transport system, permease protein 2	8	34	34	36	21	80	50	58	44	75	Pasture
-	5.439	1.384	0.511	2.708	0.006778473	0.041767	Carbohydrates	-	Unknown_carbohydrate_utilization_(_cluster_Ydj_)	Hypothetical oxidoreductase YdjG (EC 1.-.-.-)	1	1	4	4	3	8	9	7	2	18	Pasture
-	72.175	1.001	0.371	2.700	0.006924204	0.042501	Carbohydrates	-	Acetone_carboxylase	Acetone carboxylase, alpha subunit (EC 6.4.1.6)	39	37	61	56	26	112	109	138	136	41	Pasture
sugar alcohols	80.643	-2.584	0.435	-5.934	2.96E-09	1.74E-07	Carbohydrates	Sugar alcohols	Propanediol_utilization	Cob(III)alamin reductase	169	71	146	60	196	11	30	15	16	37	Forest
sugar alcohols	115.709	-1.422	0.374	-3.797	0.00014652	0.002205	Carbohydrates	Sugar alcohols	Glycerol_and_Glycerol-3-phosphate_Uptake_and_Utilization	Aerobic glycerol-3-phosphate dehydrogenase (EC 1.1.5.3)	160	80	179	156	222	58	41	87	61	78	Forest
sugar alcohols	214.002	-1.812	0.580	-3.124	0.001785742	0.015704	Carbohydrates	Sugar alcohols	Inositol_catabolism	5-keto-2-deoxygluconokinase (EC 2.7.1.92)	117	549	903	31	62	62	127	103	45	90	Forest
sugar alcohols	38.570	-0.816	0.302	-2.699	0.006955177	0.042569	Carbohydrates	Sugar alcohols	Erythritol_utilization	Erythritol transcriptional regulator EryD	32	43	58	47	53	39	23	35	35	19	Forest
sugar alcohols	89.842	-1.274	0.484	-2.631	0.008509728	0.049256	Carbohydrates	Sugar alcohols	Glycerol_and_Glycerol-3-phosphate_Uptake_and_Utilization	Glycerol-3-phosphate ABC transporter, periplasmic glycerol-3-phosphate-binding protein (TC 3.A.1.1.3)	130	29	276	73	109	63	30	108	32	30	Forest
sugar alcohols	8.299	1.803	0.445	4.048	5.16E-05	0.000954	Carbohydrates	Sugar alcohols	Glycerol_fermenation_to_1,3-propanediol	Glycerol dehydratase reactivation factor large subunit	2	1	8	3	2	18	8	13	21	13	Pasture
sugar alcohols	19.744	1.249	0.318	3.925	8.69E-05	0.001453	Carbohydrates	Sugar alcohols	Glycerol_and_Glycerol-3-phosphate_Uptake_and_Utilization	Anaerobic glycerol-3-phosphate dehydrogenase subunit B (EC 1.1.5.3)	4	10	14	14	12	29	30	27	32	36	Pasture
sugar alcohols	2.979	2.635	0.685	3.846	0.00012022	0.001877	Carbohydrates	Sugar alcohols	Propanediol_utilization	Propanediol utilization polyhedral body protein PduU	0	0	0	1	1	3	4	8	7	8	Pasture
sugar alcohols	6.968	1.741	0.469	3.716	0.00020202	0.002849	Carbohydrates	Sugar alcohols	Ethanolamine_utilization	Ethanolamine utilization polyhedral-body-like protein EutS	7	1	1	2	2	12	11	13	10	13	Pasture
sugar alcohols	6.141	1.694	0.484	3.498	0.000468502	0.005585	Carbohydrates	Sugar alcohols	Ethanolamine_utilization	Ethanolamine utilization protein EutP	3	2	4	1	2	7	8	15	9	13	Pasture
sugar alcohols	8.862	1.282	0.404	3.173	0.001509396	0.013874	Carbohydrates	Sugar alcohols	Glycerol_and_Glycerol-3-phosphate_Uptake_and_Utilization	Probable glycerol transport protein	2	7	5	3	6	9	15	15	18	13	Pasture
sugar alcohols	11.844	1.167	0.372	3.141	0.001684552	0.015104	Carbohydrates	Sugar alcohols	Mannitol_Utilization	Mannitol operon activator, BglG family	5	5	8	10	5	18	15	25	11	21	Pasture
sugar alcohols	34.475	1.096	0.349	3.138	0.001701263	0.01514	Carbohydrates	Sugar alcohols	Erythritol_utilization	Predicted erythritol ABC transporter 1, ATP-binding component 1	14	18	14	26	28	44	63	77	54	22	Pasture
sugar alcohols	4.407	1.604	0.538	2.983	0.002854343	0.022339	Carbohydrates	Sugar alcohols	Ethanolamine_utilization	ATP:Cob(I)alamin adenosyltransferase (EC 2.5.1.17), ethanolamine utilization	0	2	0	2	5	9	8	5	8	8	Pasture
sugar alcohols	25.686	1.029	0.348	2.954	0.003140159	0.023978	Carbohydrates	Sugar alcohols	Mannitol_Utilization	PTS system, mannitol-specific IIB component (EC 2.7.1.69)	6	12	13	15	32	38	40	51	39	23	Pasture
sugar alcohols	1.186	2.222	0.799	2.782	0.005395562	0.035486	Carbohydrates	Sugar alcohols	Ethanolamine_utilization	Protein clustered with ethanolamine utilization	0	0	0	0	0	0	1	1	6	5	Pasture
sugar alcohols	4.796	1.408	0.515	2.733	0.006277951	0.0395	Carbohydrates	Sugar alcohols	Mannitol_Utilization	PTS system, mannitol-specific cryptic IIC component (EC 2.7.1.69)	2	3	3	2	1	3	11	9	6	10	Pasture
sugar alcohols	3.141	1.622	0.609	2.662	0.007760353	0.046089	Carbohydrates	Sugar alcohols	Ethanolamine_utilization	Ethanolamine utilization protein similar to PduV	1	0	2	1	2	4	5	3	12	4	Pasture
sugar alcohols	22.114	0.817	0.309	2.647	0.008112346	0.047506	Carbohydrates	Sugar alcohols	Propanediol_utilization	Propanediol dehydratase medium subunit (EC 4.2.1.28)	9	16	20	14	15	21	30	35	34	35	Pasture
polysaccharide	118.905	-1.201	0.355	-3.382	0.000720873	0.00788	Carbohydrates	Polysaccharides	Glycogen_metabolism	Glycogen synthase, ADP-glucose transglucosylase (EC 2.4.1.21)	218	111	139	157	135	90	75	55	82	80	Forest
polysaccharide	362.895	-1.534	0.504	-3.043	0.002345575	0.019108	Carbohydrates	Polysaccharides	Glycogen_metabolism	Glycogen phosphorylase (EC 2.4.1.1)	1121	351	452	268	206	358	81	171	203	111	Forest
polysaccharide	20.750	1.872	0.391	4.784	1.72E-06	5.59E-05	Carbohydrates	Polysaccharides	Xyloglucan_Utilization	Extracellular endo-1,4-glucanase	4	9	4	16	6	23	51	57	31	19	Pasture
polysaccharide	8.313	2.327	0.495	4.699	2.62E-06	8.00E-05	Carbohydrates	Polysaccharides	Cellulosome	Alpha-amylase SusG (EC 3.2.1.1)	2	0	7	2	0	5	15	17	20	21	Pasture
polysaccharide	47.051	1.416	0.343	4.125	3.71E-05	0.000721	Carbohydrates	Polysaccharides	Cellulosome	Alpha-glucosidase SusB (EC 3.2.1.20)	20	21	27	39	9	60	77	80	99	64	Pasture
polysaccharide	43.369	1.227	0.357	3.433	0.000597259	0.006762	Carbohydrates	Polysaccharides	Xyloglucan_Utilization	Glucose oligosaccharide ABC transport system, permease protein 2	5	37	28	24	25	71	62	68	91	47	Pasture
polysaccharide	337.832	1.090	0.374	2.916	0.003547497	0.02615	Carbohydrates	Polysaccharides	Cellulosome	Endoglucanase J, the largest catalytic component of the cellulosome	77	275	218	257	164	497	552	577	502	418	Pasture
organic acids	340.090	-1.484	0.383	-3.879	0.000104818	0.001676	Carbohydrates	Organic acids	Lactate_utilization	L-lactate permease	570	371	615	553	236	183	190	192	135	224	Forest
organic acids	149.258	-1.433	0.386	-3.714	0.000204193	0.002873	Carbohydrates	Organic acids	Glycerate_metabolism	2-hydroxy-3-oxopropionate reductase (EC 1.1.1.60)	167	334	123	183	202	62	71	88	88	108	Forest
organic acids	42.426	-1.324	0.370	-3.578	0.000345923	0.004351	Carbohydrates	Organic acids	Alpha-acetolactate_operon	Acetolactate synthase, catabolic (EC 2.2.1.6)	53	34	89	24	87	28	26	17	23	33	Forest
organic acids	19.818	-1.144	0.338	-3.386	0.000710296	0.0078	Carbohydrates	Organic acids	Lactate_utilization	Lactate-responsive regulator LldR in Actinobacteria, GntR family	19	18	51	21	22	17	15	9	11	14	Forest
organic acids	133.543	-1.234	0.409	-3.014	0.002578543	0.020674	Carbohydrates	Organic acids	Tricarballylate_Utilization	TcuB: works with TcuA to oxidize tricarballylate to cis-aconitate	198	99	219	77	285	72	103	78	112	54	Forest
organic acids	209.816	-0.957	0.344	-2.782	0.005399962	0.035493	Carbohydrates	Organic acids	Propionyl-CoA_to_Succinyl-CoA_Module	Biotin carboxylase of Propionyl-CoA carboxylase (EC 6.3.4.14)	265	192	230	253	352	125	171	157	186	125	Forest
organic acids	560.955	-1.175	0.436	-2.693	0.007084243	0.043194	Carbohydrates	Organic acids	Propionate-CoA_to_Succinate_Module	Aconitate hydratase (EC 4.2.1.3)	758	731	988	984	223	427	373	561	187	224	Forest
organic acids	31.540	1.204	0.357	3.374	0.000742103	0.008059	Carbohydrates	Organic acids	Malonate_decarboxylase	Malonate transporter, MadL subunit	11	16	20	20	20	39	91	57	37	21	Pasture
organic acids	22.856	1.122	0.346	3.243	0.001184735	0.011449	Carbohydrates	Organic acids	Malonate_decarboxylase	Malonate decarboxylase gamma subunit	8	13	17	16	12	65	29	29	35	17	Pasture
organic acids	27.135	0.972	0.309	3.144	0.001666811	0.014998	Carbohydrates	Organic acids	Malonate_decarboxylase	Triphosphoribosyl-dephospho-CoA synthetase (EC 2.7.8.25)	9	14	17	21	24	39	40	26	45	49	Pasture
monosaccharide	294.979	-2.041	0.512	-3.985	6.75E-05	0.001195	Carbohydrates	Monosaccharides	Mannose_Metabolism	Phosphomannomutase (EC 5.4.2.8)	851	529	211	98	414	135	65	55	113	171	Forest
monosaccharide	136.906	-1.371	0.363	-3.779	0.000157683	0.002334	Carbohydrates	Monosaccharides	L-rhamnose_utilization	L-rhamnose operon transcriptional activator RhaR	144	96	243	203	262	65	72	106	94	63	Forest
monosaccharide	54.723	-1.303	0.431	-3.024	0.002491716	0.020091	Carbohydrates	Monosaccharides	D-gluconate_and_ketogluconates_metabolism	Gluconate 2-dehydrogenase (EC 1.1.99.3), membrane-bound, cytochrome c	60	50	30	84	146	24	73	25	28	15	Forest
monosaccharide	191.648	-1.661	0.555	-2.992	0.00277203	0.021896	Carbohydrates	Monosaccharides	D-gluconate_and_ketogluconates_metabolism	Gluconate 2-dehydrogenase (EC 1.1.99.3), membrane-bound, gamma subunit	606	91	325	249	60	69	132	29	190	28	Forest
monosaccharide	114.564	-1.191	0.410	-2.906	0.003666182	0.026732	Carbohydrates	Monosaccharides	Xylose_utilization	Xylose ABC transporter, ATP-binding component	105	209	231	117	95	94	125	31	72	48	Forest
monosaccharide	107.660	-0.874	0.319	-2.737	0.006206055	0.039202	Carbohydrates	Monosaccharides	Deoxyribose_and_Deoxynucleoside_Catabolism	Predicted nucleoside ABC transporter, substrate-binding component	130	99	129	128	164	97	70	83	85	73	Forest
monosaccharide	97.540	-1.274	0.484	-2.635	0.008406029	0.04879	Carbohydrates	Monosaccharides	D-ribose_utilization	Ribose 5-phosphate isomerase B (EC 5.3.1.6)	184	147	78	45	174	27	19	104	88	49	Forest
monosaccharide	35.691	2.242	0.357	6.275	3.50E-10	2.37E-08	Carbohydrates	Monosaccharides	2-Ketogluconate_Utilization	HTH-type transcriptional regulator PtxR	6	17	13	8	10	63	91	49	91	37	Pasture
monosaccharide	11.971	2.290	0.424	5.404	6.53E-08	2.91E-06	Carbohydrates	Monosaccharides	D-Tagatose_and_Galactitol_Utilization	Predicted galactitol operon regulator (Transcriptional antiterminator), BglG family	2	2	3	3	7	21	14	31	30	15	Pasture
monosaccharide	4.369	3.883	0.731	5.308	1.11E-07	4.66E-06	Carbohydrates	Monosaccharides	D-Galacturonate_and_D-Glucuronate_Utilization	Glucuronide permease	0	0	0	0	0	4	8	21	9	5	Pasture
monosaccharide	6.167	2.501	0.530	4.718	2.38E-06	7.39E-05	Carbohydrates	Monosaccharides	D-Galacturonate_and_D-Glucuronate_Utilization	Fructuronate transporter GntP	0	0	2	4	1	15	9	12	14	10	Pasture
monosaccharide	20.221	1.739	0.403	4.319	1.57E-05	0.000354	Carbohydrates	Monosaccharides	L-Arabinose_utilization	Predicted L-arabinose isomerase (EC 5.3.1.4)	19	7	2	5	5	25	28	38	32	47	Pasture
monosaccharide	30.368	1.475	0.347	4.246	2.18E-05	0.000467	Carbohydrates	Monosaccharides	Xylose_utilization	Xylose oligosaccharides ABC transporter, permease protein 2	8	17	8	14	25	54	78	40	42	35	Pasture
monosaccharide	10.644	1.560	0.390	4.003	6.27E-05	0.001132	Carbohydrates	Monosaccharides	D-Sorbitol(D-Glucitol)_and_L-Sorbose_Utilization	Putative transcriptional regulator of sorbose uptake and utilization genes	5	1	9	6	3	19	18	14	21	17	Pasture
monosaccharide	23.694	1.302	0.327	3.985	6.75E-05	0.001195	Carbohydrates	Monosaccharides	Xylose_utilization	predicted xylose isomerase	13	13	9	14	12	35	27	38	31	53	Pasture
monosaccharide	21.576	1.325	0.351	3.776	0.00015953	0.002353	Carbohydrates	Monosaccharides	L-Arabinose_utilization	Hypothetical glycoside hydrolase, family 43, similar to arabinosidase	6	11	10	17	12	19	39	51	42	20	Pasture
monosaccharide	59.248	1.332	0.360	3.703	0.000212694	0.002971	Carbohydrates	Monosaccharides	Mannose_Metabolism	Endo-alpha-mannosidase	17	50	42	21	21	64	101	110	106	88	Pasture
monosaccharide	79.755	1.548	0.422	3.664	0.000248716	0.003349	Carbohydrates	Monosaccharides	D-Galacturonate_and_D-Glucuronate_Utilization	D-mannonate oxidoreductase (EC 1.1.1.57)	18	51	34	38	37	49	69	143	167	227	Pasture
monosaccharide	24.031	1.159	0.317	3.654	0.00025778	0.003431	Carbohydrates	Monosaccharides	Xylose_utilization	Possible alpha-xyloside ABC transporter, substrate-binding component	10	11	19	11	17	50	38	37	25	33	Pasture
monosaccharide	29.663	1.128	0.314	3.592	0.00032799	0.004187	Carbohydrates	Monosaccharides	Xylose_utilization	D-xylose proton-symporter XylT	14	24	17	15	14	41	34	55	57	37	Pasture
monosaccharide	18.186	1.255	0.350	3.582	0.000340375	0.004298	Carbohydrates	Monosaccharides	D-galactarate,_D-glucarate_and_D-glycerate_catabolism	Predicted D-glucarate or D-galactorate regulator, IclR family	6	11	17	9	6	36	25	22	45	16	Pasture
monosaccharide	31.748	1.142	0.319	3.578	0.000346672	0.004356	Carbohydrates	Monosaccharides	Fructose_utilization	Transcriptional repressor of the fructose operon, DeoR family	18	23	19	18	11	53	67	46	46	30	Pasture
monosaccharide	72.819	1.695	0.498	3.404	0.000663097	0.007383	Carbohydrates	Monosaccharides	L-Arabinose_utilization	Alpha-L-arabinofuranosidase II precursor (EC 3.2.1.55)	25	37	14	18	46	229	263	51	72	25	Pasture
monosaccharide	56.726	1.222	0.359	3.403	0.000665955	0.007402	Carbohydrates	Monosaccharides	D-gluconate_and_ketogluconates_metabolism	L-idonate, D-gluconate, 5-keto-D-gluconate transporter	17	28	46	29	36	62	46	111	132	88	Pasture
monosaccharide	8.263	1.406	0.419	3.357	0.000786922	0.008369	Carbohydrates	Monosaccharides	D-galactarate,_D-glucarate_and_D-glycerate_catabolism	Predicted 5-dehydro-4-deoxyglucarate regulator YcbG	2	5	3	6	4	14	10	18	16	9	Pasture
monosaccharide	9.142	1.338	0.401	3.339	0.000841925	0.008743	Carbohydrates	Monosaccharides	D-gluconate_and_ketogluconates_metabolism	Gluconate operon transcriptional repressor	5	2	5	5	6	12	22	13	13	13	Pasture
monosaccharide	7.449	1.402	0.433	3.238	0.001201886	0.011584	Carbohydrates	Monosaccharides	D-Sorbitol(D-Glucitol)_and_L-Sorbose_Utilization	Glucitol operon repressor	2	3	4	5	4	10	11	17	9	13	Pasture
monosaccharide	149.949	1.364	0.441	3.090	0.002003128	0.017087	Carbohydrates	Monosaccharides	L-Arabinose_utilization	Alpha-N-arabinofuranosidase (EC 3.2.1.55)	90	72	65	56	74	92	257	126	544	211	Pasture
monosaccharide	15.895	1.036	0.343	3.017	0.002554201	0.02053	Carbohydrates	Monosaccharides	Mannose_Metabolism	Transcriptional regulator of mannoside utilization, variant 2, LacI family	8	11	6	9	13	15	22	28	34	19	Pasture
monosaccharide	1.191	2.348	0.796	2.951	0.00316699	0.024052	Carbohydrates	Monosaccharides	D-Sorbitol(D-Glucitol)_and_L-Sorbose_Utilization	Glucitol/sorbitol-specific transport protein GutA	0	0	0	0	0	2	4	2	2	3	Pasture
monosaccharide	24.831	0.951	0.323	2.944	0.003242451	0.024493	Carbohydrates	Monosaccharides	D-galactarate,_D-glucarate_and_D-glycerate_catabolism	Predicted D-glucarate or D-galactorate regulator, GntR family	6	23	16	17	16	36	30	45	45	25	Pasture
monosaccharide	5.269	1.443	0.496	2.911	0.003607011	0.026395	Carbohydrates	Monosaccharides	L-ascorbate_utilization_(and_related_gene_clusters)	Probable L-ascorbate-6-phosphate lactonase UlaG (EC 3.1.1.-) (L-ascorbate utilization protein G)	2	2	2	3	3	5	8	14	8	8	Pasture
monosaccharide	43.841	1.254	0.431	2.906	0.003664234	0.026732	Carbohydrates	Monosaccharides	L-Arabinose_utilization	L-arabinose transport system permease protein (TC 3.A.1.2.2)	7	51	19	22	15	61	49	95	116	26	Pasture
monosaccharide	1.705	2.217	0.765	2.900	0.003733862	0.027102	Carbohydrates	Monosaccharides	D-Galacturonate_and_D-Glucuronate_Utilization	Alpha-1,4-digalacturonate ABC transporter, substrate-binding protein	0	0	0	0	1	1	2	3	2	9	Pasture
monosaccharide	230.025	1.061	0.372	2.850	0.00437438	0.030492	Carbohydrates	Monosaccharides	D-gluconate_and_ketogluconates_metabolism	Broad-specificity glycerol dehydrogenase (EC 1.1.99.22), subunit SldA	53	132	159	170	177	308	262	406	452	295	Pasture
monosaccharide	186.933	1.223	0.436	2.805	0.005030477	0.03369	Carbohydrates	Monosaccharides	L-fucose_utilization_temp	L-fuconate dehydratase (EC 4.2.1.68)	96	127	121	88	56	124	231	467	186	421	Pasture
monosaccharide	6.520	1.271	0.454	2.798	0.005149288	0.034321	Carbohydrates	Monosaccharides	L-ascorbate_utilization_(and_related_gene_clusters)	Ascorbate-specific PTS system, EIIC component	1	4	3	7	2	8	17	9	8	10	Pasture
monosaccharide	21.674	1.111	0.398	2.793	0.005222259	0.034699	Carbohydrates	Monosaccharides	L-fucose_utilization	Lactaldehyde dehydrogenase involved in fucose or rhamnose utilization (EC 1.2.1.22)	4	16	9	28	6	32	43	36	46	10	Pasture
monosaccharide	75.034	1.242	0.446	2.784	0.005375687	0.035407	Carbohydrates	Monosaccharides	Mannose_Metabolism	Beta-mannosidase (EC 3.2.1.25)	20	78	26	40	30	113	68	246	97	57	Pasture
monosaccharide	23.877	1.022	0.369	2.767	0.005649582	0.036624	Carbohydrates	Monosaccharides	D-Tagatose_and_Galactitol_Utilization	Galactitol utilization operon repressor	2	20	18	17	16	60	31	41	27	19	Pasture
monosaccharide	17.777	0.948	0.359	2.644	0.008202886	0.047915	Carbohydrates	Monosaccharides	L-Arabinose_utilization	Predicted glycerol-1-phosphate dehydrogenase, arabinose operon	6	18	9	8	14	27	27	10	41	26	Pasture

glycoside hydrolase	4.167	1.891	0.566	3.341	0.00083433	0.008687	Carbohydrates	Glycoside hydrolases	Predicted_carbohydrate_hydrolases	Polysaccharide intercellular adhesin (PIA) biosynthesis deacetylase IcaB (EC 3.-.-.-)	0	0	4	1	2	9	4	9	10	6	Pasture
di- and oligosaccharides	68.006	-1.671	0.443	-3.773	0.000161076	0.002371	Carbohydrates	Di- and oligosaccharides	Trehalose_Uptake_and_Utilization	Maltose-6'-phosphate glucosidase (EC 3.2.1.122)	125	86	49	90	128	63	18	8	30	45	Forest
di- and oligosaccharides	31.911	-1.300	0.351	-3.702	0.00021419	0.002984	Carbohydrates	Di- and oligosaccharides	Trehalose_Uptake_and_Utilization	Trehalose operon transcriptional repressor	39	30	21	64	60	30	17	14	24	13	Forest
di- and oligosaccharides	60.721	-1.169	0.364	-3.215	0.001302245	0.012415	Carbohydrates	Di- and oligosaccharides	Lactose_and_Galactose_Uptake_and_Utilization	Tagatose-6-phosphate kinase (EC 2.7.1.144)	93	66	70	120	43	40	33	22	51	52	Forest
di- and oligosaccharides	110.596	-1.439	0.465	-3.097	0.001953587	0.01681	Carbohydrates	Di- and oligosaccharides	Trehalose_Uptake_and_Utilization	Trehalase (EC 3.2.1.28)	248	42	233	112	118	78	40	34	43	104	Forest
di- and oligosaccharides	601.872	-1.337	0.480	-2.785	0.005355918	0.035349	Carbohydrates	Di- and oligosaccharides	Maltose_and_Maltodextrin_Utilization	Maltose phosphorylase (EC 2.4.1.8)	1148	466	1188	299	922	416	212	556	99	410	Forest
di- and oligosaccharides	94.211	-1.037	0.375	-2.764	0.00571013	0.036907	Carbohydrates	Di- and oligosaccharides	Fructooligosaccharides(FOS)_and_Raffinose_Utilization	Multiple sugar ABC transporter, membrane-spanning permease protein MsmF	100	113	113	158	115	68	35	43	60	118	Forest
di- and oligosaccharides	96.930	-1.388	0.517	-2.686	0.00722565	0.043806	Carbohydrates	Di- and oligosaccharides	Trehalose_Uptake_and_Utilization	Trehalose-6-phosphate hydrolase (EC 3.2.1.93)	298	138	109	45	26	41	82	25	81	41	Forest
di- and oligosaccharides	10.717	1.952	0.409	4.774	1.80E-06	5.84E-05	Carbohydrates	Di- and oligosaccharides	Beta-Glucoside_Metabolism	6-phospho-beta-glucosidase ascB (EC 3.2.1.86)	3	1	5	4	6	16	14	17	24	24	Pasture
di- and oligosaccharides	31.810	1.697	0.371	4.573	4.80E-06	0.000136	Carbohydrates	Di- and oligosaccharides	Trehalose_Uptake_and_Utilization	PTS system, glucose-specific IIB component (EC 2.7.1.69)	9	12	11	26	9	92	42	30	75	35	Pasture
di- and oligosaccharides	42.693	1.513	0.355	4.264	2.01E-05	0.000436	Carbohydrates	Di- and oligosaccharides	Beta-Glucoside_Metabolism	Predicted beta-glucoside transporter, GPH family	17	19	16	31	16	88	85	78	76	26	Pasture
di- and oligosaccharides	18.277	1.334	0.324	4.113	3.90E-05	0.000752	Carbohydrates	Di- and oligosaccharides	Fructooligosaccharides(FOS)_and_Raffinose_Utilization	Two-component sensor kinase YesM (EC 2.7.3.-)	8	6	10	10	13	27	29	33	25	30	Pasture
di- and oligosaccharides	45.405	1.346	0.337	3.993	6.53E-05	0.001167	Carbohydrates	Di- and oligosaccharides	Maltose_and_Maltodextrin_Utilization	Mlc, transcriptional repressor of MalT (the transcriptional activator of maltose regulon) and manXYZ operon	12	23	42	25	16	57	75	96	75	57	Pasture
di- and oligosaccharides	236.008	1.493	0.380	3.924	8.69E-05	0.001453	Carbohydrates	Di- and oligosaccharides	Trehalose_Uptake_and_Utilization	Trehalose synthase, nucleoside diphosphate glucose dependent	63	136	123	71	157	384	575	362	296	321	Pasture
di- and oligosaccharides	6.429	1.559	0.468	3.331	0.00086457	0.008949	Carbohydrates	Di- and oligosaccharides	Maltose_and_Maltodextrin_Utilization	Maltose regulon regulatory protein MalI (repressor for malXY)	2	2	4	3	3	16	6	7	11	14	Pasture
di- and oligosaccharides	11.947	1.211	0.364	3.323	0.000889832	0.009149	Carbohydrates	Di- and oligosaccharides	Maltose_and_Maltodextrin_Utilization	ABC-type sugar transport system, periplasmic binding protein YcjN	2	7	7	11	6	21	13	18	22	19	Pasture
di- and oligosaccharides	13.695	1.120	0.351	3.186	0.001442452	0.013413	Carbohydrates	Di- and oligosaccharides	Trehalose_Uptake_and_Utilization	PTS system, trehalose-specific IIC component (EC 2.7.1.69)	7	6	7	10	9	16	18	31	20	18	Pasture
di- and oligosaccharides	2.111	2.169	0.713	3.043	0.002345603	0.019108	Carbohydrates	Di- and oligosaccharides	Beta-Glucoside_Metabolism	PTS system, diacetylchitobiose-specific IIA component (EC 2.7.1.6	0	1	1	0	0	4	5	3	6	3	Pasture
di- and oligosaccharides	13.154	1.064	0.352	3.025	0.002484154	0.020042	Carbohydrates	Di- and oligosaccharides	Trehalose_Uptake_and_Utilization	PTS system, trehalose-specific IIB component (EC 2.7.1.69)	5	6	8	11	9	14	28	22	21	14	Pasture
di- and oligosaccharides	6.281	1.388	0.467	2.971	0.002971818	0.022983	Carbohydrates	Di- and oligosaccharides	Sucrose_utilization	Sucrose permease, major facilitator superfamily	2	5	3	4	1	7	9	6	14	15	Pasture
di- and oligosaccharides	3.987	1.638	0.555	2.954	0.00313936	0.023978	Carbohydrates	Di- and oligosaccharides	Lactose_and_Galactose_Uptake_and_Utilization	Lactose and galactose permease, GPH translocator family	0	0	2	2	4	7	7	5	9	7	Pasture
di- and oligosaccharides	40.359	1.026	0.350	2.936	0.003322957	0.025013	Carbohydrates	Di- and oligosaccharides	Lactose_utilization	Evolved beta-D-galactosidase, alpha subunit	26	12	36	25	22	32	73	45	96	56	Pasture
di- and oligosaccharides	3.890	1.599	0.557	2.869	0.004123505	0.029251	Carbohydrates	Di- and oligosaccharides	Lactose_utilization	Lactose and galactose permease, GPH translocator family	0	0	2	2	4	7	7	5	9	6	Pasture
di- and oligosaccharides	4.111	2.161	0.763	2.833	0.004610147	0.031719	Carbohydrates	Di- and oligosaccharides	Trehalose_Uptake_and_Utilization	Transcriptional regulator of trehalose utilization, LacI family	0	2	0	0	1	12	0	4	10	15	Pasture
di- and oligosaccharides	2.461	1.973	0.698	2.827	0.00469448	0.032137	Carbohydrates	Di- and oligosaccharides	Lactose_and_Galactose_Uptake_and_Utilization	Galactose permease	0	1	0	2	0	3	7	9	0	4	Pasture
di- and oligosaccharides	9.295	1.086	0.397	2.737	0.006201609	0.039193	Carbohydrates	Di- and oligosaccharides	Unknown_oligosaccharide_utilization_Sde_1396	2nd GPH family transporter in unknown oligosaccharide utilization Sde 1396	2	5	3	9	8	15	10	19	12	14	Pasture
di- and oligosaccharides	86.852	0.957	0.350	2.730	0.006329912	0.039749	Carbohydrates	Di- and oligosaccharides	Unknown_oligosaccharide_utilization_Sde_1396	1st GPH family transporter in unknown oligosaccharide utilization Sde 1396	29	58	59	74	53	114	209	114	125	77	Pasture
di- and oligosaccharides	4.450	1.487	0.545	2.729	0.006348319	0.039826	Carbohydrates	Di- and oligosaccharides	Beta-Glucoside_Metabolism	PTS system, arbutin-, cellobiose-, and salicin-specific IIBC component (EC 2.7.1.69)	0	0	2	5	3	7	6	2	11	12	Pasture
di- and oligosaccharides	23.815	0.932	0.350	2.666	0.007681688	0.045832	Carbohydrates	Di- and oligosaccharides	Maltose_and_Maltodextrin_Utilization	Maltose/maltodextrin ABC transporter 2, permease protein MalF2	15	21	9	16	12	22	40	52	21	35	Pasture
di- and oligosaccharides	9.597	1.011	0.381	2.654	0.007948544	0.046845	Carbohydrates	Di- and oligosaccharides	Beta-Glucoside_Metabolism	Transcriptional antiterminator of lichenan operon, BglG family	3	5	6	7	8	13	13	19	13	13	Pasture
co2 fixation	57.526	-3.921	0.454	-8.632	6.02E-18	1.06E-15	Carbohydrates	CO2 fixation	Calvin-Benson_cycle	Ribose 1,5-bisphosphate or 5-ribose-1,2-cyclic phosphate dehydrogenase	99	142	50	170	40	7	9	6	3	5	Forest
co2 fixation	31.898	-1.691	0.418	-4.044	5.25E-05	0.000969	Carbohydrates	CO2 fixation	Calvin-Benson_cycle	NAD(P)-dependent glyceraldehyde 3-phosphate dehydrogenase archaeal (EC 1.2.1.59)	12	21	59	118	36	16	14	9	16	22	Forest
co2 fixation	179.588	-1.477	0.423	-3.495	0.000474135	0.005632	Carbohydrates	CO2 fixation	Photorespiration_(oxidative_C2_cycle)	Malate synthase G (EC 2.3.3.9)	211	325	412	131	170	89	71	49	135	144	Forest
co2 fixation	495.588	-1.957	0.564	-3.469	0.000522113	0.006072	Carbohydrates	CO2 fixation	Calvin-Benson_cycle	Transketolase, N-terminal section (EC 2.2.1.1)	1301	1129	90	922	121	74	146	266	161	256	Forest
co2 fixation	63.779	-1.470	0.485	-3.028	0.002458115	0.01987	Carbohydrates	CO2 fixation	Calvin-Benson_cycle	Fructose-1,6-bisphosphatase, type V, archaeal (EC 3.1.3.11)	155	28	189	36	29	50	21	32	43	24	Forest
co2 fixation	297.411	-1.290	0.451	-2.864	0.004185004	0.029573	Carbohydrates	CO2 fixation	Photorespiration_(oxidative_C2_cycle)	Serine--glyoxylate aminotransferase (EC 2.6.1.45)	313	329	277	576	528	144	257	289	157	40	Forest
co2 fixation	304.365	-1.310	0.478	-2.738	0.006172125	0.039065	Carbohydrates	CO2 fixation	Photorespiration_(oxidative_C2_cycle)	D-Lactate dehydrogenase, cytochrome c-dependent (EC 1.1.2.4)	854	281	153	179	446	168	188	136	255	149	Forest
co2 fixation	18.490	1.598	0.378	4.229	2.35E-05	0.000501	Carbohydrates	CO2 fixation	CO2_uptake,_carboxysome	Sensory subunit of low CO2-induced protein complex, putative	4	9	10	8	10	24	53	36	33	10	Pasture
co2 fixation	10.112	1.525	0.399	3.821	0.000133168	0.00204	Carbohydrates	CO2 fixation	Photorespiration_(oxidative_C2_cycle)	Ribulose bisphosphate carboxylase (EC 4.1.1.39)	2	7	2	6	6	13	17	18	24	12	Pasture
co2 fixation	11.238	1.530	0.423	3.619	0.00029571	0.003836	Carbohydrates	CO2 fixation	CO2_uptake,_carboxysome	Ribulose bisphosphate carboxylase (EC 4.1.1.39)	3	8	2	6	6	17	19	36	11	9	Pasture
co2 fixation	9.391	1.296	0.408	3.180	0.001474424	0.013641	Carbohydrates	CO2 fixation	Calvin-Benson_cycle	Ribulose bisphosphate carboxylase (EC 4.1.1.39)	3	7	2	6	6	14	13	23	16	8	Pasture
co2 fixation	8.004	1.212	0.436	2.779	0.005453178	0.035712	Carbohydrates	CO2 fixation	Photorespiration_(oxidative_C2_cycle)	Aminomethyl transferase, THF-dependent (glycine cleavage system T protein) (EC 2.1.2.10), mitochondrial	3	7	0	3	8	9	16	17	12	8	Pasture
co2 fixation	8.809	1.070	0.402	2.659	0.007839874	0.046409	Carbohydrates	CO2 fixation	Photorespiration_(oxidative_C2_cycle)	Glutamate/malate translocator DiT2.1, plastidic	0	7	4	7	8	14	17	14	13	9	Pasture
co2 fixation	45.313	1.132	0.431	2.625	0.008653575	0.049776	Carbohydrates	CO2 fixation	Photorespiration_(oxidative_C2_cycle)	Glycerate kinase (EC 2.7.1.31)	5	32	22	31	39	32	45	119	42	101	Pasture
fermentation	136.147	-1.822	0.517	-3.525	0.000423297	0.005113	Carbohydrates	Fermentation	Acetone_Butanol_Ethanol_Synthesis	Electron transfer flavoprotein, beta subunit	114	37	110	477	326	125	41	53	25	42	Forest
fermentation	151.984	-1.533	0.479	-3.198	0.001381686	0.012989	Carbohydrates	Fermentation	Acetyl-CoA_fermentation_to_Butyrate	Butyrate-acetoacetate CoA-transferase subunit A (EC 2.8.3.9)	179	144	207	125	426	130	35	91	120	18	Forest
fermentation	222.858	-1.122	0.412	-2.724	0.006446649	0.040324	Carbohydrates	Fermentation	Acetyl-CoA_fermentation_to_Butyrate	3-hydroxybutyryl-CoA dehydratase (EC 4.2.1.55)	371	125	233	286	407	171	109	91	156	207	Forest
fermentation	26.994	1.908	0.385	4.955	7.23E-07	2.58E-05	Carbohydrates	Fermentation	Acetyl-CoA_fermentation_to_Butyrate	Butyrate kinase (EC 2.7.2.7)	6	6	11	13	14	55	27	80	22	49	Pasture
fermentation	58.615	1.703	0.406	4.198	2.70E-05	0.000564	Carbohydrates	Fermentation	Acetyl-CoA_fermentation_to_Butyrate	Probable electron transfer flavoprotein-quinone oxidoreductase FixC (EC 1.5.5.-)	20	17	25	20	38	120	190	87	35	69	Pasture
fermentation	6.354	1.783	0.496	3.599	0.000319689	0.00411	Carbohydrates	Fermentation	Acetone_Butanol_Ethanol_Synthesis	NADH-dependent butanol dehydrogenase B (EC 1.1.1.-)	1	1	3	3	4	5	6	10	21	14	Pasture
fermentation	5.862	1.649	0.493	3.345	0.000824282	0.008623	Carbohydrates	Fermentation	Butanol_Biosynthesis	NADH-dependent butanol dehydrogenase B (EC 1.1.1.-)	1	1	3	3	4	5	6	14	13	12	Pasture
fermentation	1.805	2.296	0.755	3.040	0.002368473	0.01923	Carbohydrates	Fermentation	Acetyl-CoA_fermentation_to_Butyrate	Ferredoxin-like protein YdiT	1	0	0	0	0	3	1	7	4	3	Pasture
fermentation	17.614	1.073	0.361	2.971	0.002972554	0.022983	Carbohydrates	Fermentation	Acetone_Butanol_Ethanol_Synthesis	NADH-dependent butanol dehydrogenase A (EC 1.1.1.-)	6	9	8	15	14	30	16	13	48	27	Pasture
fermentation	988.039	1.038	0.372	2.792	0.005232554	0.034737	Carbohydrates	Fermentation	Fermentations:_Lactate	Fructose-6-phosphate phosphoketolase (EC 4.1.2.22)	472	678	743	498	525	2772	949	1652	1068	914	Pasture
fermentation	7.760	1.211	0.438	2.763	0.00571855	0.036924	Carbohydrates	Fermentation	Butanol_Biosynthesis	NADPH-dependent butanol dehydrogenase (EC 1.1.1.-)	2	1	2	6	10	10	8	14	19	10	Pasture
fermentation	17.672	0.935	0.348	2.684	0.007278045	0.043977	Carbohydrates	Fermentation	Butanol_Biosynthesis	NADH-dependent butanol dehydrogenase A (EC 1.1.1.-)	5	10	14	12	15	15	19	40	23	30	Pasture
one-carbon	346.208	-1.948	0.465	-4.186	2.84E-05	0.000588	Carbohydrates	One-carbon Metabolism	One-carbon_metabolism_by_tetrahydropterines	N(5),N(10)-methenyltetrahydromethanopterin cyclohydrolase (EC 3.5.4.27)	1072	182	394	295	520	179	101	141	135	138	Forest
one-carbon	7.728	-1.707	0.441	-3.871	0.00010837	0.001726	Carbohydrates	One-carbon Metabolism	Methanogenesis_from_methylated_compounds	Dimethylamine permease	11	7	9	11	18	2	4	7	2	3	Forest
one-carbon	363.833	-1.235	0.357	-3.462	0.000536069	0.006206	Carbohydrates	One-carbon Metabolism	One-carbon_metabolism_by_tetrahydropterines	Methylene tetrahydromethanopterin dehydrogenase (EC 1.5.99.9)	481	356	662	379	524	212	292	136	283	232	Forest
one-carbon	252.812	-1.402	0.413	-3.397	0.000682286	0.007523	Carbohydrates	One-carbon Metabolism	Serine-glyoxylate_cycle	N(5),N(10)-methenyltetrahydromethanopterin cyclohydrolase (EC 3.5.4.27)	196	429	599	374	176	146	156	65	168	186	Forest
one-carbon	199.874	-1.077	0.360	-2.991	0.002776429	0.021917	Carbohydrates	One-carbon Metabolism	Methanogenesis_from_methylated_compounds	Trimethylamine:corrinoid methyltransferase	254	250	231	249	279	121	210	156	98	97	Forest
one-carbon	312.484	-1.478	0.501	-2.952	0.003161282	0.024051	Carbohydrates	One-carbon Metabolism	Methanogenesis	Formylmethanofuran--tetrahydromethanopterin N-formyltransferase (EC 2.3.1.101)	516	136	774	195	584	204	90	183	308	46	Forest
one-carbon	179.298	-1.512	0.551	-2.747	0.006008866	0.038361	Carbohydrates	One-carbon Metabolism	Serine-glyoxylate_cycle	Formate--tetrahydrofolate ligase (EC 6.3.4.3)	113	65	62	299	781	93	95	61	179	30	Forest
one-carbon	76.333	-1.072	0.391	-2.744	0.006076155	0.038648	Carbohydrates	One-carbon Metabolism	Methanogenesis	Formylmethanofuran dehydrogenase subunit B (EC 1.2.99.5)	43	129	127	98	100	34	68	25	45	87	Forest
one-carbon	245.885	-1.445	0.540	-2.679	0.007394854	0.044578	Carbohydrates	One-carbon Metabolism	Serine-glyoxylate_cycle	Methylmalonyl-CoA mutase, small subunit (EC 5.4.99.2)	792	200	337	222	55	247	78	63	93	163	Forest
one-carbon	902.528	-0.874	0.331	-2.639	0.008324542	0.048427	Carbohydrates	One-carbon Metabolism	Serine-glyoxylate_cycle	Fumarate hydratase class I, N-terminal domain	1016	880	955	1002	1601	469	856	811	710	567	Forest
one-carbon	7.543	3.875	0.620	6.250	4.11E-10	2.73E-08	Carbohydrates	One-carbon Metabolism	Methanogenesis_from_methylated_compounds	Methylcobalamin:coenzyme M methyltransferase, methylamine-specific	1	0	0	1	0	14	16	14	19	17	Pasture
one-carbon	27.490	2.198	0.372	5.907	3.49E-09	1.99E-07	Carbohydrates	One-carbon Metabolism	Methanogenesis_from_methylated_compounds	Trimethylamine methyltransferase corrinoid protein	6	11	5	4	16	31	69	57	58	36	Pasture
one-carbon	5.006	4.081	0.720	5.668	1.44E-08	7.36E-07	Carbohydrates	One-carbon Metabolism	Methanogenesis	Formylmethanofuran dehydrogenase (tungsten) operon gene F (polyferredoxin)	0	0	0	0	0	8	5	13	12	16	Pasture
one-carbon	60.194	1.933	0.371	5.217	1.82E-07	7.36E-06	Carbohydrates	One-carbon Metabolism	Methanogenesis_from_methylated_compounds	Methylthiol:coenzyme M methyltransferase	9	22	44	21	16	87	133	139	84	85	Pasture
one-carbon	3.262	3.558	0.746	4.771	1.83E-06	5.92E-05	Carbohydrates	One-carbon Metabolism	Methanogenesis	N5-methyltetrahydromethanopterin:coenzyme M methyltransferase subunit X	0	0	0	0	0	4	3	7	7	14	Pasture
one-carbon	34.675	1.648	0.352	4.678	2.90E-06	8.75E-05	Carbohydrates	One-carbon Metabolism	Serine-glyoxylate_cycle	Serine-pyruvate aminotransferase/archaeal aspartate aminotransferase	6	17	21	20	12	68	72	37	35	79	Pasture
one-carbon	27.792	1.476	0.342	4.321	1.55E-05	0.000354	Carbohydrates	One-carbon Metabolism	Methanogenesis	CoB--CoM heterodisulfide reductase subunit B (EC 1.8.98.1)	6	12	15	20	14	38	40	47	29	70	Pasture
one-carbon	4.511	2.781	0.649	4.284	1.84E-05	0.000406	Carbohydrates	One-carbon Metabolism	Methanogenesis_from_methylated_compounds	Dimethylamine:corrinoid methyltransferase	1	1	0	0	1	6	1	14	5	18	Pasture
one-carbon	3.153	3.001	0.719	4.174	2.99E-05	0.000613	Carbohydrates	One-carbon Metabolism	Methanogenesis	Methyl coenzyme M reductase gamma subunit (EC 2.8.4.1)	1	0	0	0	0	2	4	9	3	14	Pasture
one-carbon	4.819	2.606	0.632	4.125	3.71E-05	0.000721	Carbohydrates	One-carbon Metabolism	Methanogenesis	Methyl coenzyme M reductase beta subunit (EC 2.8.4.1)	1	1	0	1	1	6	4	14	1	21	Pasture
one-carbon	2.466	3.161	0.767	4.123	3.75E-05	0.000727	Carbohydrates	One-carbon Metabolism	Methanogenesis	Formylmethanofuran dehydrogenase (molybdenum) operon gene E	0	0	0	0	0	5	1	9	1	10	Pasture
one-carbon	5.239	2.186	0.547	3.995	6.47E-05	0.001161	Carbohydrates	One-carbon Metabolism	Methanogenesis_from_methylated_compounds	pyrrolysine-containing	2	1	0	2	2	10	8	9	5	16	Pasture
one-carbon	3.690	2.578	0.648	3.978	6.96E-05	0.001226	Carbohydrates	One-carbon Metabolism	Methanogenesis	Methyl coenzyme M reductase operon protein D	0	1	0	0	2	8	4	11	3	10	Pasture
one-carbon	23.659	1.308	0.335	3.903	9.52E-05	0.001555	Carbohydrates	One-carbon Metabolism	Methanogenesis_from_methylated_compounds	Dimethylamine methyltransferase corrinoid protein	9	17	10	13	12	33	30	58	28	35	Pasture
one-carbon	3.252	3.035	0.784	3.871	0.000108322	0.001726	Carbohydrates	One-carbon Metabolism	Methanogenesis	N5-methyltetrahydromethanopterin:coenzyme M methyltransferase subunit D (EC 2.1.1.86)	0	0	0	0	0	0	5	7	9	14	Pasture
one-carbon	3.230	2.672	0.695	3.845	0.000120327	0.001877	Carbohydrates	One-carbon Metabolism	Methanogenesis_from_methylated_compounds	Monomethylamine:corrinoid methyltransferase	0	0	0	1	1	1	4	11	3	13	Pasture
one-carbon	3.891	2.374	0.626	3.793	0.000148552	0.002227	Carbohydrates	One-carbon Metabolism	Methanogenesis	Methyl coenzyme M reductase alpha subunit (EC 2.8.4.1)	0	2	0	1	1	2	7	12	5	11	Pasture
one-carbon	5.880	2.552	0.698	3.658	0.000254136	0.003407	Carbohydrates	One-carbon Metabolism	Methanogenesis	Methyl coenzyme M reductase associated protein	2	2	0	0	0	8	4	26	5	14	Pasture
one-carbon	3.367	2.142	0.628	3.409	0.000651183	0.007276	Carbohydrates	One-carbon Metabolism	Methanogenesis	N5-methyltetrahydromethanopterin:coenzyme M methyltransferase subunit E (EC 2.1.1.86)	2	2	0	0	0	5	5	8	5	8	Pasture
one-carbon	1.616	2.653	0.787	3.370	0.000751908	0.008119	Carbohydrates	One-carbon Metabolism	Methanogenesis	Methyl coenzyme M reductase operon protein C	0	0	0	0	0	2	1	8	1	5	Pasture
one-carbon	6.404	1.678	0.512	3.278	0.001047036	0.010376	Carbohydrates	One-carbon Metabolism	Methanogenesis	Formylmethanofuran dehydrogenase subunit D (EC 1.2.99.5)	6	3	2	1	0	11	2	17	15	9	Pasture
one-carbon	1.677	2.263	0.758	2.985	0.002838105	0.022271	Carbohydrates	One-carbon Metabolism	Formaldehyde_assimilation:_Ribulose_monophosphate_pathway	related to 6-phospho-3-hexuloisomerase	0	0	0	1	0	3	3	4	2	5	Pasture
one-carbon	18.344	1.081	0.362	2.984	0.002847661	0.022319	Carbohydrates	One-carbon Metabolism	Formaldehyde_assimilation:_Ribulose_monophosphate_pathway	Transcriptional regulator HxlR, formaldehyde assimilation	5	9	14	12	14	20	14	45	22	35	Pasture
one-carbon	1.589	2.169	0.766	2.832	0.004619039	0.031763	Carbohydrates	One-carbon Metabolism	Formaldehyde_assimilation:_Ribulose_monophosphate_pathway	Putative pentose isomerase	0	0	1	0	0	3	2	7	2	2	Pasture
one-carbon	6.809	1.284	0.464	2.769	0.005615364	0.036478	Carbohydrates	One-carbon Metabolism	Serine-glyoxylate_cycle	hydroxypyruvate reductase	3	5	4	1	4	5	9	20	8	11	Pasture
one-carbon	3.259	1.635	0.608	2.688	0.007185794	0.043647	Carbohydrates	One-carbon Metabolism	Methanogenesis	F420-dependent methylenetetrahydromethanopterin dehydrogenase (EC 1.5.99.9)	2	0	2	1	1	2	5	5	5	11	Pasture
one-carbon	3.259	1.635	0.608	2.688	0.007185794	0.043647	Carbohydrates	One-carbon Metabolism	One-carbon_metabolism_by_tetrahydropterines	F420-dependent methylenetetrahydromethanopterin dehydrogenase (EC 1.5.99.9)	2	0	2	1	1	2	5	5	5	11	Pasture
one-carbon	3.460	1.574	0.587	2.679	0.007376787	0.044511	Carbohydrates	One-carbon Metabolism	Methanogenesis_from_methylated_compounds	Methanol methyltransferase corrinoid protein	0	1	0	4	2	4	5	4	8	9	Pasture
central carbon	102.809	-3.844	0.421	-9.140	6.25E-20	1.40E-17	Carbohydrates	Central carbohydrate metabolism	Glycolate,_glyoxylate_interconversions	Phosphoglycolate phosphatase, archaeal type (EC 3.1.3.18)	90	167	173	352	150	8	9	9	11	22	Forest
central carbon	294.799	-3.005	0.407	-7.383	1.55E-13	1.81E-11	Carbohydrates	Central carbohydrate metabolism	Pyruvate_metabolism_II:_acetyl-CoA,_acetogenesis_from_pyruvate	Acetyl-CoA synthetase (ADP-forming) alpha chain (EC 6.2.1.13)	300	515	588	660	456	50	99	23	52	87	Forest
central carbon	160.727	-2.608	0.447	-5.834	5.40E-09	2.96E-07	Carbohydrates	Central carbohydrate metabolism	Pyruvate:ferredoxin_oxidoreductase	Pyruvate:ferredoxin oxidoreductase, delta subunit (EC 1.2.7.1)	372	92	448	126	254	46	30	40	39	57	Forest
central carbon	33.716	-1.798	0.373	-4.824	1.41E-06	4.72E-05	Carbohydrates	Central carbohydrate metabolism	Particulate_methane_monooxygenase_(pMMO)	Particulate methane monooxygenase B-subunit (EC 1.14.13.25)	62	32	71	34	45	13	11	7	17	29	Forest
central carbon	244.115	-1.703	0.399	-4.270	1.96E-05	0.000426	Carbohydrates	Central carbohydrate metabolism	Glycolysis_and_Gluconeogenesis	Polyphosphate glucokinase (EC 2.7.1.63)	164	388	480	410	370	64	183	123	155	67	Forest
central carbon	83.942	-1.639	0.417	-3.930	8.48E-05	0.001427	Carbohydrates	Central carbohydrate metabolism	Particulate_methane_monooxygenase_(pMMO)	Particulate methane monooxygenase C-subunit (EC 1.14.13.25)	143	77	204	46	127	55	34	25	32	61	Forest
central carbon	209.958	-2.023	0.558	-3.629	0.000284579	0.003714	Carbohydrates	Central carbohydrate metabolism	Dehydrogenase_complexes	Cytosol aminopeptidase PepA (EC 3.4.11.1)	381	82	987	111	101	154	59	52	28	80	Forest
central carbon	577.898	-1.879	0.528	-3.557	0.000375037	0.00464	Carbohydrates	Central carbohydrate metabolism	Entner-Doudoroff_Pathway	Glucose dehydrogenase, PQQ-dependent (EC 1.1.5.2)	673	283	1880	1536	199	255	129	151	94	510	Forest
central carbon	99.985	-1.673	0.480	-3.486	0.000490975	0.005794	Carbohydrates	Central carbohydrate metabolism	Glycolysis_and_Gluconeogenesis,_including_Archaeal_enzymes	Fructose-1,6-bisphosphatase, type V, archaeal (EC 3.1.3.11)	115	71	47	442	74	54	47	29	53	54	Forest
central carbon	20.677	-1.075	0.309	-3.479	0.000502597	0.005898	Carbohydrates	Central carbohydrate metabolism	Particulate_methane_monooxygenase_(pMMO)	Particulate methane monooxygenase A-subunit (EC 1.14.13.25)	23	25	38	26	20	12	15	15	13	16	Forest
central carbon	24.728	-1.389	0.423	-3.280	0.001038481	0.010324	Carbohydrates	Central carbohydrate metabolism	Glycolysis_and_Gluconeogenesis,_including_Archaeal_enzymes	NAD(P)-dependent glyceraldehyde 3-phosphate dehydrogenase archaeal (EC 1.2.1.59)	9	11	28	39	90	19	12	11	16	13	Forest
central carbon	91.001	-1.199	0.369	-3.253	0.001141365	0.011164	Carbohydrates	Central carbohydrate metabolism	TCA_Cycle	Aconitate hydratase large subunit (EC 4.2.1.3)	177	56	127	106	117	60	66	60	55	50	Forest
central carbon	147.997	-1.353	0.416	-3.252	0.001146584	0.011181	Carbohydrates	Central carbohydrate metabolism	Pyruvate_metabolism_II:_acetyl-CoA,_acetogenesis_from_pyruvate	Pyruvate oxidase [ubiquinone, cytochrome] (EC 1.2.2.2)	152	77	397	230	185	64	142	60	68	98	Forest
central carbon	147.333	-1.387	0.435	-3.191	0.001419568	0.013267	Carbohydrates	Central carbohydrate metabolism	Entner-Doudoroff_Pathway	D-glycerate 2-kinase (EC 2.7.1.-)	294	83	205	198	211	130	35	51	110	94	Forest
central carbon	47.429	-1.180	0.372	-3.176	0.001491999	0.013773	Carbohydrates	Central carbohydrate metabolism	TCA_Cycle	Archaeal succinyl-CoA ligase [ADP-forming] alpha chain (EC 6.2.1.5)	28	61	118	58	55	13	32	39	28	40	Forest
central carbon	204.707	-1.483	0.506	-2.932	0.003366284	0.025223	Carbohydrates	Central carbohydrate metabolism	Pyruvate_metabolism_I:_anaplerotic_reactions,_PEP	NAD-dependent malic enzyme (EC 1.1.1.38)	545	377	76	263	77	123	156	91	84	82	Forest
central carbon	244.980	-1.675	0.576	-2.909	0.003629857	0.026498	Carbohydrates	Central carbohydrate metabolism	Entner-Doudoroff_Pathway	2,3-bisphosphoglycerate-independent phosphoglycerate mutase (EC 5.4.2.1)	227	102	121	91	1286	166	41	54	131	150	Forest
central carbon	407.852	-1.363	0.482	-2.829	0.00467096	0.032017	Carbohydrates	Central carbohydrate metabolism	Dehydrogenase_complexes	Acetoin dehydrogenase E1 component beta-subunit (EC 1.2.4.-)	178	921	264	270	1172	152	203	221	367	217	Forest
central carbon	108.989	-1.363	0.486	-2.804	0.005042369	0.033732	Carbohydrates	Central carbohydrate metabolism	Glycolysis_and_Gluconeogenesis,_including_Archaeal_enzymes	Phosphoglycerate mutase (EC 5.4.2.1)	121	75	161	87	312	49	17	71	146	29	Forest
central carbon	84.044	-1.235	0.450	-2.744	0.006069983	0.038628	Carbohydrates	Central carbohydrate metabolism	Glycolate,_glyoxylate_interconversions	Phosphoglycolate phosphatase (EC 3.1.3.18)	35	90	69	169	216	47	31	100	52	26	Forest
central carbon	121.603	-1.567	0.575	-2.724	0.006457441	0.040372	Carbohydrates	Central carbohydrate metabolism	Methylglyoxal_Metabolism	Aldehyde dehydrogenase A (EC 1.2.1.22)	27	538	41	32	221	50	51	60	96	34	Forest
central carbon	340.837	-1.342	0.493	-2.723	0.006472224	0.040444	Carbohydrates	Central carbohydrate metabolism	Entner-Doudoroff_Pathway	Glucose-6-phosphate 1-dehydrogenase (EC 1.1.1.49)	261	520	119	304	1128	136	368	132	168	174	Forest
central carbon	146.340	-1.327	0.493	-2.693	0.007082062	0.043194	Carbohydrates	Central carbohydrate metabolism	Pyruvate_metabolism_II:_acetyl-CoA,_acetogenesis_from_pyruvate	NAD-dependent protein deacetylase of SIR2 family	257	141	74	172	328	201	80	71	29	41	Forest
central carbon	902.528	-0.874	0.331	-2.639	0.008324542	0.048427	Carbohydrates	Central carbohydrate metabolism	TCA_Cycle	Fumarate hydratase class I, N-terminal domain	1016	880	955	1002	1601	469	856	811	710	567	Forest
central carbon	35.573	5.845	0.625	9.349	8.85E-21	2.46E-18	Carbohydrates	Central carbohydrate metabolism	Glycolysis_and_Gluconeogenesis	Predicted Fructose-bisphosphate aldolase (EC 4.1.2.13) in Geobacter	1	0	0	0	0	44	142	96	27	75	Pasture
central carbon	52.501	4.147	0.451	9.200	3.59E-20	8.35E-18	Carbohydrates	Central carbohydrate metabolism	Entner-Doudoroff_Pathway	Glyceraldehyde-3-phosphate: ferredoxin oxidoreductase (EC 1.2.7.6)	3	3	3	8	3	153	89	174	78	53	Pasture
central carbon	49.596	4.076	0.448	9.101	8.92E-20	1.90E-17	Carbohydrates	Central carbohydrate metabolism	Glycolysis_and_Gluconeogenesis,_including_Archaeal_enzymes	Glyceraldehyde-3-phosphate: ferredoxin oxidoreductase (EC 1.2.7.6)	3	3	3	8	3	150	118	136	74	41	Pasture
central carbon	14.801	1.733	0.384	4.509	6.52E-06	0.000174	Carbohydrates	Central carbohydrate metabolism	HPr_kinase_and_hprK_operon_in_Gram-positive_organisms	Nucleoside binding-domain containing protein YvoD	6	2	7	5	10	29	15	38	28	16	Pasture
central carbon	123.853	1.535	0.356	4.313	1.61E-05	0.000363	Carbohydrates	Central carbohydrate metabolism	Glycolysis_and_Gluconeogenesis	6-phosphofructokinase, eukaryotic type (EC 2.7.1.11)	67	51	55	42	62	138	183	198	292	211	Pasture
central carbon	16.297	1.516	0.363	4.176	2.97E-05	0.000611	Carbohydrates	Central carbohydrate metabolism	HPr_kinase_and_hprK_operon_in_Gram-positive_organisms	Pyrophosphatase PpaX (EC 3.6.1.1)	4	5	6	12	11	17	23	41	25	27	Pasture
central carbon	27.457	1.507	0.361	4.169	3.06E-05	0.000624	Carbohydrates	Central carbohydrate metabolism	Pyruvate_metabolism_I:_anaplerotic_reactions,_PEP	Positive regulator of Tartrate dehydrogenase/decarboxylase/D-malic enzyme	6	12	13	22	12	29	57	42	79	22	Pasture
central carbon	83.037	1.582	0.407	3.890	0.000100306	0.001615	Carbohydrates	Central carbohydrate metabolism	Pyruvate_metabolism_II:_acetyl-CoA,_acetogenesis_from_pyruvate	Pyruvate oxidase (EC 1.2.3.3)	18	38	26	42	60	71	244	158	150	75	Pasture
central carbon	775.850	1.268	0.392	3.238	0.001204696	0.011598	Carbohydrates	Central carbohydrate metabolism	Glycolysis_and_Gluconeogenesis	Putative phosphoenolpyruvate synthase/pyruvate phosphate dikinase, C-terminal domain	610	178	280	468	434	864	1246	1449	1491	1040	Pasture
central carbon	16.935	1.136	0.351	3.237	0.001209836	0.011636	Carbohydrates	Central carbohydrate metabolism	Pyruvate_metabolism_II:_acetyl-CoA,_acetogenesis_from_pyruvate	Acetyltransferase AcuA, acetyl-CoA synthetase inhibitor	14	5	7	11	10	21	20	39	24	23	Pasture
central carbon	47.767	1.557	0.482	3.232	0.001228553	0.011792	Carbohydrates	Central carbohydrate metabolism	Pyruvate_metabolism_I:_anaplerotic_reactions,_PEP	Oxaloacetate decarboxylase beta chain (EC 4.1.1.3)	9	27	8	36	24	178	48	49	15	108	Pasture
central carbon	51.178	1.112	0.357	3.112	0.001857	0.016142	Carbohydrates	Central carbohydrate metabolism	Glycolysis_and_Gluconeogenesis,_including_Archaeal_enzymes	Non-phosphorylating glyceraldehyde-3-phosphate dehydrogenase (NAD)	30	19	12	38	46	70	75	96	80	65	Pasture
central carbon	19.861	1.125	0.386	2.918	0.003526749	0.026057	Carbohydrates	Central carbohydrate metabolism	Pyruvate_metabolism_I:_anaplerotic_reactions,_PEP	Two-component response regulator, malate (EC 2.7.3.-)	9	6	17	14	11	51	10	27	49	16	Pasture
central carbon	44.518	1.008	0.371	2.720	0.006534571	0.040734	Carbohydrates	Central carbohydrate metabolism	Peripheral_Glucose_Catabolism_Pathways	Glucose ABC transporter, ATP-binding subunit (EC 3.6.3.-)	10	28	46	19	34	62	77	99	58	33	Pasture
central carbon	53.732	1.108	0.410	2.701	0.006913713	0.042457	Carbohydrates	Central carbohydrate metabolism	Pyruvate_Alanine_Serine_Interconversions	Alanine racemase, catabolic (EC 5.1.1.1)	7	29	25	45	51	108	89	28	117	73	Pasture
central carbon	72.664	0.986	0.368	2.678	0.007416768	0.044647	Carbohydrates	Central carbohydrate metabolism	Dihydroxyacetone_kinases	DHA-specific phosphocarrier protein HPr	27	30	47	57	64	152	100	73	60	147	Pasture
central carbon	29.522	0.950	0.359	2.647	0.008129231	0.047572	Carbohydrates	Central carbohydrate metabolism	Glycolysis_and_Gluconeogenesis	Non-phosphorylating glyceraldehyde-3-phosphate dehydrogenase (NADP) (EC 1.2.1.9)	16	19	11	24	21	27	81	43	46	20	Pasture
