# Supplementary material for: New Biological Insights Into How Deforestation in Amazonia Affects Soil Microbial Communities Using Metagenomics and Metagenome-Assembled Genomes
Source: Front Microbiol. 2018 Jul 23;9:1635. doi: 10.3389/fmicb.2018.01635 (PMC6064768; doi:10.3389/fmicb.2018.01635)
Supplement: Supplementary file 12 [file Table_6.PDF]

Supplemental Table 6: The relative abundance of bacterial and archaeal phyla in rainforest (FII) and cattle pasture (P72I) soil samples across 3 (a, b, c) 100 m transects based on iTAG sequencing. Some samples are missing due strict standards in the quality control of the sequencing process.

Taxon	FII_A001a	FII_A001b	FII_A001c	FII_A01a	FII_A01b	FII_A01c	FII_A1a	FII_A1b	FII_A1c	FII_A10a	FII_A10b	FII_A10c	FII_A100a	FII_A100b	FII_A100c	P72I_A001b	P72I_A001c	P72I_A10a	P72I_A10c	P72I_A100a	P72I_A100b
k__Archaea;Other	8.08E-06	7.17E-06	1.65E-05	8.09E-06	1.36E-05	1.33E-05	2.24E-05	1.67E-05	4.34E-05	9.74E-06	1.43E-06	9.18E-06	1.47E-06	6.90E-06	1.64E-06	0	0	0	2.23E-06	0	3.95E-06
k__Archaea;p__ArchaeaKI	4.04E-06	7.17E-06	3.66E-06	5.78E-06	8.52E-06	1.49E-05	8.00E-06	3.33E-06	1.97E-06	3.25E-06	1.29E-05	1.84E-05	3.52E-05	1.38E-05	2.79E-05	0	0	0	0	0	0
k__Archaea;p__Crenarchaeota	0.03551649	0.02292713	0.02879384	0.02950134	0.02578108	0.02961387	0.03673703	0.03308374	0.0316615	0.02849154	0.02761803	0.02550259	0.01807992	0.016468	0.01842536	1.69E-05	1.69E-05	2.73E-05	2.23E-05	0	3.56E-05
k__Archaea;p__DHVE3	6.06E-06	5.73E-06	7.32E-06	1.39E-05	1.02E-05	9.95E-06	1.76E-05	1.17E-05	1.38E-05	0	1.43E-06	0	0	2.76E-06	0	0	0	0	0	0	0
k__Archaea;p__Euryarchaeota	0.00017767	0.00019347	0.00015561	0.00015494	0.0001601	0.00021561	0.00013276	0.00010157	0.00013028	0.00017862	0.00013305	0.00013154	0.00042343	0.00040556	0.00038759	1.21E-05	4.23E-05	5.47E-06	2.90E-05	0	1.98E-05
k__Archaea;p__HydGC-84-221A	2.02E-06	1.43E-06	0	2.31E-06	3.41E-06	1.66E-06	4.80E-06	0	1.97E-06	0	4.29E-06	3.06E-06	1.47E-06	0	0	0	0	0	0	0	0
k__Bacteria;Other	0.00317995	0.00315431	0.00332279	0.00329874	0.0034796	0.00344652	0.00345984	0.00350835	0.00363791	0.00374778	0.00356509	0.00366464	0.00472657	0.00494813	0.00504854	0.00214783	0.00242397	0.00245957	0.00253083	0.00272505	0.0023956
k__Bacteria;p__49S1_2B	2.02E-06	2.87E-06	0	1.16E-06	0	3.32E-06	0	0	1.97E-06	0	0	0	0	0	0	0	0	0	0	0	0
k__Bacteria;p__ABY1_OD1	6.06E-06	8.60E-06	1.28E-05	1.97E-05	2.04E-05	1.99E-05	2.24E-05	3.16E-05	9.87E-06	6.50E-06	5.72E-06	1.22E-05	4.40E-05	3.45E-05	2.46E-05	2.41E-06	0	1.09E-05	6.68E-06	0	3.95E-06
k__Bacteria;p__AD3	0.00107614	0.00199491	0.00085495	0.00084752	0.00094016	0.00073475	0.00137722	0.00158517	0.00121198	0.00135751	0.00150072	0.00149889	0.0026358	0.00257545	0.00262445	0.00121147	0.00109988	0.00043726	0.00038319	0.00117459	0.00088155
k__Bacteria;p__Acidobacteria	0.1472286	0.12544713	0.14606904	0.16113524	0.14561703	0.15591606	0.15724607	0.14971352	0.17210709	0.20614388	0.20832189	0.20856327	0.24665324	0.24427351	0.23681492	0.10093853	0.10500912	0.09738794	0.10632596	0.11017666	0.0979507
k__Bacteria;p__Actinobacteria	0.09146138	0.1515773	0.09341887	0.0695672	0.07838048	0.07200208	0.0643054	0.07456454	0.06584368	0.05917523	0.06076109	0.05903802	0.06358009	0.06955241	0.06637827	0.07658596	0.05886484	0.06141267	0.05463335	0.08823529	0.10596765
k__Bacteria;p__Armatimonadetes	0.00168184	0.00148042	0.00172639	0.0017332	0.00171681	0.0017896	0.00192747	0.00155853	0.00153768	0.00152964	0.00153934	0.00173749	0.00214791	0.00211471	0.00220073	0.00102806	0.00130294	0.00201685	0.00217437	0.00352377	0.00209516
k__Bacteria;p__BRC1	0.00014739	0.00010319	0.0001666	0.000185	0.00023845	0.00030186	0.00016475	0.00020314	0.00017765	0.00027605	0.00025608	0.00022636	0.00094649	0.00103873	0.00100839	0.00013032	0.00010576	0.00016944	0.00025397	0.00023492	0.00022138
k__Bacteria;p__BacteriaKI	5.86E-05	2.29E-05	4.21E-05	2.89E-05	5.62E-05	6.14E-05	4.80E-06	1.67E-06	9.87E-06	6.50E-06	1.29E-05	1.53E-05	1.47E-06	4.14E-06	8.21E-06	0	0	0	0	0	1.19E-05
k__Bacteria;p__Bacteroidetes	0.01902114	0.01552647	0.02269748	0.02083998	0.02875484	0.02598656	0.01750555	0.02188598	0.01913112	0.02189883	0.01886981	0.01794389	0.00476613	0.00608204	0.00708011	0.00695029	0.00923055	0.02510399	0.0228933	0.03260665	0.0246201
k__Bacteria;p__CCM11b	0.00209574	0.00171115	0.00193326	0.00215522	0.00187861	0.00208815	0.00169713	0.00180496	0.00165611	0.00104574	0.00098999	0.0010064	0.00195304	0.00201263	0.00200858	0.00038613	0.00057955	0.0003826	0.00036314	0.00061079	0.00044275
k__Bacteria;p__Chlamydiae	0.0083244	0.00626562	0.0089816	0.01054949	0.00751615	0.00821327	0.00883116	0.00832047	0.00897931	0.00722924	0.00724893	0.00799307	0.01050218	0.01072117	0.00978503	0.00149142	0.00263549	0.00385332	0.00386085	0.00117459	0.00115036
k__Bacteria;p__Chlorobi	0.00051485	0.00036545	0.00059499	0.00074462	0.00079879	0.00096861	0.00055025	0.00050286	0.00065929	0.00056834	0.0005093	0.00050167	0.00015531	0.00020002	0.00023978	6.52E-05	7.19E-05	0.0001913	0.00019828	0.00084571	0.00063645
k__Bacteria;p__Chloroflexi	0.02014169	0.02373971	0.0188749	0.01898885	0.02079757	0.02223652	0.0180542	0.01947826	0.01977857	0.02605906	0.02772103	0.02580543	0.03641478	0.03619981	0.03457937	0.02942532	0.03182466	0.03273411	0.03239281	0.02692163	0.02387296
k__Bacteria;p__Cyanobacteria	0.00086818	0.00085987	0.0011314	0.00099205	0.00113262	0.00136335	0.00151638	0.00157684	0.00277137	0.00187714	0.00334335	0.00190268	0.00455661	0.00442669	0.00429635	0.00269324	0.00287661	0.00365656	0.00367817	0.00159744	0.00169194
k__Bacteria;p__Deferribacteres	0.00012518	0.00014045	0.00011717	0.00016303	0.00013966	0.00014098	0.00025433	0.00025476	0.00022897	0.00013315	0.00011588	0.00014989	9.08E-05	9.38E-05	7.39E-05	9.89E-05	7.61E-05	9.29E-05	8.47E-05	4.70E-05	1.19E-05
k__Bacteria;p__Elusimicrobia	0.00169193	0.00135287	0.00216027	0.00168232	0.00202338	0.00241654	0.00185869	0.00179331	0.00234106	0.00211421	0.00184406	0.00209233	0.00399399	0.00399492	0.00394982	0.00037406	0.00053725	0.00104395	0.00112952	0.00075174	0.00072738
k__Bacteria;p__Firmicutes	0.03765867	0.03274117	0.03415973	0.04888793	0.03660481	0.04680998	0.0404544	0.03981571	0.04447414	0.03784474	0.04917597	0.04400626	0.05314824	0.05797735	0.05353028	0.13367007	0.19669274	0.10611121	0.13913092	0.11821086	0.14733717
k__Bacteria;p__Fusobacteria	0	0	3.66E-06	3.47E-06	0	0	4.80E-06	1.67E-06	0	9.74E-06	4.29E-06	1.22E-05	2.93E-06	4.14E-06	0	2.41E-06	0	0	0	0	7.91E-06
k__Bacteria;p__GAL15	0.00023622	0.00049873	0.00025081	0.00025437	0.0003185	0.00029025	0.00060463	0.00050286	0.00046782	0.0009613	0.00131617	0.00115017	0.00128347	0.00125393	0.0010708	0	4.23E-06	5.47E-06	1.78E-05	0	0
k__Bacteria;p__GN02	1.01E-05	5.73E-06	9.15E-06	9.25E-06	6.81E-06	1.33E-05	4.80E-06	1.67E-06	9.87E-06	1.30E-05	1.86E-05	0	1.61E-05	9.66E-06	1.15E-05	1.21E-05	4.23E-06	3.28E-05	1.11E-05	0	0
k__Bacteria;p__GN04	8.08E-06	5.73E-06	3.66E-06	2.31E-06	1.70E-06	3.32E-06	0	0	0	0	0	3.06E-06	0	0	0	0	0	0	0	0	0
k__Bacteria;p__GN12	2.02E-06	4.30E-06	1.10E-05	1.16E-06	3.41E-06	1.66E-06	0	1.67E-06	0	0	0	0	0	0	0	0	0	0	0	0	0
k__Bacteria;p__GOUTA4	8.08E-06	1.00E-05	1.28E-05	6.94E-06	1.02E-05	1.33E-05	8.00E-06	1.67E-06	1.97E-06	0	2.86E-06	0	1.47E-06	0	0	0	0	0	0	0	0
k__Bacteria;p__Gemmatimonadete	0.02213244	0.02300022	0.02401927	0.01771005	0.01948612	0.01964914	0.01185272	0.01371371	0.01152763	0.00862573	0.00822604	0.00835709	0.00714113	0.00788086	0.0086896	0.00068538	0.00071069	0.00084718	0.00089114	0.00249013	0.00339574
k__Bacteria;p__Lentisphaerae	0	0	1.83E-06	0	1.70E-06	0	1.60E-06	3.33E-06	1.97E-06	3.25E-06	2.86E-06	6.12E-06	4.40E-06	5.52E-06	0	0	0	0	2.23E-06	0	0
k__Bacteria;p__MD2896-B26	1.01E-05	8.60E-06	3.66E-06	1.62E-05	1.02E-05	2.16E-05	6.40E-06	1.33E-05	1.78E-05	3.25E-06	1.29E-05	3.06E-06	2.93E-06	4.14E-06	0	0	0	0	0	0	0
k__Bacteria;p__NC10	0	0	0	1.62E-05	0	1.66E-06	0	0	0	0	0	0	1.03E-05	4.14E-06	1.48E-05	4.83E-06	4.23E-06	0	1.56E-05	0	1.58E-05
k__Bacteria;p__NKB19	2.02E-05	1.00E-05	2.01E-05	1.27E-05	1.53E-05	4.48E-05	1.60E-06	6.66E-06	5.92E-06	1.30E-05	2.00E-05	6.12E-06	4.40E-06	1.38E-05	1.64E-05	0	0	0	2.23E-06	0	3.95E-06
k__Bacteria;p__Nitrospirae	0.02235857	0.01712441	0.02217755	0.02512152	0.02295891	0.02510752	0.02656865	0.0212316	0.01937786	0.01250016	0.0134578	0.01331261	0.01106333	0.01018042	0.01015949	1.21E-05	1.69E-05	1.64E-05	2.67E-05	4.70E-05	2.77E-05
k__Bacteria;p__OP11	3.23E-05	1.86E-05	3.48E-05	5.90E-05	3.07E-05	4.15E-05	4.16E-05	3.83E-05	7.30E-05	3.25E-05	2.72E-05	3.67E-05	4.25E-05	4.14E-05	2.79E-05	4.83E-06	2.12E-05	6.01E-05	5.35E-05	0	1.58E-05
k__Bacteria;p__OP3	0.00032708	0.00022643	0.00028193	0.00037	0.00043942	0.00045445	0.00029432	0.00020148	0.00033951	0.00024357	0.00037339	0.00040378	0.00023735	0.00025382	0.0002792	4.83E-06	4.23E-06	0	0	0	3.95E-06
k__Bacteria;p__Planctomycetes	0.06584816	0.05878247	0.06780502	0.06644189	0.07360135	0.07553982	0.06688069	0.06912634	0.07799111	0.07299718	0.07499857	0.07415848	0.0590015	0.06022587	0.05898284	0.07562788	0.08879009	0.10017545	0.08382699	0.05722609	0.05498016
k__Bacteria;p__Proteobacteria	0.36979069	0.38354859	0.35663496	0.34097605	0.35561437	0.33204517	0.34586107	0.36165324	0.32147475	0.30196547	0.28512303	0.2970377	0.3038951	0.29547633	0.30841746	0.24200777	0.20060578	0.26264901	0.2284629	0.28340537	0.32023924
k__Bacteria;p__SAR406	4.04E-06	5.73E-06	1.10E-05	1.16E-06	1.70E-06	4.98E-06	1.60E-06	0	3.95E-06	0	0	0	7.33E-06	1.38E-06	1.64E-06	2.41E-06	4.23E-06	5.47E-06	6.68E-06	0	0
k__Bacteria;p__SBR1093	1.21E-05	1.00E-05	1.83E-05	0	0	1.66E-06	0	0	0	0	0	0	0	0	0	0	0	0	0	0	0
k__Bacteria;p__SC3	6.26E-05	7.17E-05	0.00011168	6.01E-05	8.86E-05	5.97E-05	0.00014236	0.00010657	0.00011843	0.00010717	0.00014306	0.00015295	0.00011575	0.00014484	0.0001708	0.00034993	0.00024113	0.00043179	0.00049681	0.00108062	0.00085388
k__Bacteria;p__SC4	1.82E-05	1.86E-05	3.30E-05	3.70E-05	2.21E-05	8.29E-05	1.44E-05	1.17E-05	1.78E-05	2.27E-05	1.14E-05	1.22E-05	0.00032819	0.00024968	0.00024799	1.45E-05	3.38E-05	5.47E-06	2.67E-05	0	0.00013836
k__Bacteria;p__SM2F11	2.22E-05	3.01E-05	6.04E-05	4.97E-05	5.62E-05	4.48E-05	2.08E-05	4.16E-05	1.18E-05	6.50E-06	2.00E-05	9.18E-06	4.25E-05	3.86E-05	2.96E-05	4.83E-06	8.46E-06	5.47E-06	1.56E-05	0	7.91E-06
k__Bacteria;p__SPAM	0.01407657	0.01733508	0.01388065	0.0119046	0.01297655	0.01207111	0.01127208	0.01035188	0.00982809	0.00541057	0.00589986	0.00642994	0.00402623	0.00394664	0.00382993	0.00212853	0.00151022	0.00167797	0.00151048	0.00812817	0.00660173
k__Bacteria;p__SR1	0	0	7.32E-06	0	0	0	0	0	3.95E-06	6.50E-06	2.86E-06	3.06E-06	0	0	0	0	0	0	1.34E-05	4.70E-05	7.91E-06
k__Bacteria;p__Spirochaetes	1.21E-05	1.86E-05	2.01E-05	2.54E-05	2.04E-05	3.98E-05	1.92E-05	1.83E-05	3.75E-05	2.92E-05	2.72E-05	1.53E-05	2.93E-05	0.00011725	0.00027263	2.65E-05	2.54E-05	2.19E-05	8.91E-06	9.40E-05	2.77E-05
k__Bacteria;p__Synergistetes	0	0	0	0	0	0	0	0	0	0	0	0	0	1.10E-05	1.64E-06	0	0	0	0	0	0
k__Bacteria;p__TG3	2.02E-06	0	3.66E-06	1.16E-06	0	1.66E-06	0	3.33E-06	1.97E-06	3.25E-06	0	0	2.93E-06	1.24E-05	4.93E-06	3.86E-05	8.46E-05	0.00012571	0.00018268	9.40E-05	9.88E-05
k__Bacteria;p__TM6	0.00194633	0.00157644	0.0020248	0.0023171	0.00177472	0.00202844	0.00250011	0.00238108	0.00216538	0.00171475	0.00197711	0.00192409	0.0040526	0.00381973	0.00336515	0.0027584	0.00370999	0.00337781	0.00438662	0.00277204	0.00219399
k__Bacteria;p__TM7	1.01E-05	8.60E-06	1.46E-05	1.73E-05	5.11E-06	1.16E-05	8.00E-06	8.33E-06	3.95E-06	4.22E-05	6.01E-05	2.75E-05	7.33E-06	1.66E-05	8.21E-06	0	4.23E-06	0	4.46E-06	0	3.95E-06
k__Bacteria;p__Tenericutes	0.0002443	0.00022787	0.00017941	0.00030756	0.00024356	0.000136	0.00027512	0.00037964	0.00031385	0.00042219	0.00027039	0.00039155	0.00032673	0.00029107	0.00027427	0.00022685	0.00050341	0.00015851	0.00023615	9.40E-05	0.00016603
k__Bacteria;p__Thermotogae	0.00015345	3.73E-05	4.58E-05	4.62E-05	4.26E-05	2.99E-05	4.32E-05	8.16E-05	5.72E-05	1.62E-05	1.14E-05	3.06E-06	1.03E-05	1.10E-05	2.14E-05	3.86E-05	1.69E-05	5.47E-06	1.11E-05	0.00108062	0.0001937
k__Bacteria;p__Verrucomicrobia	0.12445613	0.10010662	0.13966695	0.15710229	0.14732532	0.14955874	0.17184363	0.15497688	0.17414811	0.18914249	0.18864092	0.18877788	0.14635361	0.14535377	0.14798921	0.31783595	0.28943817	0.29149154	0.30791218	0.23980455	0.19330814
k__Bacteria;p__WPS-2	0.00022815	0.00030382	0.00027461	0.00039774	0.00029295	0.00034996	0.00087976	0.00068935	0.0009909	0.00223438	0.00270815	0.00268271	0.0058474	0.00596203	0.00620146	0.00029925	0.00036804	0.00026782	0.00019605	0.00023492	0.00012255
k__Bacteria;p__WS1	0	0	0	1.16E-06	0	0	0	0	0	0	0	0	0	0	0	0	0	0	0	4.70E-05	0
k__Bacteria;p__WS2	2.02E-06	0	0	2.31E-06	0	0	1.60E-06	0	0	9.74E-06	2.86E-06	6.12E-06	5.86E-06	8.28E-06	8.21E-06	0	0	0	0	0	0
k__Bacteria;p__WS3	0.00691513	0.00731324	0.00705932	0.0051464	0.0085074	0.00788653	0.00461632	0.00456236	0.00453999	0.00371855	0.00305293	0.00321803	0.00119703	0.0014443	0.00132208	0.0006709	0.00048226	0.00150853	0.00155058	0.01442398	0.00743189
k__Bacteria;p__WS6	2.22E-05	2.72E-05	1.28E-05	2.54E-05	2.55E-05	4.15E-05	3.84E-05	4.00E-05	3.16E-05	3.25E-05	1.57E-05	1.22E-05	2.64E-05	3.45E-05	2.79E-05	2.41E-06	1.27E-05	1.09E-05	2.67E-05	0	3.95E-06
k__Bacteria;p__ZB2	2.83E-05	2.72E-05	6.41E-05	4.97E-05	4.43E-05	5.47E-05	4.00E-05	3.33E-05	2.57E-05	1.30E-05	1.86E-05	2.45E-05	5.27E-05	3.59E-05	5.75E-05	1.21E-05	4.23E-06	2.19E-05	1.11E-05	9.40E-05	7.12E-05
k__Bacteria;p__ZB3	2.02E-06	1.43E-06	0	0	1.70E-06	0	0	0	0	3.25E-06	1.43E-06	0	2.93E-06	0	1.64E-06	0	0	0	0	0	0
