# Supplementary material for: New Biological Insights Into How Deforestation in Amazonia Affects Soil Microbial Communities Using Metagenomics and Metagenome-Assembled Genomes
Source: Front Microbiol. 2018 Jul 23;9:1635. doi: 10.3389/fmicb.2018.01635 (PMC6064768; doi:10.3389/fmicb.2018.01635)
Supplement: Supplementary file 13 [file Table_7.PDF]

Supplemental Table 7: The unique protein families (FIGFams) found only in Rokubacteria Amazon FNV 2010 15_13 when compared to 75 other Rokubacteria MAGs.

Family ID	Description
FIG00517239	FIG00517240: hypothetical protein
FIG00000279	GTPase and tRNA-U34 5-formylation enzyme TrmE
FIG00000541	2-oxoglutarate dehydrogenase E1 component (EC 1.2.4.2)
FIG00000552	Cell division protein FtsX
FIG00000756	Ribosomal-protein-S5p-alanine acetyltransferase
FIG00001000	Sulfate and thiosulfate binding protein CysP
FIG00001036	Type II/IV secretion system ATP hydrolase TadA/VirB11/CpaF, TadA subfamily
FIG00001109	4-hydroxyphenylpyruvate dioxygenase (EC 1.13.11.27)
FIG00001409	5-deoxy-glucuronate isomerase (EC 5.3.1.-)
FIG00001495	Glutaredoxin-related protein
FIG00002117	L-arabinose isomerase (EC 5.3.1.4)
FIG00002300	Ribulokinase (EC 2.7.1.16)
FIG00002561	RNA polymerase sporulation specific sigma factor SigH
FIG00003506	Cytosine/purine/uracil/thiamine/allantoin permease family protein
FIG00003590	Streptothricin acetyltransferase, Streptomyces lavendulae type
FIG00004410	Aspartate racemase (EC 5.1.1.13)
FIG00004443	4-hydroxycinnamoyl CoA hydratase/lyase (Enoyl-CoA hydratase/lyase) (EC 4.2.1.17)
FIG00005527	Catechol 2,3-dioxygenase (EC 1.13.11.2)
FIG00005590	Hypothetical protein DUF194, DegV family
FIG00006767	Gamma-aminobutyrate:alpha-ketoglutarate aminotransferase (EC 2.6.1.19)
FIG00007212	GDP-mannose 6-dehydrogenase (EC 1.1.1.132)
FIG00007989	Guanine deaminase (EC 3.5.4.3); Hydroxydechloroatrazine ethylaminohydrolase (EC 3.5.99.3)
FIG00008311	Inositol transport system sugar-binding protein
FIG00008439	Inositol transport system ATP-binding protein
FIG00014104	Encapsulating protein for a DyP-type peroxidase or ferritin-like protein oligomers
FIG00015316	Flp pilus assembly protein TadB
FIG00016929	HoxN/HupN/NixA family nickel/cobalt transporter
FIG00017495	Nudix-related transcriptional regulator NrtR
FIG00018897	Sulfate transport system permease protein CysT
FIG00029322	Aerobic C4-dicarboxylate transporter for fumarate, L-malate, D-malate, succunate
FIG00029868	Epi-inositol hydrolase (EC 3.7.1.-)
FIG00046358	Transport ATP-binding protein CydC
FIG00049476	FIG049476: HIT family protein
FIG00066718	Putative phosphoenolpyruvate synthase/pyruvate phosphate dikinase, C-terminal domain
FIG00096593	Cytochrome d ubiquinol oxidase subunit I (EC 1.10.3.-)
FIG00134749	Menaquinone-specific isochorismate synthase (EC 5.4.4.2)
FIG00137607	Proline racemase (EC 5.1.1.4)
FIG00137661	Predicted ATPase with chaperone activity, associated with Flp pilus assembly
FIG00138458	Isocitrate dehydrogenase [NAD] (EC 1.1.1.41)
FIG00138542	Predicted nucleoside ABC transporter, substrate-binding component
FIG00139157	Type II/IV secretion system protein TadC, associated with Flp pilus assembly
FIG00139160	P-hydroxybenzoate hydroxylase (EC 1.14.13.2)
FIG00149264	Particulate methane monooxygenase A-subunit (EC 1.14.13.25)
FIG00149431	Particulate methane monooxygenase C-subunit (EC 1.14.13.25)
FIG00229257	Histidinol-phosphatase (EC 3.1.3.15)
FIG00229345	Thermostable carboxypeptidase 1 (EC 3.4.17.19)
FIG00340333	FIG021292: hypothetical protein
FIG00351520	Putative siderophore biosynthesis protein, related to 2-demethylmenaquinone methyltransferase
FIG00449297	Similar to vanillate/3-O-methylgallate O-demethylase
FIG00451192	sugar ABC transporter, periplasmic sugar-binding protein

FIG00506717	FIG173306: hypothetical protein
FIG00000390	Cytochrome d ubiquinol oxidase subunit II (EC 1.10.3.-)
FIG00528069	Probable 2-phosphosulfolactate phosphatase (EC 3.1.3.71)
FIG00553659	FMN-dependent NADH-azoreductase
FIG00628138	Transcriptional regulator, TetR family
FIG00628194	Acid-resistant locus arl7 (Fragment)
FIG00634996	Mn-dependent transcriptional regulator MntR
FIG00638731	Transglycosylase, Slt family
FIG00680972	pyridoxamine 5'-phosphate oxidase-related, FMN-binding
FIG00682872	protein of unknown function DUF1326
FIG00774028	TPR domain protein
FIG00821228	O-methyltransferase
FIG00868571	putative two-component system sensor kinase
FIG00869624	Ribosomal large subunit pseudouridine synthase B (EC 4.2.1.70)
FIG00945488	FHA domain containing protein
FIG00973639	macromolecule metabolism; macromolecule synthesis, modification
FIG00983407	putative permease component of ABC transporter
FIG01000155	FIG01000157: hypothetical protein
FIG01010650	Transport ATP-binding protein CydD
FIG01030543	FIG01030554: hypothetical protein
FIG01076290	Aminomethyltransferase
FIG01121797	putative RNA polymerase sigma factor
FIG01122886	putative drug exporters of the RND superfamily
FIG01125468	FIG01125469: hypothetical protein
FIG01132099	Methyltransferase SCO0408
FIG01227591	Trp repressor binding protein
FIG01239241	oxidoreductase of aldo/keto reductase family, subgroup 1
FIG01272192	Sulfate transport system permease protein CysW
FIG01304044	Molybdopterin binding motif, CinA N-terminal domain / C-terminal domain of CinA type S
FIG01309964	Glucose/sorbosone dehydrogenases
FIG01319990	lysophospholipase-like family protein
FIG01322870	Saccharopine dehydrogenase and related proteins
FIG01326115	pseudomonapepsin precursor
FIG01327653	oxidoreductase ylbE
FIG01333307	glycosyl transferase, family 39
FIG01339311	oxidoreductase, FAD-binding
FIG01344107	cation-transporting ATPase, E1-E2 family
FIG01347954	basic membrane lipoprotein
FIG01348972	Diadenosine tetraphosphatase
FIG01350540	Cocaine esterase (EC 3.1.1.-)
FIG01354495	protein of unknown function DUF151
FIG01361711	Hypothetical N-acetyltransferase
FIG01386881	monooxygenase, flavin-binding family
FIG01430130	YceI like family protein
FIG01437391	DEAD/DEAH box helicase-like
FIG01455347	Peptidase T (EC 3.4.11.-)
FIG01499937	Uncharacterized membrane protein
FIG01508617	Glyceraldehyde-3-phosphate dehydrogenase, putative
FIG01513806	3',5'-cyclic-nucleotide phosphodiesterase
FIG01704471	probable 3-oxoacyl-(acyl carrier protein) reductase
FIG01709650	Amidases related to nicotinamidase
FIG01859502	FIG00840365: hypothetical protein
FIG01954108	Flavodoxin reductases (ferredoxin-NADPH reductases) family 1
