# Supplementary material for: New Biological Insights Into How Deforestation in Amazonia Affects Soil Microbial Communities Using Metagenomics and Metagenome-Assembled Genomes
Source: Front Microbiol. 2018 Jul 23;9:1635. doi: 10.3389/fmicb.2018.01635 (PMC6064768; doi:10.3389/fmicb.2018.01635)
Supplement: Supplementary file 14 [file Table_8.PDF]

Supplemental Table 8: Genes originating from lateral gene transfer in Rokubacteria Amazon FNV 2010 15_13 as identified by CompareM. The complete output is available upon request.

Gene Id	Length (bp)	Annotation of deviants	Deviations from mean
fig|6666666.168244.peg.3060	4971	General secretion pathway protein N	2.85
fig|6666666.168244.peg.884	1536	Basic proline rich protein precursor	5.42
fig|6666666.168244.peg.3138	2232	Hypothetical protein	3.70
fig|6666666.168244.peg.1411	1866	Basic proline rich protein precursor	3.56
fig|6666666.168244.peg.266	147	Dipeptide transport system permease DppC	0.76
fig|6666666.168244.peg.3100	1290	Basic proline rich protein precursor	5.16
fig|6666666.168244.peg.1595	1305	Hypothetical protein	1.69
fig|6666666.168244.peg.1306	2901	PE-PGRS	1.64
fig|6666666.168244.peg.1991	1395	Hypothetical protein	2.18
fig|6666666.168244.peg.1367	153	Hypothetical protein	1.23
fig|6666666.168244.peg.2626	1383	Hypothetical protein	2.79
fig|6666666.168244.peg.2780	2865	Putative autotransporter	1.42
fig|6666666.168244.peg.769	1056	Hypothetical protein	3.57
fig|6666666.168244.peg.2126	645	Hypothetical protein	3.23
fig|6666666.168244.peg.3182	2739	Regulatory protein LuxR	0.18
fig|6666666.168244.peg.2499	126	Hypothetical protein	2.06
fig|6666666.168244.peg.2217	1998	Beta-hexosaminidase	0.82
fig|6666666.168244.peg.1616	5028	Conserved repeat domain	1.14
fig|6666666.168244.peg.801	123	Hypothetical protein	3.12
fig|6666666.168244.peg.744	327	LSU L24p	1.50
fig|6666666.168244.peg.2054	636	Basic proline rich protein precursor	3.89
fig|6666666.168244.peg.34	171	Hypothetical protein	2.79
fig|6666666.168244.peg.2705	750	Hypothetical protein	3.15
fig|6666666.168244.peg.2912	1416	Probable poly(beta-D-mannuronate) O-acetylase	0.36
fig|6666666.168244.peg.1280	1788	Choline dehydrogenase	1.52
fig|6666666.168244.peg.2305	1452	Hypothetical protein	2.38
fig|6666666.168244.peg.939	123	N-methylhydantoinase A	2.59
fig|6666666.168244.peg.2840	3111	RND multidrug efflux transporter; acriflavine resistance	-0.04

fig|6666666.168244.peg.667	927	Collagen triple helix repeat domain	0.57
fig|6666666.168244.peg.1446	114	Hypothetical protein	2.89
fig|6666666.168244.peg.1222	171	Hypothetical protein	1.89
fig|6666666.168244.peg.1548	2139	Hypothetical protein	1.33
fig|6666666.168244.peg.2979	1098	TolA precursor	1.28
fig|6666666.168244.peg.2221	630	Hypothetical protein	1.89
fig|6666666.168244.peg.2586	960	Enoyl-[acyl-carrier-protein] reductase [FMN]	1.23
fig|6666666.168244.peg.2232	201	Hypothetical protein	2.85
fig|6666666.168244.peg.480	135	Hypothetical protein	2.94
fig|6666666.168244.peg.2583	588	Possible transmembrane protein	0.91
fig|6666666.168244.peg.2673	2070	Hypothetical protein	0.45
fig|6666666.168244.peg.1824	1473	Malonate transporter	1.26
fig|6666666.168244.peg.1773	114	Hypothetical protein	2.72
fig|6666666.168244.peg.2192	1077	Cell surface protein	1.38
fig|6666666.168244.peg.427	2034	DNA ligase	-0.47
fig|6666666.168244.peg.1281	267	Hypothetical protein	2.91
fig|6666666.168244.peg.2523	195	Hypothetical protein	2.01
fig|6666666.168244.peg.2723	126	Hypothetical protein	3.36
fig|6666666.168244.peg.2173	825	Inner membrane protein translocase YidC long form	1.50
fig|6666666.168244.peg.2125	252	Hypothetical protein	1.58
fig|6666666.168244.peg.1854	177	Hypothetical protein	3.56
fig|6666666.168244.peg.1637	1704	Hypothetical protein	1.99
fig|6666666.168244.peg.1675	378	Hypothetical protein	0.34
fig|6666666.168244.peg.1497	2091	Hypothetical protein	1.16
fig|6666666.168244.peg.1452	1602	LipT	1.77
fig|6666666.168244.peg.1805	432	Chemotaxis protein methyltransferase CheR	0.28
fig|6666666.168244.peg.1775	1215	COG0477:permeases of major facilitator superfamily	0.94
fig|6666666.168244.peg.1486	213	Hypothetical protein	3.77
fig|6666666.168244.peg.2763	153	Hypothetical protein	3.52
fig|6666666.168244.peg.1609	2826	Putative transcriptional regulator	-0.39
fig|6666666.168244.peg.469	390	Hypothetical protein	3.28

fig|6666666.168244.peg.452	2715	Macrolide-specific ABC-type efflux carrier	-0.29
fig|6666666.168244.peg.224	1062	Epoxide hydrolase	1.16
fig|6666666.168244.peg.1179	114	Hypothetical protein	3.58
fig|6666666.168244.peg.2907	1434	Gamma-glutamyltranspeptidase	0.17
fig|6666666.168244.peg.891	177	Hypothetical protein	3.45
fig|6666666.168244.peg.626	996	Ribose ABC transport system permease RbsC	1.15
fig|6666666.168244.peg.2677	1005	Similarity with known prokaryotic or eukaryotic proteins	0.99
fig|6666666.168244.peg.570	159	Hypothetical protein	1.65
fig|6666666.168244.peg.1019	732	Peptidase M23B	-0.13
fig|6666666.168244.peg.2313	165	Hypothetical protein	2.48
fig|6666666.168244.peg.650	2046	N-methylhydantoinase A	-0.02
fig|6666666.168244.peg.1699	606	Transcriptional regulator TetR family	0.01
fig|6666666.168244.peg.2784	1446	Alpha-galactosidase	1.01
fig|6666666.168244.peg.6	1509	Alanyl-dipeptidyl peptidase	2.11
fig|6666666.168244.peg.1770	222	Hypothetical protein	2.41
fig|6666666.168244.peg.821	1329	Membrane protein SypL involved in exopolysaccharide production	0.08
fig|6666666.168244.peg.1622	2505	Mannose-1-phosphate guanylyltransferase/phosphomannomutase	-0.71
fig|6666666.168244.peg.2948	819	RNA binding methyltransferase FtsJ-like	-0.03
fig|6666666.168244.peg.2774	261	Protein yceI precursor	1.88
fig|6666666.168244.peg.497	129	Hypothetical protein	3.26
fig|6666666.168244.peg.2304	117	Hypothetical protein	3.71
fig|6666666.168244.peg.625	918	Ribose ABC transport system permease protein RbsC	0.58
fig|6666666.168244.peg.810	186	Hypothetical protein	3.51
fig|6666666.168244.peg.557	756	3-oxoacyl-[acyl-carrier-protein] reductase	0.68
fig|6666666.168244.peg.1524	693	Hypothetical protein	1.12
fig|6666666.168244.peg.2831	123	Hypothetical protein	1.76
fig|6666666.168244.peg.3085	783	3-oxoacyl-[acyl-carrier-protein] reductase	0.65
fig|6666666.168244.peg.1388	381	Arsenate reductase	0.65
fig|6666666.168244.peg.3094	249	Hypothetical protein	1.52

fig|6666666.168244.peg.483	213	Hypothetical protein	1.79
fig|6666666.168244.peg.947	1686	Hypothetical protein	0.89
fig|6666666.168244.peg.410	2961	Adenylate cyclase/guanylate cyclase	0.08
fig|6666666.168244.peg.871	3066	Hypothetical protein	0.48
fig|6666666.168244.peg.1253	1830	Hypothetical protein	0.77
fig|6666666.168244.peg.271	1416	Aspartyl-tRNA(Asn) amidotransferase subunit A @ glutamyl-tRNA(Gln) amidotransferase subunit A	0.70
fig|6666666.168244.peg.1338	174	Hypothetical protein	3.57
fig|6666666.168244.peg.2290	864	Carbon monoxide dehydrogenase medium chain	0.19
fig|6666666.168244.peg.1952	420	Hypothetical protein	1.61
fig|6666666.168244.peg.2414	126	Hypothetical protein	2.40
fig|6666666.168244.peg.2738	936	Quinolinate synthetase	-0.14
fig|6666666.168244.peg.2022	1134	Hypothetical protein	0.38
fig|6666666.168244.peg.168	714	Glycosyl transferase family 2	0.07
fig|6666666.168244.peg.2001	618	Cysteinyl-tRNA synthetase	4.08
