# Supplementary material for: New Biological Insights Into How Deforestation in Amazonia Affects Soil Microbial Communities Using Metagenomics and Metagenome-Assembled Genomes
Source: Front Microbiol. 2018 Jul 23;9:1635. doi: 10.3389/fmicb.2018.01635 (PMC6064768; doi:10.3389/fmicb.2018.01635)
Supplement: Supplementary file 15 [file Table_9.PDF]

Supplemental Table 9: Overview for the presence/absence of key metabolic genes found in Rokubacteria MAGs (n=53).

		Hydrocarbon degradation steps						Beta-oxidation of fatty acids				Cytochromes/Electron Transport													
Study Location	Rokubacteria Metagenome-Assembled Genome	alkane monooxygenase	P450 hydroxylase	particulate methane monooxygenase	aldehyde dehydrogenase	alcohol dehydrogenase	acyl-CoA synthetase	acyl CoA dehydrogenase	enoyl CoA hydratase	3-hydroxyacyl-CoA dehydrogenase	β-ketothiolase	NADH:ubiquinone oxidoreductase	NADH dehydrogenase	anaerobic DMSO reductase	anaerobic nitric oxide reductase flavorubredoxin	arsenate reductase	fumarate reductase	mercuric ion reductase	cytochrome bc1 complex	cytochrome c oxidase	cytochrome c551 peroxidase	flavocytochrome c sulfide dehydrogenase	sulfite dehydrogenase cytochrome SoxD	cytochrome d ubiquinol oxidase	electron transport RNF complex
Amazon rainforest (this study)	Rokubacteria_Amazon_FNV_2010_15_13	X	X	X	X	X	X		X		X		X			X				X				X	
Rifle, CO aquifer (Banfield Lab)	RIFCSPHIGHO2_12_FULL_73_22	X				X	X	X	X	X	X	X	X	X		X	X	X	X	X					
Rifle, CO aquifer (Banfield Lab)	RIFCSPLOWO2_02_FULL_69_13				X	X	X	X	X	X	X	X	X	X		X			X	X		X			
Rifle, CO aquifer (Banfield Lab)	RISCPLOWO2_12_FULL_68_19				X	X	X	X	X	X	X	X	X	X		X	X		X	X	X	X			
Rifle, CO aquifer (Banfield Lab)	RIFCSPLOWO2_12_FULL_69_21				X	X	X	X	X	X	X	X		X					X	X					
Rifle, CO aquifer (Banfield Lab)	RIFSCPLOWO2_12_FULL_71_18						X	X	X		X	X		X						X					X
Rifle, CO aquifer (Banfield Lab)	RIFCPLOWO2_12_FULL_71_19					X	X	X	X	X	X	X	X				X								X
Rifle, CO aquifer (Banfield Lab)	RIFCSPLOWO2_12_FULL_71_22				X	X	X	X	X	X	X	X	X		X	X			X	X	X	X			X
Rifle, CO aquifer (Banfield Lab)	RIFCSPLOWO2_12_FULL_72_37					X	X	X	X	X	X	X					X		X	X					X
Rifle, CO aquifer (Banfield Lab)	RIFSCPLOWO2_12_FULL_73_26	X				X	X	X	X		X	X	X	X	X	X	X	X	X	X					X
Rifle, CO aquifer (Banfield Lab)	RIFCSPLOWO2_12_FULL_73_47	X				X	X	X	X	X	X	X	X	X	X	X	X	X	X	X					X
Rifle, CO aquifer (Banfield Lab)	RIFCPLOWO2_12_FULL_73_56	X				X	X	X	X	X	X	X	X	X	X	X		X	X	X					X
California meadow soil (Banfield Lab)	13_1_20CM_2_68_19		X		X	X	X	X	X		X	X	X				X			X					
California meadow soil (Banfield Lab)	13_1_20CM_2_69_58		X		X	X	X	X	X	X	X	X	X							X		X			
California meadow soil (Banfield Lab)	13_1_20CM_2_70_7				X	X	X	X	X	X	X	X	X							X					
California meadow soil (Banfield Lab)	13_1_20CM_4_68_9	X	X		X	X	X		X	X	X	X	X		X	X	X		X	X	X	X	X		
California meadow soil (Banfield Lab)	13_1_20CM_4_70_13				X		X	X	X			X								X		X			
California meadow soil (Banfield Lab)	13_1_20CM_4_70_14		X			X	X		X		X	X	X				X			X			X		
California meadow soil (Banfield Lab)	13_1_20CM_70_15		X			X	X	X	X			X	X					X					X		
California meadow soil (Banfield Lab)	13_1_40CM_2_68_13					X	X		X			X				X	X	X		X	X	X			
California meadow soil (Banfield Lab)	13_1_40CM_2_68_8						X		X		X	X				X				X		X			
California meadow soil (Banfield Lab)	13_1_40CM_2_70_45		X			X	X	X	X		X	X								X		X			
California meadow soil (Banfield Lab)	13_1_40CM_3_69_38		X			X	X	X	X			X	X				X			X		X			
California meadow soil (Banfield Lab)	13_1_40CM_4_67_11	X	X			X	X	X	X		X	X	X			X	X			X		X			
California meadow soil (Banfield Lab)	13_1_40CM_4_69_39		X			X	X	X	X	X		X	X				X			X		X			
California meadow soil (Banfield Lab)	13_1_40CM_4_69_5	X	X		X	X	X	X	X	X	X	X	X				X			X		X	X		
California meadow soil (Banfield Lab)	13_1_40CM_68_15	X	X		X	X	X		X	X	X	X	X		X	X	X			X		X	X		
California meadow soil (Banfield Lab)	13_1_40CM_69_27	X	X		X	X	X	X	X	X	X	X	X				X			X		X	X		
California meadow soil (Banfield Lab)	13_1_40CM_69_96	X	X		X		X		X		X	X					X			X					
California meadow soil (Banfield Lab)	13_2_20CM_2_64_8	X	X		X	X	X	X	X	X	X	X	X			X	X		X	X	X	X	X		
California meadow soil (Banfield Lab)	13_2_20CM_2_70_11	X	X		X	X	X	X	X		X	X	X				X			X	X	X			
California meadow soil (Banfield Lab)	13_2_20CM_69_10	X			X	X	X	X	X	X	X	X	X				X		X	X		X			
California meadow soil (Banfield Lab)	13_2_20CM_69_15_1	X	X		X	X	X	X	X	X	X	X	X				X			X		X			
California meadow soil (Banfield Lab)	13_2_20CM_69_15_2						X		X		X				X										
California meadow soil (Banfield Lab)	13_2_20CM_70_12						X		X		X		X												
Rifle, CO aquifer (Banfield Lab)	GWA2_70_23				X	X	X	X	X	X	X	X		X		X	X		X	X	X				X
Rifle, CO aquifer (Banfield Lab)	GWA2_73_35				X	X	X	X	X		X	X				X	X		X	X	X	X			X
Rifle, CO aquifer (Banfield Lab)	GWC2_70_16					X	X	X	X	X	X	X		X		X			X	X					X
Rifle, CO aquifer (Banfield Lab)	GWC2_70_24					X	X	X	X	X	X	X		X			X								X
Rifle, CO aquifer (Banfield Lab)	GWF2_70_14				X	X	X	X	X		X	X					X			X					X
Rifle, CO aquifer (Banfield Lab)	RBG_16_73_20				X	X	X	X	X		X	X		X			X		X	X	X	X			X
Rifle, CO aquifer (Banfield Lab)	RIFCSPHIGHO2_02_FULL_69_13				X	X	X	X	X	X	X	X		X		X			X	X		X			
Rifle, CO aquifer (Banfield Lab)	RIFCSPHIGHO2_02_FULL_73_26					X	X	X	X		X	X	X	X	X	X	X	X	X	X					X
Rifle, CO aquifer (Banfield Lab)	RIFCSPHIGHO2_12_FULL_73_22					X	X	X	X	X	X	X	X	X		X	X	X	X	X					
Rifle, CO aquifer (Banfield Lab)	RIFCSPLOWO2_02_FULL_68_19				X	X	X	X	X	X	X	X		X		X	X		X	X	X	X			
Rifle, CO aquifer (Banfield Lab)	RIFCSPLOWO2_02_FULL_71_18						X	X	X		X	X		X						X					X
Rifle, CO aquifer (Banfield Lab)	RIFCSPLOWO2_02_FULL_72_37					X	X	X	X	X	X	X					X		X	X					X
Rifle, CO aquifer (Banfield Lab)	RIFCSPLOWO2_02_FULL_73_56					X	X	X	X	X	X	X	X	X	X	X	X	X	X	X					X
Rifle, CO aquifer (Banfield Lab)	RIFCSPLOWO2_12_FULL_69_21				X	X	X	X	X	X	X	X		X					X	X					
Rifle, CO aquifer (Banfield Lab)	RIFCSPLOWO2_12_FULL_71_19					X	X	X	X	X	X	X					X								X
Rifle, CO aquifer (Banfield Lab)	RIFCSPLOWO2_12_FULL_71_22				X	X	X	X	X	X	X	X	X		X	X			X	X	X	X			X
Rifle, CO aquifer (Banfield Lab)	RIFCSPLOWO2_12_FULL_73_47					X	X	X	X	X	X	X	X	X	X	X	X	X	X	X					X
Rifle, CO aquifer (Banfield Lab)	bacterium CSP1-6	X			X	X	X	X	X	X	X	X		X		X				X		X			

	Benzoate metabolism (ben-cat pathway)										Benzoate metabolism (box pathway)				Anaerobic Benzoate Metabolism		Aromatic compound oxidation genes			Biphenyl metabolism (bph pathway)						
Rokubacteria Metagenome-Assembled Genome	benzoate-1,2-dioxygenase	cis-1,2-dihydroxycyclohexa-3,5-diene-1-carboxylate dehydrogenase	catechol-1,2-dioxygenase	catechol-2,3-dioxygenase	muconate cycloisomerase	muconolactone δ-isomerase	3-oxoadipate enol-lactonase	3-oxoadipate CoA-transferase	β-ketoadipyl-CoA thiolase	benzoate 4-monooxygenase	benzoate CoA ligase	benzoyl-CoA oxygenase/reductase	benzoyl-CoA dihydrodiol lyase	aldehyde dehydrogenase	benzoyl-CoA reductase	hydroxyacyl-CoA dehydrogenase	4-hydroxyphenylacetate 3-monooxygenase	protocatechuate 3,4-dioxygenase	gentisate 1,2-dioxygenase	biphenyl dioxygenase	biphenyldihydrodiol dehydrogenase	2,3-dihydroxybiphenyl 1,2-dioxygenase	2-hydroxy-6-oxo-6-phenylhexa-2,4-dienoate hydrolase	2-hydroxypenta-2,4-dienoate hydratase	4-hydroxy-2-oxovalerate aldolase	acetaldehyde dehydrogenase
Rokubacteria_Amazon_FNV_2010_15_13				X	X		X	X	X					X			X									
RIFCSPHIGHO2_12_FULL_73_22					X		X		X		X	X	X		X	X	X					X	X			
RIFCSPLOWO2_02_FULL_69_13							X		X			X		X		X			X							
RISCPLOWO2_12_FULL_68_19							X	X	X		X	X	X	X		X										
RIFCSPLOWO2_12_FULL_69_21							X	X	X					X	X	X			X							
RIFSCPLOWO2_12_FULL_71_18					X		X		X				X		X				X							
RIFCPLOWO2_12_FULL_71_19					X		X		X			X	X		X	X	X						X			
RIFCSPLOWO2_12_FULL_71_22							X		X		X	X	X	X		X							X			
RIFCSPLOWO2_12_FULL_72_37							X		X		X	X	X			X	X						X			
RIFSCPLOWO2_12_FULL_73_26					X			X	X		X	X	X		X	X	X						X			
RIFCSPLOWO2_12_FULL_73_47					X		X		X		X	X	X		X	X	X						X			
RIFCPLOWO2_12_FULL_73_56					X		X		X		X	X	X		X	X	X						X			
13_1_20CM_2_68_19	X							X	X			X	X	X		X	X	X	X							
13_1_20CM_2_69_58							X		X			X	X	X		X	X		X			X				
13_1_20CM_2_70_7									X					X												
13_1_20CM_4_68_9							X	X	X			X	X	X		X	X		X			X				
13_1_20CM_4_70_13	X		X													X			X							
13_1_20CM_4_70_14								X	X			X	X				X									
13_1_20CM_70_15																										
13_1_40CM_2_68_13								X	X			X	X				X									
13_1_40CM_2_68_8			X					X	X		X	X	X			X		X								
13_1_40CM_2_70_45									X																	
13_1_40CM_3_69_38									X			X	X				X									
13_1_40CM_4_67_11									X		X	X	X			X		X								
13_1_40CM_4_69_39								X	X			X	X			X	X									
13_1_40CM_4_69_5							X	X	X		X	X	X	X		X			X							
13_1_40CM_68_15							X		X		X	X	X	X		X	X		X			X	X			
13_1_40CM_69_27							X	X	X		X	X	X	X		X			X			X				
13_1_40CM_69_96					X		X		X			X	X	X					X							
13_2_20CM_2_64_8	X						X		X		X	X	X	X		X	X		X			X				
13_2_20CM_2_70_11							X		X		X	X	X	X		X			X			X				
13_2_20CM_69_10	X								X		X	X	X	X		X	X		X			X	X			
13_2_20CM_69_15_1								X	X		X	X	X	X		X	X		X			X				
13_2_20CM_69_15_2									X		X	X	X				X									
13_2_20CM_70_12								X	X								X									
GWA2_70_23									X				X	X	X	X	X						X			
GWA2_73_35									X		X	X		X	X	X	X						X			
GWC2_70_16							X		X				X		X	X	X						X			
GWC2_70_24									X							X										
GWF2_70_14								X	X					X		X	X									
RBG_16_73_20					X		X		X				X	X	X	X	X						X			
RIFCSPHIGHO2_02_FULL_69_13							X		X			X		X		X										
RIFCSPHIGHO2_02_FULL_73_26					X			X	X		X	X	X		X	X	X						X			
RIFCSPHIGHO2_12_FULL_73_22					X		X		X		X	X	X		X	X	X						X			
RIFCSPLOWO2_02_FULL_68_19							X	X	X		X	X	X	X		X			X							
RIFCSPLOWO2_02_FULL_71_18					X		X		X				X		X				X							
RIFCSPLOWO2_02_FULL_72_37							X		X		X	X	X			X	X									
RIFCSPLOWO2_02_FULL_73_56					X		X		X		X	X	X		X	X	X						X			
RIFCSPLOWO2_12_FULL_69_21								X	X					X	X	X			X							
RIFCSPLOWO2_12_FULL_71_19							X		X						X	X	X						X			
RIFCSPLOWO2_12_FULL_71_22							X		X		X	X	X	X		X							X			
RIFCSPLOWO2_12_FULL_73_47							X		X		X	X	X		X	X	X						X			
bacterium CSP1-6							X		X			X	X	X		X			X				X			

	Serine Cycle										RuMP cycle					Methane Oxidation											Glycolysis/Gluconeogenesis												
Rokubacteria Metagenome-Assembled Genome	serine hydroxymethyltransferase	serine-glyoxylate aminotransferase	hydroxypyruvate reductase	glycerate kinase	enolase	phosphoenolpyruvate carboxylase	malate dehydrogenase	malate thiokinase	malyl-CoA lyase	isocitrate lyase	hexulose-6-phosphate isomerase	glucose phosphate isomerase	glucose-6-phosphate dehydrogenase;	6-phosphogluconate dehydrogenase	hexulose-6-phosphate synthetase	methane monooxygenase	methanol dehydrogenase/catalase/peroxidase	formaldehyde dehydrogenase	tungsten-containing aldehyde oxidoreductase	tungsten-containing formate dehydrogenase	formate dehydrogenase	formate hydrogenlyase	formate--tetrahydrofolate ligase	methylene tetrahydrofolate dehydrogenase/methenyltetrahydrofolate cyclohydrolase	formaldehyde activting enzyme	methylene tetrahydromethanopterin dehydrogenase	PEP carboxykinase	enolase	phosphoglycerate mutase	phosphoglycerate kinase	glyceraldehyde phosphate dehydrogenase	triose phosphate isomerase	fructose-bisphosphate aldolase class I	fructose 1,6-bisphosphatase	glucose-6-phosphate isomerase	phosphoglucomutase	glucokinase	6-phosphofructokinase	pyrvate kinase
Rokubacteria_Amazon_FNV_2010_15_13		X		X	X		X						X	X		X	X						X	X			X	X	X	X	X	X		X	X		X	X	X
RIFCSPHIGHO2_12_FULL_73_22		X	X	X	X		X			X			X	X					X		X	X	X	X				X		X	X	X	X	X			X	X	
RIFCSPLOWO2_02_FULL_69_13	X	X		X	X					X			X	X					X	X	X		X	X				X		X	X	X	X		X				
RISCPLOWO2_12_FULL_68_19	X	X		X	X		X			X			X	X					X	X			X	X				X	X	X	X	X		X					X
RIFCSPLOWO2_12_FULL_69_21		X		X	X		X						X	X					X	X	X		X					X		X	X	X	X			X	X	X	
RIFSCPLOWO2_12_FULL_71_18	X	X					X						X	X					X											X	X	X	X		X				
RIFCPLOWO2_12_FULL_71_19				X	X					X			X						X		X		X	X			X	X		X	X	X	X	X			X	X	
RIFCSPLOWO2_12_FULL_71_22	X	X		X	X								X	X					X		X	X	X	X			X	X		X	X	X	X		X		X		X
RIFCSPLOWO2_12_FULL_72_37	X	X			X		X						X	X					X		X	X	X	X			X	X		X	X	X			X			X	X
RIFSCPLOWO2_12_FULL_73_26	X	X			X		X			X			X	X					X		X	X	X	X				X		X	X	X	X	X	X		X	X	X
RIFCSPLOWO2_12_FULL_73_47	X	X		X	X		X			X			X	X					X		X	X	X	X				X		X	X	X	X	X			X		X
RIFCPLOWO2_12_FULL_73_56		X		X	X		X			X			X	X					X		X	X	X	X				X		X	X	X	X	X			X		X
13_1_20CM_2_68_19		X			X							X	X	X			X		X		X	X						X	X			X			X	X		X	
13_1_20CM_2_69_58	X	X			X		X						X				X						X	X	X	X		X	X	X	X		X	X	X		X	X	
13_1_20CM_2_70_7	X	X		X			X						X	X					X		X						X			X	X	X	X	X	X		X		
13_1_20CM_4_68_9	X	X			X	X	X		X	X		X	X	X			X		X		X			X			X	X		X	X	X	X		X		X	X	
13_1_20CM_4_70_13							X																				X										X		
13_1_20CM_4_70_14	X	X		X	X					X		X	X	X			X		X		X	X						X			X	X	X	X	X	X	X	X	X
13_1_20CM_70_15	X	X			X							X	X	X			X				X	X		X				X					X	X	X	X			
13_1_40CM_2_68_13		X											X	X			X		X		X						X		X				X			X	X	X	
13_1_40CM_2_68_8	X	X											X								X	X	X				X		X										X
13_1_40CM_2_70_45	X	X			X								X				X											X			X	X	X	X	X		X	X	X
13_1_40CM_3_69_38	X	X		X	X		X					X	X	X			X		X				X					X			X	X	X	X	X	X	X	X	X
13_1_40CM_4_67_11	X	X		X									X	X			X		X		X	X					X			X	X	X	X			X	X	X	X
13_1_40CM_4_69_39	X	X		X	X		X			X		X	X	X			X		X		X	X	X					X			X	X			X			X	X
13_1_40CM_4_69_5	X	X		X	X		X			X			X	X					X		X	X		X			X	X			X		X	X	X	X	X		X
13_1_40CM_68_15	X	X		X			X			X		X	X	X					X		X	X		X			X			X	X	X	X		X	X	X	X	X
13_1_40CM_69_27	X	X		X	X		X			X			X	X					X		X	X		X			X	X		X	X	X	X	X	X	X	X		X
13_1_40CM_69_96	X	X			X		X					X	X	X			X						X	X				X		X	X		X	X	X		X		
13_2_20CM_2_64_8	X	X			X	X	X			X			X	X			X		X		X			X			X	X	X	X	X	X	X			X	X	X	X
13_2_20CM_2_70_11	X	X			X		X					X		X			X		X			X		X				X	X	X	X	X	X		X	X			
13_2_20CM_69_10	X	X		X	X								X	X							X			X				X		X	X	X						X	X
13_2_20CM_69_15_1	X	X			X								X	X			X				X			X				X		X	X	X	X		X		X	X	X
13_2_20CM_69_15_2		X								X									X			X									X	X							X
13_2_20CM_70_12				X	X					X			X	X			X				X	X						X				X			X			X	
GWA2_70_23	X	X		X		X	X			X			X	X					X		X	X		X			X			X	X	X	X	X		X		X	X
GWA2_73_35	X	X		X	X		X			X			X	X					X		X		X	X			X	X		X	X	X	X	X	X		X	X	X
GWC2_70_16	X	X		X			X			X									X		X	X					X			X	X	X	X	X	X		X	X	X
GWC2_70_24	X	X			X	X	X					X									X	X		X			X	X		X	X	X			X				
GWF2_70_14	X	X				X	X														X	X					X			X	X	X			X	X			
RBG_16_73_20		X		X			X			X			X	X					X		X		X	X						X	X	X	X	X	X		X		
RIFCSPHIGHO2_02_FULL_69_13	X			X	X					X			X	X						X	X		X	X				X		X	X	X	X		X				
RIFCSPHIGHO2_02_FULL_73_26	X	X			X		X			X			X	X					X		X	X	X	X				X		X	X	X	X	X	X		X	X	X
RIFCSPHIGHO2_12_FULL_73_22		X	X	X	X		X			X			X	X					X		X	X	X	X				X		X	X	X	X	X			X		
RIFCSPLOWO2_02_FULL_68_19	X	X		X	X		X			X			X	X					X	X			X	X				X		X	X	X		X	X				X
RIFCSPLOWO2_02_FULL_71_18	X	X					X						X	X					X											X	X	X	X		X				
RIFCSPLOWO2_02_FULL_72_37	X	X			X								X	X					X		X	X	X	X			X	X		X	X	X			X			X	X
RIFCSPLOWO2_02_FULL_73_56		X		X	X		X			X			X	X					X		X	X	X	X				X		X	X	X	X	X			X		X
RIFCSPLOWO2_12_FULL_69_21		X		X	X		X						X	X					X	X	X		X					X		X	X	X	X			X	X	X	
RIFCSPLOWO2_12_FULL_71_19				X	X								X						X		X		X	X			X	X		X	X	X	X	X	X		X	X	
RIFCSPLOWO2_12_FULL_71_22	X	X		X	X								X	X					X		X	X	X	X			X	X		X	X	X	X		X		X		X
RIFCSPLOWO2_12_FULL_73_47	X	X		X	X		X			X			X	X					X		X	X	X	X				X		X	X	X	X	X	X		X		X
bacterium CSP1-6	X	X		X	X		X						X	X					X	X	X		X					X					X	X			X	X	

	Nitrogen Metabolism								Sulfur Metabolism								TCA Cycle																	Wood-Ljungdahl Pathway (acetogenesis) with associated steps of serine and methionine biosynthesis									
Rokubacteria Metagenome-Assembled Genome	nitrate reductase	nitrite reductase (NO-forming)	nitrite reductase	nitrilase	formamidase	glutamate-ammonia ligase	anaerobic nitric oxide reductase flavorubredoxin	aminomethyltransferase	sulfate adenylyltransferase	sulfate adenylyltransferase dissimilatory type	adenylylsulfate reductase	sulfite reductase	sulfite reductase dissimilatory (desulfoviridin)	adenylylsulfate kinase	phosphoadenylyl-sulfate reductase	sulfur oxidation protein SoxB	malate dehydrogenase	Isocitrate dehydrogenase (NAD(+))	Isocitrate dehydrogenase (NADP(+))	pyruvate dehydroegnase (acetyl-transferring)	oxoglutarate dehydrogenase (succinyl-transferring)	pyruvate synthase	2-oxoglutarate synthase	succinate dehydrogenase (ubiquinone)	succinate dehydrogenase	dihydrolipoyl dehydrogenase	dihydrolipoyllysine-residue acetyltransferase	citrate (Si)-synthase	phosphoenolpyruvate carboxykinase	fumarate hydratase	aconitate hydratase	succinate--CoA ligase (ADP-forming)	pyruvate carboxylase	carbon monoxide dehydrogenase	acetyl-CoA synthase	methyltransferase corrinoid activation protein	ferredoxin	formate dehydrogenase	formyl-tetrahydrofolate synthase	methylene-tetrahydrofolate deydrogenase/cyclohydrolase	serine hydroxymethyltransferase	methylene-tetrahydrofolate reductase	methionine synthase
Rokubacteria_Amazon_FNV_2010_15_13		X				X		X									X	X		X	X						X	X	X		X			X	X		X		X	X			X
RIFCSPHIGHO2_12_FULL_73_22	X	X						X									X		X	X		X	X	X	X	X	X	X		X	X	X		X	X	X	X	X	X	X		X	
RIFCSPLOWO2_02_FULL_69_13	X					X		X		X	X					X	X		X	X		X	X	X		X	X	X		X	X	X		X	X			X	X	X	X		
RISCPLOWO2_12_FULL_68_19	X				X	X		X		X	X					X	X		X	X		X	X	X	X	X	X	X		X	X	X		X	X		X		X	X	X	X	
RIFCSPLOWO2_12_FULL_69_21	X		X			X		X		X	X					X	X		X	X		X	X	X	X	X	X	X		X	X	X		X	X			X	X			X	
RIFSCPLOWO2_12_FULL_71_18			X								X						X		X			X	X	X						X	X	X		X			X				X		
RIFCPLOWO2_12_FULL_71_19	X	X				X				X	X					X				X		X	X	X	X	X	X	X	X		X	X		X	X			X	X	X		X	X
RIFCSPLOWO2_12_FULL_71_22	X	X				X	X		X		X	X		X		X			X	X		X	X	X		X	X	X	X	X	X			X	X		X	X	X	X	X	X	
RIFCSPLOWO2_12_FULL_72_37	X		X											X			X			X		X	X		X	X	X	X	X		X			X	X	X	X	X	X	X	X		
RIFSCPLOWO2_12_FULL_73_26	X	X				X	X	X									X		X	X			X	X	X	X	X	X		X	X	X		X	X		X	X	X	X	X	X	
RIFCSPLOWO2_12_FULL_73_47	X	X				X	X	X									X		X	X		X	X	X	X	X	X	X		X	X	X		X	X	X	X	X	X	X	X	X	
RIFCPLOWO2_12_FULL_73_56	X	X				X	X	X									X		X	X			X	X	X	X	X	X		X	X	X		X	X	X	X	X	X	X		X	
13_1_20CM_2_68_19	X							X									X									X	X	X		X	X	X		X		X	X	X					X
13_1_20CM_2_69_58	X		X		X			X									X		X	X		X		X		X	X	X		X	X	X		X			X		X	X	X	X	X
13_1_20CM_2_70_7								X		X			X			X	X		X	X				X	X				X	X	X			X			X	X			X	X	
13_1_20CM_4_68_9	X			X		X	X										X		X	X				X	X	X	X	X	X	X	X	X		X	X		X	X		X	X	X	X
13_1_20CM_4_70_13																	X		X						X				X	X	X			X			X						
13_1_20CM_4_70_14	X				X			X								X	X			X		X				X	X	X		X	X			X				X			X	X	X
13_1_20CM_70_15																										X								X			X	X		X	X		X
13_1_40CM_2_68_13	X			X													X			X				X		X	X	X	X	X	X	X	X	X				X					X
13_1_40CM_2_68_8																	X			X		X		X		X	X	X	X	X			X	X			X	X	X		X		X
13_1_40CM_2_70_45	X							X					X			X	X			X		X					X	X		X				X							X		
13_1_40CM_3_69_38	X		X		X											X	X		X	X		X				X	X	X		X	X		X	X					X		X	X	X
13_1_40CM_4_67_11	X			X		X											X			X				X		X	X	X	X	X	X			X			X	X			X		
13_1_40CM_4_69_39	X				X			X								X	X		X	X						X	X	X		X	X	X		X				X	X		X	X	X
13_1_40CM_4_69_5						X		X		X					X	X	X		X	X		X		X	X	X	X	X	X	X	X	X		X	X		X	X		X	X		X
13_1_40CM_68_15	X		X	X		X	X			X			X			X	X			X		X				X	X	X	X	X	X			X	X	X	X	X		X	X	X	X
13_1_40CM_69_27						X		X		X			X		X	X	X		X	X		X		X	X	X	X	X	X	X	X	X		X			X	X		X	X	X	X
13_1_40CM_69_96								X					X				X			X		X		X		X	X	X		X	X	X		X			X		X	X	X	X	
13_2_20CM_2_64_8	X			X		X		X									X		X	X				X		X		X	X	X	X			X	X		X	X		X	X	X	X
13_2_20CM_2_70_11						X		X					X			X	X		X	X		X		X		X	X	X		X	X	X		X			X			X	X	X	X
13_2_20CM_69_10	X							X					X			X				X				X		X		X		X	X	X		X	X		X	X		X	X	X	X
13_2_20CM_69_15_1						X		X								X	X			X						X	X	X		X	X			X		X	X	X		X	X		X
13_2_20CM_69_15_2							X						X			X				X						X	X			X		X		X									
13_2_20CM_70_12	X				X			X								X				X		X					X	X		X	X			X				X					X
GWA2_70_23	X	X				X		X		X	X					X	X		X	X		X	X	X	X	X	X	X	X	X	X	X		X	X		X	X		X	X		
GWA2_73_35	X	X						X						X			X		X	X		X	X	X	X	X	X	X	X	X	X	X		X	X		X	X	X	X	X		
GWC2_70_16	X	X				X				X	X					X	X		X	X		X	X	X	X	X	X	X	X	X	X	X		X	X			X	X			X	X
GWC2_70_24	X	X				X		X									X		X	X		X	X	X	X	X	X	X	X	X	X	X		X	X			X		X	X		
GWF2_70_14	X	X				X				X	X						X		X	X		X	X	X	X	X	X	X	X	X	X	X		X	X		X	X			X	X	
RBG_16_73_20	X	X				X								X			X		X	X		X	X	X	X	X	X	X		X	X	X		X	X			X	X	X			
RIFCSPHIGHO2_02_FULL_69_13	X					X		X		X	X					X	X		X	X		X	X	X		X	X	X		X	X	X		X	X	X	X	X	X	X	X		
RIFCSPHIGHO2_02_FULL_73_26	X	X				X	X	X									X		X	X			X	X	X	X	X	X		X	X	X		X		X	X	X	X	X	X	X	
RIFCSPHIGHO2_12_FULL_73_22	X	X						X									X		X	X		X	X	X	X	X	X	X		X	X	X		X		X	X	X	X	X		X	
RIFCSPLOWO2_02_FULL_68_19	X				X	X		X		X	X					X	X		X	X		X	X	X	X	X	X	X		X	X	X		X	X		X		X	X	X	X	
RIFCSPLOWO2_02_FULL_71_18			X								X						X		X			X	X	X						X	X	X		X			X				X		
RIFCSPLOWO2_02_FULL_72_37	X		X											X			X			X		X	X		X	X	X	X	X		X			X	X		X	X	X	X	X		
RIFCSPLOWO2_02_FULL_73_56	X	X				X	X	X											X	X			X	X	X	X	X	X		X	X	X		X		X	X	X	X	X		X	
RIFCSPLOWO2_12_FULL_69_21	X		X			X		X		X	X					X	X		X	X		X	X	X	X	X	X	X		X	X	X		X	X			X				X	
RIFCSPLOWO2_12_FULL_71_19	X	X				X				X	X	X				X				X		X	X	X	X	X	X	X	X		X	X		X	X	X		X	X	X		X	X
RIFCSPLOWO2_12_FULL_71_22	X	X				X	X		X		X			X					X	X		X	X	X		X	X	X	X	X	X			X	X		X	X	X	X	X	X	
RIFCSPLOWO2_12_FULL_73_47	X	X				X	X	X									X		X	X		X	X	X	X	X	X	X		X	X	X		X		X	X	X	X	X	X	X	
bacterium CSP1-6	X					X				X	X					X	X		X	X		X	X	X	X	X	X	X		X	X	X		X	X		X	X			X		
